# A comprehensive assessment of somatic mutation detection in cancer using whole-genome sequencing

**DOI:** 10.1038/ncomms10001

**Published:** 2015-12-09

**Authors:** Tyler S. Alioto, Ivo Buchhalter, Sophia Derdak, Barbara Hutter, Matthew D. Eldridge, Eivind Hovig, Lawrence E. Heisler, Timothy A. Beck, Jared T. Simpson, Laurie Tonon, Anne-Sophie Sertier, Ann-Marie Patch, Natalie Jäger, Philip Ginsbach, Ruben Drews, Nagarajan Paramasivam, Rolf Kabbe, Sasithorn Chotewutmontri, Nicolle Diessl, Christopher Previti, Sabine Schmidt, Benedikt Brors, Lars Feuerbach, Michael Heinold, Susanne Gröbner, Andrey Korshunov, Patrick S. Tarpey, Adam P. Butler, Jonathan Hinton, David Jones, Andrew Menzies, Keiran Raine, Rebecca Shepherd, Lucy Stebbings, Jon W. Teague, Paolo Ribeca, Francesc Castro Giner, Sergi Beltran, Emanuele Raineri, Marc Dabad, Simon C. Heath, Marta Gut, Robert E. Denroche, Nicholas J. Harding, Takafumi N. Yamaguchi, Akihiro Fujimoto, Hidewaki Nakagawa, Víctor Quesada, Rafael Valdés-Mas, Sigve Nakken, Daniel Vodák, Lawrence Bower, Andrew G. Lynch, Charlotte L. Anderson, Nicola Waddell, John V. Pearson, Sean M. Grimmond, Myron Peto, Paul Spellman, Minghui He, Cyriac Kandoth, Semin Lee, John Zhang, Louis Létourneau, Singer Ma, Sahil Seth, David Torrents, Liu Xi, David A. Wheeler, Carlos López-Otín, Elías Campo, Peter J. Campbell, Paul C. Boutros, Xose S. Puente, Daniela S. Gerhard, Stefan M. Pfister, John D. McPherson, Thomas J. Hudson, Matthias Schlesner, Peter Lichter, Roland Eils, David T. W. Jones, Ivo G. Gut

**Affiliations:** 1 CNAG-CRG, Centre for Genomic Regulation, Barcelona Institute of Science and Technology (BIST), Baldiri i Reixac 4, 08028 Barcelona, Spain; 2 Universitat Pompeu Fabra (UPF), 08002 Barcelona, Spain; 3 Division of Theoretical Bioinformatics, German Cancer Research Center, Im Neuenheimer Feld 280, Heidelberg 69120, Germany; 4 Division of Applied Bioinformatics, German Cancer Research Center, Im Neuenheimer Feld 280, Heidelberg 69120, Germany; 5 Cancer Research UK Cambridge Institute, University of Cambridge, Li Ka Shing Centre, Robinson Way, Cambridge CB2 0RE, UK; 6 Department of Tumor Biology, Institute for Cancer Research, Oslo University Hospital, 0424 Oslo, Norway; 7 Department of Informatics, University of Oslo, 0373 Oslo, Norway; 8 Ontario Institute for Cancer Research, 661 University Avenue, Suite 510, Toronto, Ontario, Canada M5G 0A3; 9 Synergie Lyon Cancer Foundation, Centre Léon Bérard, Cheney C, 28 rue Laennec, Lyon 69373, France; 10 Queensland Centre for Medical Genomics, Institute for Molecular Bioscience, University of Queensland, St Lucia, Brisbane, Queensland 4072, Australia; 11 QIMR Berghofer Medical Research Institute, Brisbane, Queensland 4006, Australia; 12 Department of Genetics, Stanford University, Mail Stop-5120, Stanford, California 94305-5120, USA; 13 Genome and Proteome Core Facility, German Cancer Research Center, Im Neuenheimer Feld 280, Heidelberg, 69120 Germany; 14 Department of Pediatric Hematology and Oncology, Heidelberg University Hospital, Im Neuenheimer Feld 430, Heidelberg 69120, Germany; 15 Department of Neuropathology, Heidelberg University Hospital, Im Neuenheimer Feld 224, Heidelberg 69120, Germany; 16 Wellcome Trust Sanger Institute, Hinxton, Cambridge CB10 1SA, UK; 17 RIKEN Center for Integrative Medical Sciences, 4-6-1 Shirokanedai, Minato-ku, Tokyo 108-8639, Japan; 18 Universidad de Oviedo—IUOPA, C/Fernando Bongera s/n, 33006 Oviedo, Spain; 19 The Bioinformatics Core Facility, Institute for Cancer Genetics and Informatics, Oslo University Hospital, 0310 Oslo, Norway; 20 Victorian Life Sciences Computation Initiative, The University of Melbourne, Melbourne, Victoria 3053, Australia; 21 WolfsonWohl Cancer Research Centre, Institute of Cancer Sciences, University of Glasgow, Glasgow, Scotland G61 1QH, UK; 22 Knight Cancer Institute, Oregon Health and Science University, Portland, Oregon 97239-3098, USA; 23 BGI-Shenzhen, Shenzhen 518083, China; 24 The Genome Institute, Washington University, St Louis, Missouri 63108, USA; 25 Harvard Medical School, Boston, Massachusetts 02115, USA; 26 MD Anderson Cancer Center, Houston, Texas 77030, USA; 27 ^27^McGill University, Montreal, Quebec, Canada QC H3A 0G4; 28 Center for Biomolecular Science and Engineering, University of California, Santa Cruz, California 95064, USA; 29 IRB-BSC Joint Research Program on Computational Biology, Barcelona Supercomputing Center, 08034 Barcelona, Spain; 30 Human Genome Sequencing Center, Baylor College of Medicine, One Baylor Plaza, Houston, Texas 77030, USA; 31 Hematopathology Unit, Department of Pathology, Hospital Clinic, University of Barcelona, Institut d'Investigacions Biomèdiques August Pi i Sunyer, 08036 Barcelona, Spain; 32 Department of Medical Biophysics, University of Toronto, Toronto, Ontario, Canada M5G 1L7; 33 National Cancer Institute, Office of Cancer Genomics, 31 Center Drive, 10A07, Bethesda, Maryland 20892-2580, USA; 34 Division of Pediatric Neurooncology, German Cancer Research Center (DKFZ), Im Neuenheimer Feld 280, Heidelberg 69120, Germany; 35 Department of Molecular Genetics, University of Toronto, Toronto, Ontario, Canada M5S 1A8; 36 Division of Molecular Genetics, German Cancer Research Center (DKFZ), Im Neuenheimer Feld 280, Heidelberg 69120,Germany; 37 Heidelberg Center for Personalised Oncology (DKFZ-HIPO), German Cancer Research Center (DKFZ), Heidelberg, Germany; 38 Institute of Pharmacy and Molecular Biotechnology, University of Heidelberg, Heidelberg 69120, Germany; 39 Bioquant Center, University of Heidelberg, Im Neuenheimer Feld 267, Heidelberg 69120, Germany; 40 Division of Pediatric Neurooncology, German Cancer Research Center (DKFZ), Im Neuenheimer Feld 280, Heidelberg 69120, Germany

## Abstract

As whole-genome sequencing for cancer genome analysis becomes a clinical tool, a full understanding of the variables affecting sequencing analysis output is required. Here using tumour-normal sample pairs from two different types of cancer, chronic lymphocytic leukaemia and medulloblastoma, we conduct a benchmarking exercise within the context of the International Cancer Genome Consortium. We compare sequencing methods, analysis pipelines and validation methods. We show that using PCR-free methods and increasing sequencing depth to ∼100 × shows benefits, as long as the tumour:control coverage ratio remains balanced. We observe widely varying mutation call rates and low concordance among analysis pipelines, reflecting the artefact-prone nature of the raw data and lack of standards for dealing with the artefacts. However, we show that, using the benchmark mutation set we have created, many issues are in fact easy to remedy and have an immediate positive impact on mutation detection accuracy.

## Abstract (web summary)

Cancer genetics has benefited from the advent of next generation sequencing, yet a comparison of sequencing and analysis techniques is lacking. Here, the authors sequence a normal-tumour pair and perform data analysis at multiple institutes and highlight some of the pitfalls associated with the different methods.

## Graphical abstract

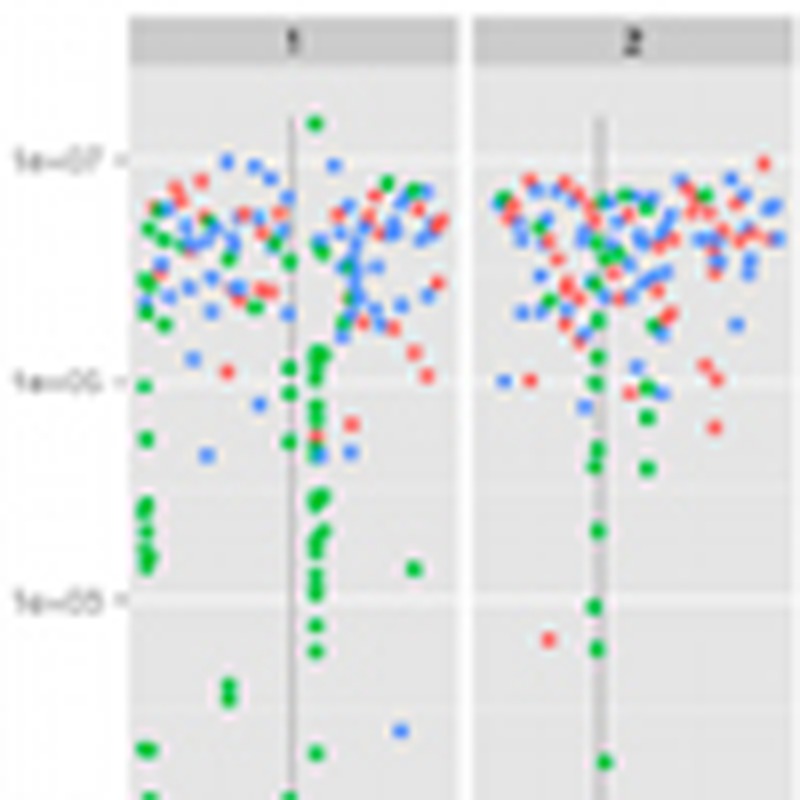

The International Cancer Genome Consortium (ICGC) is characterizing over 25,000 cancer cases from many forms of cancer^1^. Currently, there are 74 projects supported by different national and international funding agencies. As innovation and development of sequencing technologies have driven prices down and throughput up, projects have been transitioning from exome to whole-genome sequencing (WGS) of tumour and matched germline samples, supplemented by transcript and methylation analyses when possible, facilitating the discovery of new biology for many different forms of cancer^2^,^3^,^4^,^5^,^6^,^7^,^8^,^9^,^10^. However, as data from the different projects began to be collected and centralized (https://dcc.icgc.org/), it became apparent that there are marked differences in how teams generate WGS data and analyse it. On the basis of cost, capacity and analytical experience, it was initially determined that comprehensive identification of tumour-specific somatic mutations requires WGS with a minimum of 30 × sequence coverage of each the tumour and normal genomes^11^ with paired reads on the order of 100–250 bp in length, depending on the platform. However, from project to project the sample preparation, coverage of tumour and normal samples and read lengths vary. Even more variability exists in the approaches to identify differences between tumour and normal genomes, evidenced by the many strategies developed to identify somatic single-base mutations (SSM)^12^, somatic insertion/deletion mutations (SIM) and larger structural changes (rearrangements and chromosome segment copy number changes)^5^.

This variation makes comparison of mutation calls across cancers challenging because of the unknown contributions of individual pipeline components and parameters on the accuracy of the calls. Benchmark data sets and analytical tools^13^,^14^,^15^,^16^,^17^ have been developed for variant calling on normal genomes, while those for cancer have largely focused on SSM detection from exome sequencing^12^,^18^. Benchmarking of mutation calling from exome data from The Cancer Genome Atlas has raised concerns about biased inferences and highlights the need for benchmark data sets^19^. In our study we set out to investigate the factors that need to be considered to generate high-quality whole-genome sequence data and high-confidence variant calls from tumour-normal pairs, including new sources of bias and pitfalls not encountered in exome data.

We explored several benchmarking strategies. First, we evaluated somatic mutation calling pipelines using a common set of 40 × WGS reads of average quality corresponding to a case of chronic lymphocytic leukaemia (CLL). In a second benchmark, we evaluated both sequencing methods and somatic mutation calling pipelines using matched samples from a case of medulloblastoma (MB, a malignant pediatric brain tumour arising in the cerebellum^20^,^21^) from the ICGC PedBrain Tumor project. Both cancers exhibit a high degree of tumour purity (95–98%). For each case, we made available unaligned sequence reads of a tumour (∼40 × genome coverage) and its corresponding normal genome (∼30 × coverage) to members of the ICGC consortium, who then returned somatic mutation calls. In contrast to the approach taken in a recent benchmark of SSM calling using three simulated tumour genomes^22^, we have used the sequence from a real tumour-normal pair and made a concerted effort to manually curate both SSMs and SIMs detectable at a sequencing depth 8–10 times in excess of the standard amount (∼300 ×). We argue that real, not simulated, mutations are more useful for dissecting performance of mutation callers with respect to real genome-wide mutational signatures, and methods for detecting insertion–deletion mutations, an even bigger challenge to somatic mutation callers, must also be benchmarked. Our study has two main results: one, we identify outstanding issues in somatic mutation analysis from WGS data and begin to formulate a set of best practices to be adopted more widely by genome researchers, and, two, we provide two benchmark data sets for testing or developing new somatic mutation calling pipelines.

## Results

### WGS data generation

We conducted a first benchmark exercise using WGS data generated from a CLL tumour-normal pair and then a second using a case of MB. Both tumour types were expected to have relatively low mutational load and not very pervasive structural changes: CLL has a few known translocations and large copy number variants, and MB exhibits a large degree of tetraploidy but is otherwise typically free of complex rearrangements. The quality of the CLL data was below today's standards but typical for the time it was produced, while the quality of the MB library preparation and produced sequence were of high quality. The validation strategies also differed. For CLL we chose to validate submitted mutations by target capture and sequencing with two independent platforms (MiSeq and IonTorrent). This approach was limited by technical issues inherent to target capture and sequencing on these other platforms, which led to a low rate of independently validated mutations. Moreover, the real false-negative (FN) rates were underestimated because of the limited coverage provided to participants. For the MB data set, ∼300 × in sequence reads were generated by five different sequencing centres, which we joined and used to create a curated set of mutations. The MB tumour presented with a tetraploid background combined with other changes of ploidy in chromosomes 1, 4, 8, 15 and 17 (Supplementary Fig. 1), giving us the opportunity to benchmark performance at lower mutant allele frequencies. Moreover, the high depth and relatively unbiased coverage of the genome enabled higher sensitivity in mutation detection leading to a more inclusive set of curated mutations. For these reasons, we present here only the results for the MB benchmark.

### Evaluation of sequencing library construction methods

**Figure 1:**
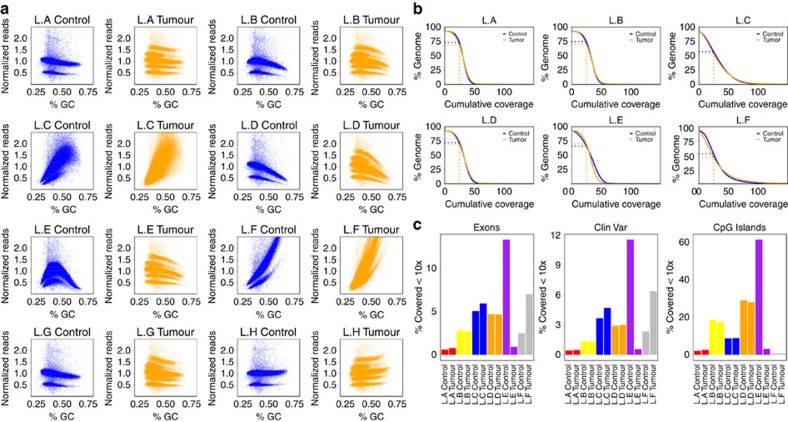
Differences between the different sample libraries. Libraries A, E and G are PCR-free. (**a**) GC bias of the different libraries. The genome was segmented into 10-kb windows. For each window, the GC content was calculated and the coverage for the respective library was added. For better comparability, the coverage was normalized by dividing by the mean. The major band in normal corresponds to autosomes, while the lower band corresponds to sex chromosomes. The increased number of bands in the tumour is because of a higher number of ploidy states in the (largely) tetraploid tumour sample. (**b**) Cumulative coverage displayed for different libraries. Displayed are all libraries sequenced to at least 28 ×. To make the values comparable, we downsampled all samples to a coverage of 28 × (the lowest coverage of the initially sequenced libraries). The plot shows the percentage of the genome (*y* axis) covered with a given minimum coverage (*x* axis). (**c**) Percentage of certain regions of interest covered with less than 10 ×. Different colours are used to distinguish centres.

Several different protocols were used for generating sequencing libraries at the five contributing sequencing centres, which varied in their reagent supplier, methods for selecting the fragment size of library inserts and use of amplifying PCR steps (Table 1 and Supplementary Table 1). Interestingly, these differences resulted in marked variation in the evenness of coverage genome-wide as well as in key regions of interest such as exons. PCR-free libraries were found to give the most even coverage, with very little effect of GC content on coverage levels (Fig. 1a). Evenness is directly correlated with coverage of the genome: when we downsampled each data set to an average 30 × tumour and control coverage (libraries sequenced to less than 28 ×, L.G and L.H, were excluded from further analysis), we see that in the best-performing libraries (L.A and L.B controls), 73–74% of the genome was covered at or above 25 ×, while the worst-performing library (L.F tumour) had only 46% of the genome covered at this level (Fig. 1b). In general, the coverage distribution was more even and the percentage of well-covered regions was higher in the control libraries compared with the tumour libraries, reflecting the different copy number states of the tumour. An unusual pattern of GC content distribution in control library E, however, meant that this was slightly worse than its tumour counterpart. The percentage of exonic regions covered at ≤10 × (that is, likely insufficient to accurately call mutations) also varied, with a range from less than 1% ‘missing' in the best-performing libraries to more than 10% in the worst (Fig. 1c), demonstrating that sequencing library preparation performance can have a significant impact on the ability to identify coding variants in downstream analyses. Performance in other regions of interest, such as enhancers and untranslated repeats, was similarly variable (Fig. 1c and Supplementary Fig. 2).

### Evaluation of sequencing depth

**Figure 2:**
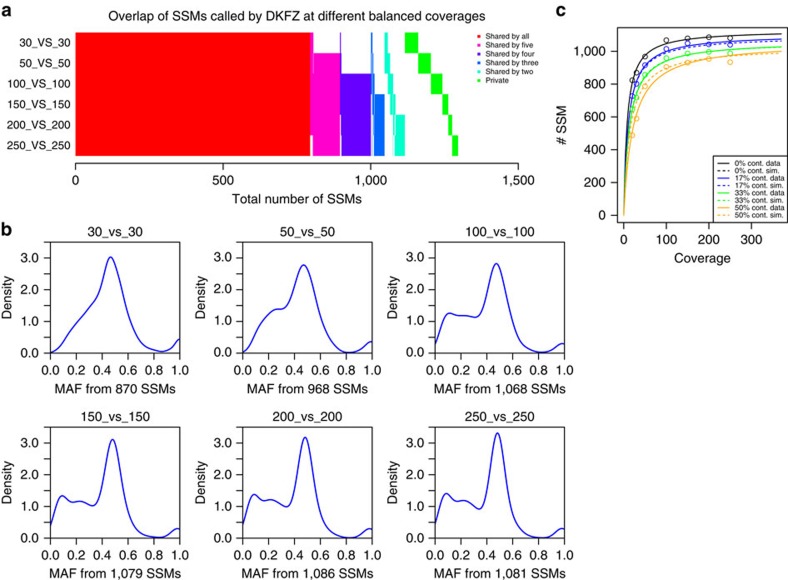
Effect of sequencing coverage on the ability to call SSMs. (**a**) Overlap of SSMs called on different balanced coverages. (**b**) Density plots of the variant allele frequencies for different balanced coverages of tumour and control (tumour_versus_control) and number of SSMs called in total (calls were performed using the DKFZ calling pipeline, MB.I). (**c**) Plot of the number of SSMs (*y* axis) found for a given coverage (*x* axis). The different colours represent different levels of normal ‘contamination' in the tumour (0% black, 17% blue, 33% green and 50% orange). Solid lines represent the real data and dashed lines are simulated. Lines are fitted against the Michaelis–Menten model using the ‘drc' package in R. Solid lines are fitted to the data points and dashed lines are simulated using a mixed inhibition model for enzyme kinetics.

Combining the sequencing data generated from each participating centre gave us the opportunity to investigate a tumour-normal pair with very deep coverage. After merging each of the individual pairs, the combined tumour coverage was 314 ×, and the control 272 ×. To remove already identified artefacts (Supplementary Note 1), we excluded the tumour library from centre E and the slightly contaminated control library from centre B. For comparison of mutation-calling metrics at a range of coverage levels, the combined tumour and normal sets were randomly serially downsampled to 250, 200, 150, 100, 50, 30 and 20 × coverage and then analysed using the standard DKFZ pipeline (MB.I, Supplementary Methods). The total number of mutations increases when going from 30 × to 50 × and further to 100 × coverage; however, no striking increase is seen above this level (at 100 ×, 95% of the maximum mutation number are detected, in contrast to only 77% at 30 × ; Fig. 2a and Supplementary Table 2). While the majority of mutations were called at the 30 × level, there were some notable differences in the number and type of mutations detected as the coverage increased. The sensitivity for detecting mutations with lower mutant allele frequencies (that is, subclonal alterations and/or events happening after polysomic changes but also major somatic mutations in samples with low tumour cell content) was much greater with higher coverage, as seen from density plots of mutations versus allele frequency (AF, Fig. 2b). This effect was even more striking when considering mutation calls per chromosome, which clearly shows the difference between low and high coverage when looking for late-occurring mutations after whole-chromosome copy number changes (Supplementary Fig. 3).

### Effect of tumour purity on mutation calling

Since MBs tend to show a very high tumour cell content (usually above 95%, and for this sample ∼98%, because of their nature as masses of small, round, tightly packed tumour cells), the high coverage data set also provided an opportunity to model the dynamics of mutation calling with increasing coverage and with increasing proportions of ‘contaminating' normal tissue. We found that the mutation calls with increasing coverage were accurately modelled with a Michaelis–Menten equation, reaching ‘saturation' (no or minimal additional mutations called as coverage increases) at around 100 × (Fig. 2c). The impact of normal cells on SSM detection could be thought of as a ‘mixed-type inhibition' of mutation detection sensitivity, which we examined by mixing increasing proportions of normal sequence reads (17, 33 and 50%) into the tumour data set and re-calling mutations. Each curve displayed the same plateau after ∼100 × as the pure tumour sample; however, the addition of any normal content meant that the maximum mutation count from the pure tumour could not be reached, even at 250 × total coverage. At 100 ×, the detected proportions of mutation calls from the pure sample were 95%, 90% and 85%, respectively, for 17%, 33% and 50% ‘contamination' (Fig. 2c). At lower coverage, the normal cell content had a proportionally larger impact. At 30 ×, only 92%, 83% or 68% of the calls from the 30 × pure sample were called when adding 17%, 33% or 50% normal reads, respectively (Supplementary Table 2). For SIMs called using the DKFZ pipeline, a different picture was observed. SIM calling at present likely suffers at least as much from low specificity as from low sensitivity, as indicated by the fact that increasing coverage actually reduces the number of called variants (that is, the FP rate decreases; Supplementary Fig. 4).

### Effect of tumour to normal sequencing depth ratio

We investigated the effect of tumour-normal coverage ratios on variant calling to assess whether increasing coverage of the tumour alone is sufficient to increase mutation detection sensitivity. The 250 × tumour genome was therefore compared with control genomes at 200, 150, 100, 50 and 30 × coverages. Down to the 150 × level, few differences are seen in the mutations called when compared with the 250 × /250 × standard (Supplementary Fig. 5a,b). At lower control coverage levels, a notable increase is observed in the overall number of mutations reported because of a sharp rise in those called with a low allele fraction. Since these mutations are not called in the 250 × versus 250 × set, it is almost certain that they are sequencing artefacts arising in a very small proportion of calls, which appear to be somatic when the control coverage is insufficient to show the same phenomenon. These new calls are dominated by a T>G base change arising in a particular sequence context (GpTpG, Supplementary Fig. 5c). Indeed, performing a motif analysis on the wider context of these changes revealed that the majority occur at a thymine base within a homopolymer run of guanines (Supplementary Fig. 5d). Keeping the ratio of tumour:normal coverage closer to one therefore appears to play a role in maintaining the accuracy of mutation calling with standard pipelines, since any systematic artefacts are then balanced out in both the tumour and control data sets. While it may be possible to apply additional filters to account for the new false positives (FPs) seen in unbalanced comparisons, this would potentially come at the cost of a reduced sensitivity for detecting true mutations with low allele frequencies (that is, tumour subpopulations), which are of particular interest when increasing sequencing coverage depth.

### Curation of a Gold Set of somatic mutations

We used the high-coverage (314 × :272 ×) data set from the sequencing benchmark to curate a Gold Set of verified somatic mutations (Methods). Gold Set mutations were classified (Table 2) according to the potential issues that may lead to an incorrect call: Tier 1 mutations have a mutant AF≥10%, Tier 2 mutations have AF≥5%, Tier 3 includes all verified mutations supported by unambiguous alignments, while Tier 4 includes additional mutations with more complicated or ambiguous local alignments and Tier 5 includes those with unusually high or low read depth.

The MB Gold Set had a total of 1,620 bona fide mutation calls across all tiers (Table 2), with 962, 1,101, 1,255 and 1,263 SSMs in Tiers 1, 2, 3 and 4, respectively, and 337 and 347 SIMs in Tiers 1 and 4, respectively. The mutational load of this tumour was ∼0.5 mutations per Mbp. Of these, there were eleven exonic SSMs (seven missense, three synonymous and one early stop) and one splice site mutation. We found that 32% of SSMs are in RepeatMasked sequence, 9% in tandem repeats (4% in homopolymer tracts) and 4.4% adjacent to tandem repeats. About a quarter of SSMs (27%) exhibits a mutant AF in the tumour of less than 10%, with 6% being very close to the alternate AF in the normal sample (Supplementary Table 3). For curated SIMs, 83% fall in tandem repeats (71% in homopolymers; Supplementary Table 4).

### Evaluation of somatic mutation calling pipelines

A submission and revision process was set up for the MB benchmark with guidelines for the mutation call format. Participating centres were provided with the best-quality sequence data set from the sequencing benchmark (L.A). Using these FASTQs, they produced SSM and SIM calls and submitted them for evaluation. We received 18 SSM and 16 SIM submissions.

**Figure 3:**
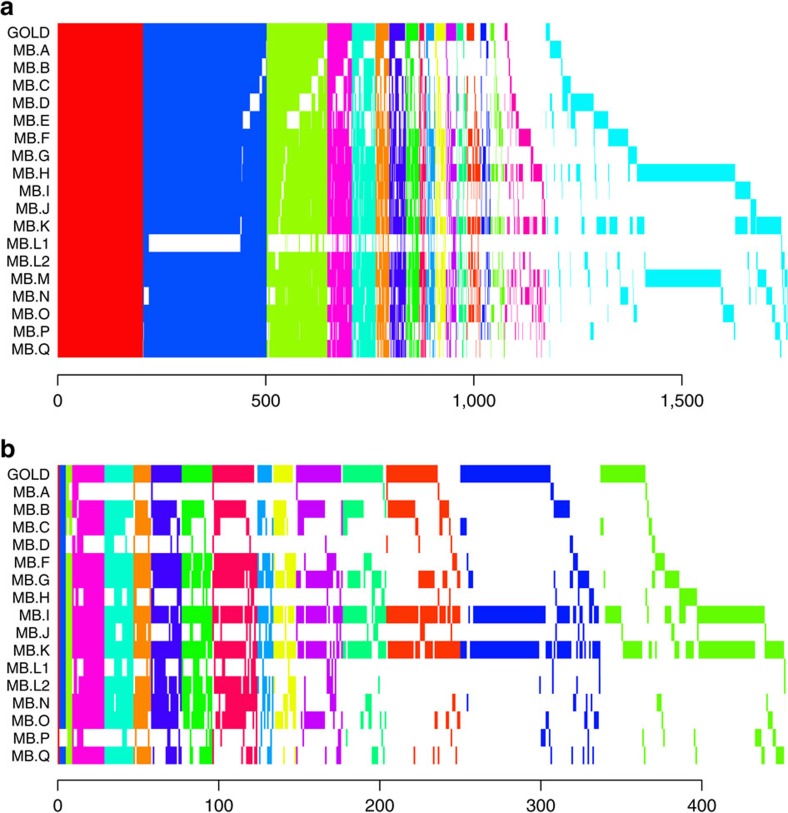
Overlap of somatic mutation calls for each level of concordance. Shared sets of calls are vertically aligned. GOLD indicates the Gold Set. (**a**) Medulloblastoma SSM calls shared by at least two call sets. (**b**) Medulloblastoma SIM calls shared by at least two call sets.

**Figure 4:**
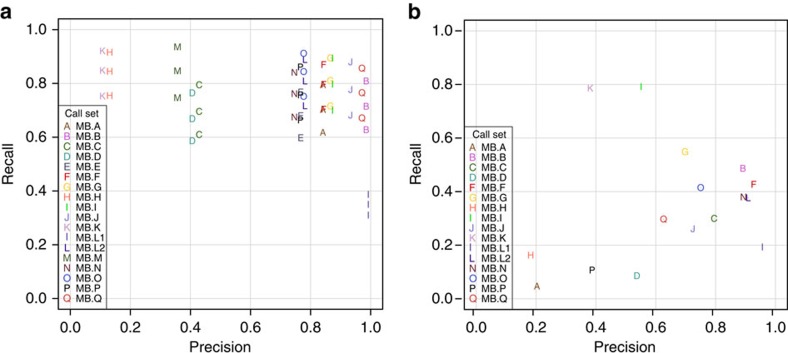
Somatic mutation calling accuracy against Gold Sets. Decreasing sensitivity on Tiers 1, 2 and 3 shown as series for each SSM call set, while precision remains the same. (**a**) Medulloblastoma SSMs. (**b**) Medulloblastoma SIMs.

Submissions were compared among themselves and to the Gold Set. Figure 3 shows the overlap of mutation call sets (private calls are shown in Supplementary Figs 6 and 7). We found that only 205 SSMs and one SIM were agreed upon by all submitters (Fig. 3 and Supplementary Figs 6 and 7). Agreement among SSM sets was much greater than agreement among SIMs in general. In Fig. 4 we show the precision versus recall of the submitted mutation calls. Each letter corresponds to a submission compared with the Gold Set of Tiers 1, 2 or 3, with comparison with Tier 1 (AF>10% mutant AF) having the highest value for recall in the plot, Tier 2 (AF>5%) the second highest and Tier 3 (AF>∼2%) the lowest. Precision is always calculated against Tier 4, which also includes mutations that are complex or have ambiguous positions.

We observed a cluster of well-performing SSM submissions with high values for both precision and recall. Those with the highest F1 scores (Table 3) were MB.Q and MB.J, pipelines that combine two different somatic mutation callers: qSNP^23^ with GATK^24^ and SGA^25^ with FreeBayes^26^ (Supplementary Methods). Submissions with a high number of calls did not necessarily achieve higher recall; about two-thirds of all mutations (or >80% of Tier 1 mutations) can be detected without making many false-positive (FP) calls, after which increases in recall are accompanied by precipitous declines in precision. This is because of the fact that at 40 × depth, a fraction of the curated mutations is impossible to detect. Likewise, the one submission whose precision was the highest (MB.L1) was not much more precise than MB.B or MB.Q, which both found over twice as many true mutations (Table 3). For SIMs (Fig. 4b), some submissions achieved precisions greater than 0.9; however, their sensitivities were still low. The highest F1 score (0.65 for MB.I) is noticeably lower than that obtained for SSMs (0.79). Overall, SIM detection appears to be more challenging, with performance lagging behind than that of SSM detection.

### Correlation of pipeline components with shared mutations

Could those submissions that cluster together in terms of precision and recall be calling the same mutations or have similarities in terms of their pipelines? Using a measure of pairwise overlap, the Jaccard index, we clustered the submissions and display the results as both a heatmap and hierarchical clustering in Supplementary Fig. 8. Correspondence analysis gave similar results. We also broke them down by true positives (TPs) or FPs (Supplementary Figs 9–12) and clustered the pipelines based on shared components or parameters (Supplementary Fig. 13, input data in Supplementary Data 1). We find that when submissions agree, they tend to agree on true mutations, and when they disagree, these are more likely to be FPs or true negatives. Some notable exceptions can be observed among the FP SIMs, where MB.L1 and MB.L2 cluster as do MB.F and MB.N. These concordant FPs may indicate incompleteness of the Gold Set and/or similarities in the pipelines. In this case, MB.L1 is a filtered subset of MB.L2, explaining the high degree of overlap. MB.F and MB.N both use Strelka^27^ to call SIMs, possibly explaining the overlap in FPs. Indeed, some overlap of MB.F and MB.N FP SIMs is seen with MB.L2, which also uses Strelka. For TP SIMs, pipelines that share components tend to have higher overlap, for example, among Strelka calls or among GATK SomaticIndelDetector calls. Sometimes, we observe higher Jaccard index values for pipelines using different software, for example Platypus^28^ and Atlas-indel^29^, which are two of the most sensitive mutation callers. There is much more concordance among SSM calls; therefore, trends are harder to see among the FPs. Logically, SSM submissions with the highest F1 scores have the highest Jaccard indices.

### Genomic or alignment features affecting accuracy

**Figure 5:**
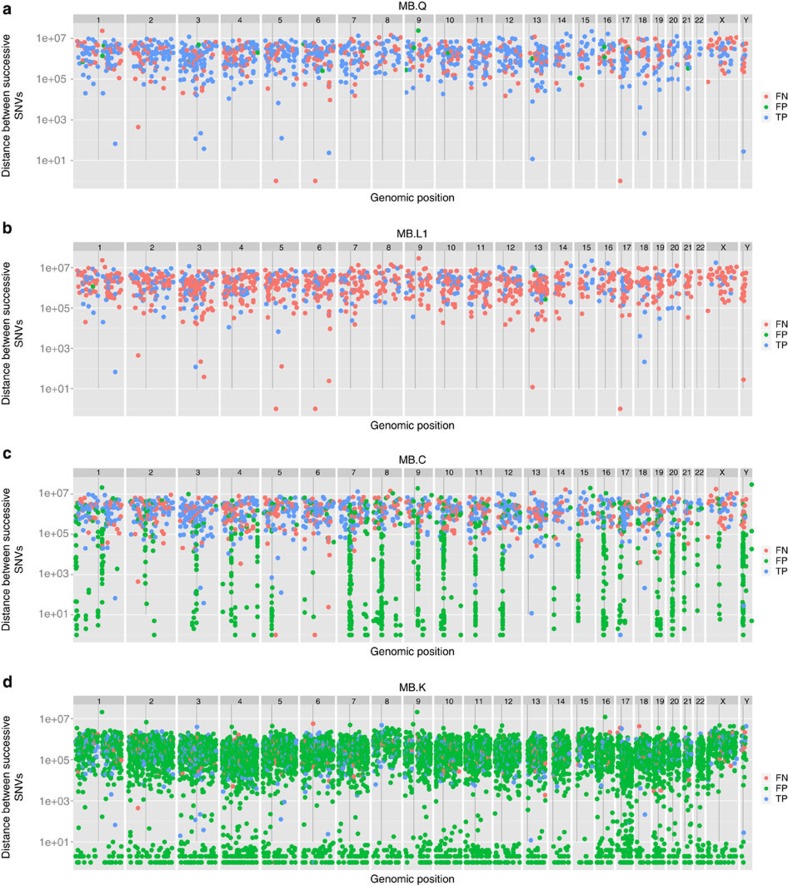
Rainfall plot showing distribution of called mutations on the genome. The distance between mutations is plotted in the log scale (*y* axis) versus the genomic position on the *x* axis. TPs (blue), FPs (green) and FNs (red). Four MB submissions representative of distinct patterns are shown. (**a**) MB.Q is one of best balanced between FPs and FNs, with low positional bias. (**b**) MB.L1 has many FNs. (**c**) MB.C has clusters of FPs near centromeres and FNs on the X chromosome. (**d**)MB.K has a high FP rate with short distance clustering of mutations.

We asked what genomic features or sample, sequence or alignment features might correlate with better or worse performance. We used ‘rainfall' plots that show density of features along a genome. By plotting the distance from the previous mutation (Fig. 5 and Supplementary Fig. 14), we can observe clustering or hotspots. SSM and SIM calls are coloured according to TP, FP and FN status. The Gold Set exhibits no mutational hotspots; therefore, any deviation is likely to be caused by a feature of the pipeline. Indeed, we detect quite different patterns: MB.Q (Fig. 5a) and MB.B (Fig. 5b) do not display any notable hotspots, while MB.C (Fig. 5c), for example, has many FP mutation calls in centromeric regions. MB.D and other call sets display varying degrees of this problem, which may arise if alignment of reads is performed to the GRCH37 reference without the d5 decoy sequence and/or no ultrahigh-signal blacklist is employed. MB.K overcalls (Fig. 5d) but a more subtle pattern is also apparent: higher ploidy chromosomes (for example, 17) display a greater density of calls and lower ploidy chromosomes (8, 15, X and Y) demonstrate a lower density of calls, presumably because of coverage.

**Figure 6:**
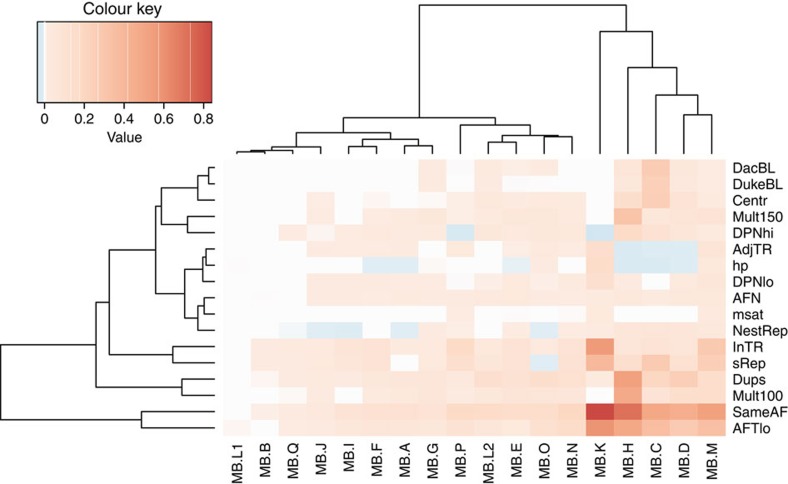
Enrichment or depletion of genomic and alignment features in FP calls for each medulloblastoma SSM submission. For each feature, the difference in frequency with respect to the Gold Set is multiplied by the FP rate. Blue indicates values less than zero and thus the proportion of variants or their score on that feature is lower in the FP set with respect to the true variants. Reddish colours correspond to a higher proportion of variants or higher scores for the feature in FP calls versus the Gold Set. Both features and submissions are clustered hierarchically. The features shown here include same AF (the probability that the AF in the tumour sample is not higher than that in the normal samples, derived from the snape-cmp-counts score), DacBL (in ENCODE DAC mappability blacklist region), DukeBL (in Encode Duke Mappability blacklist region), centr (in centromere or centromeric repeat), mult100 (1—mappability of 100mers with 1% mismatch), map150 (1—mappability of 150mers with 1% mismatch), DPNhi (high depth in normal), DPNlo (low depth in normal), dups (in high-identity segmental duplication), nestRep (in nested repeat), sRep (in simple repeat), inTR (in tandem repeat), adjTR (immediately adjacent to tandem repeat), msat (in microsatellite), hp (in or next to homopolymer of length >6), AFN (mutant AF in normal) and AFTlo (mutant AF in tumour<10%).

Other genomic features such as tandem or interspersed repeats, as well as some key sample/sequence/alignment features, also create problems for mutation callers but are not detectable at the chromosomal scale. We annotated the Gold Set and all submitted SSM and SIM calls for each feature, indicating membership (Boolean flags) or a score (for continuous characteristics; Supplementary Tables 3 and 4). The frequencies or mean scores, respectively, were computed for three subsets of each submission (TPs, FPs or FNs) and for the Gold Sets. To highlight problematic features for each submission, the differences with respect to the Gold Set were computed and multiplied by the FP or FN rate, accordingly. The problematic features of FPs in the MB SSM data set are shown as a heat map (Fig. 6). While nearly all sets of FPs are enriched in low-frequency mutations, which are harder to discriminate from background noise (also reflected by the ‘same AF' metric), some call sets (MB.K, H, C, D and M) do less well. MB.H seems to also have a problem with segmental duplications and multimappable regions, and MB.D with duplications only. MB.K and MB.M, to a lesser extent, are enriched in SSMs located in tandem repeats, simple repeats and homopolymers. MB.C has issues with FPs falling in blacklisted regions, specifically centromeric and simple repeats. The three submissions with fewer FPs immediately adjacent to tandem repeats than in the Gold Set (MB.H, MB.C and MB.D) do not use the Burrows-Wheeler Aligner (BWA) for the primary alignment step—instead, the mappers Novoalign or GEM are used, or the detection method is not based on mapping (SMUFIN^30^). The corresponding heatmaps for MB SSM FNs, and MB FP and FN SIMs are shown in Supplementary Figs 15–17. In general, both tandem repeats and segmental duplications and interspersed repeats cause sensitivity issues, with some pipelines more affected than others. The results for SIMs show that SIMs in tandem repeats (the majority being homopolymers) are undercalled, being under-represented in FPs and over-represented in FNs. Interestingly, nested repeats and duplications show the opposite trend, indicating that many FPs likely arise from low mapping quality.

Correspondence analysis confirms some of the above findings for MB SSM FPs (Supplementary Fig. 18). MB.C clusters with EncMap, dukeMap and centr, suggesting that MB.C FPs occur in some blacklisted regions. MB.H (and MB.G and MB.O to lesser extent) FPs are associated with segmental duplications. MB.K FPs are associated with tandem repeats (and microsatellites and simple repeats).

### Effect of mapper on mutation calling

The differences between sets of mutations submitted by the participating groups raised questions about the impact of individual pipeline components on the results. The extent of observed pipeline customization (Supplementary Methods and Supplementary Data 1) did not allow for exhaustive testing of all potentially important analysis steps; however, three pipeline components were selected for closer inspection because of their expected high impact: mapper, reference genome build and mutation caller. Four mappers (Novoalign2, BWA, BWA-mem and GEM), two SSM callers (MuTect^31^ and Strelka) and three versions of the human reference genome (b37, b37+decoy and ‘hg19r'—a reduced version of hg19, with unplaced contigs and haplotypes removed) were selected for testing, based on their usage by the benchmarking groups (Supplementary Methods for software versions and settings). To limit the effect of non-tested software on the produced mutation sets, a simple SSM-calling pipeline was established. First, we compared the effect of the mapper with each of the SSM callers. With a single SSM caller employed, a considerable fraction of unfiltered SSM calls for a given mapper (0.22–0.69, depending on the mapper–caller combination) is not reproducible by that caller with any other mapper (Supplementary Fig. 19). When compared with the Gold Set (Tier 3 SSMs), calls supported by a single mapper are almost exclusively FPs (precision <0.02). On the other hand, a large majority of calls supported by all four mappers are TPs (with precision ranging from 0.87 for MuTect to 0.99 for Strelka).

### Effect of primary mutation caller on mutation calling

Similar trends are observed when SSM callers are compared while holding the mapper constant (Supplementary Table 5). A sizable fraction (0.22–0.87, depending on the mapper) of unfiltered SSM calls for any given mapper–caller combination is not reproducible by the other caller on the same alignment file. Remarkably, in case of Novoalign2, the same alignment file leads to the most somatic calls and the lowest overall precision when used with MuTect, but the fewest somatic calls and highest overall precision when used with Strelka. When compared with the Gold Set, calls private to a single caller appear to be mostly FPs, with precision ranging from 0.01 to 0.05. Calls supported by both callers prove to be mostly correct (with precision between 0.89 and 0.93; Supplementary Table 6). The consensus sets seem to be robust—considerably improving the precision rates while only minimally lowering the sensitivity. The results of reference genome choice and a detailed examination of the alignment characteristics of the different aligners are presented in Supplementary Note 1.

### Improvement of pipelines using the benchmark data set

**Figure 7:**
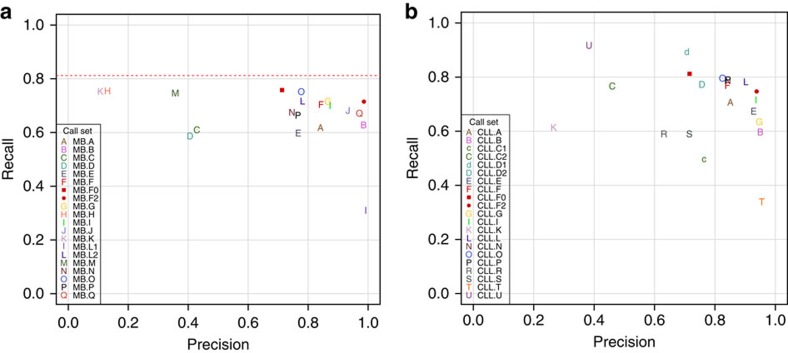
Accuracy of re-filtered pipeline SSM calls. Unfiltered calls (MB.F0 and CLL.F0) are shown as a red squares, while the calls using the tuned filters (MB.F2 and CLL.F2) are shown as red circles for the medulloblastoma (**a**) and CLL (**b**) benchmark GOLD sets. For MB, only the recall versus Tier 3 is shown. Overall, 1,019 (81.2%) of the medulloblastoma SSMs (indicated by the dotted line) are considered callable at 40 × coverage; 236 MB SSMs (18.2%) were not called by any pipeline. For CLL, verification was carried out on SSMs originally called on the 40 × data, which explains the higher recall.

As a demonstration of the utility of the benchmark data set to improve pipelines, we set out to improve the MB.F pipeline further (already the MB.E pipeline, which uses SomaticSniper, was replaced with the MB.F pipeline, which uses Strelka, based on the analysis of the CLL benchmark results). Using the Gold Set to devise and tune a set of filters for various metrics (Supplementary Table 7), including mapping quality and distance from the end of the read alignment block (Supplementary Fig. 20), we are able to outperform (in terms of F1) all other MB SSM submissions (Fig. 7a and Supplementary Table 8). Despite choosing reasonably conservative thresholds, we were still worried about the possibility of overfitting; thus, we tested the adjusted pipeline on the CLL benchmark data set. We achieved similar results (Fig. 7b), demonstrating that the filter settings work well on at least one other cancer type. Removal of the repeat copy filter in Strelka also improve both MB and CLL SIM sensitivity without greatly affecting precision (Supplementary Fig. 21 and Supplementary Table 8).

Additional information regarding the CLL benchmark is provided in Supplementary Note 1, Supplementary Figs 22–36 and Supplementary Tables 9–13, which present the analogous information presented here for MB. Additional sequencing analyses are described in Supplementary Note 1, Supplementary Figs 37–40 and Supplementary Tables 14 and 15. Controls for genome reference builds and effect of mapper choice are presented in Supplementary Note 1, Supplementary Figs 41–47 and Supplementary Table 16. All pipeline details (as given by each submitter) are presented in Supplementary Methods, Supplementary Fig. 48 and Supplementary Tables 17 and 18.

## Discussion

This benchmarking exercise has highlighted the importance of carefully considering all stages of the laboratory and analysis pipelines required to generate consistent and high-quality whole-genome data for cancer analysis. In this study we have isolated and tested individual library construction/sequencing methods and complete analysis pipelines. Analysis pipelines themselves are also multicomponent; therefore, we have also evaluated mappers and two popular mutation callers in isolation.

By preparing libraries and generating sequence from the same MB tumour-normal pair at five different sequencing centres, we obtained results that suggest that PCR-free library preparation protocols should be the method of choice to ensure evenness of coverage, and that a sequencing depth of close to 100 × for both tumour and normal ought to be aimed for (particularly in situations where subclonal mutations or noncoding alterations are suspected to be playing a role). With platforms such as the Illumina HiSeq X now coming online in more centres, such an increase in coverage may be feasible without dramatically increasing costs.

This exercise also afforded a unique opportunity to compare validation schemes for benchmark creation. We found that high-depth (∼300 ×) WGS, in contrast to targeted resequencing, allowed us to more accurately assess FN rates in addition to FP rates, as well as better enable us to determine sweet spots of pipelines.

We have found that, contrary to common perception, identifying somatic mutations, be they SSMs or SIMs, from WGS data is still a major challenge. Calling mutations with different pipelines on differently prepared sequence read sets resulted in a low level of consensus. Using a standard pipeline had the potential of improving on this but still suffered from inadequate controls for library preparation and sequencing artefacts. Using a common high-quality sequence data set yielded higher concordance, but still resulted in substantial discrepancies in somatic mutation call rates and the calls themselves in the hands of different analysis groups. In some cases we were able to identify the Achilles' heels of pipelines for failing to identify or for overcalling mutations as a function of genomic features or properties of sequencing depth, quality or alignment problems. We found that dominating features of both FP and FN SSMs were low coverage in the tumour, and aberrant coverage of tumour and normal. Underlying these artefacts are features such as segmental duplications and centromeric repeats. Use of an appropriate reference sequence (including decoy sequences) and/or good use of blacklists (problematic high-coverage regions or low mappability) can reduce FPs, while only an increase in overall sequencing depth or more sensitive algorithms (MuTect, for example) can address the FN rate. In contrast, we found that the vast majority of curated SIMs fall in simple/tandem repeats, and yet they are often filtered out because of a concern that they may be artefacts. We found little basis for this concern, at least in our data set that came from a no-PCR library. We found that adjustment of filters related to the number of copies of a repeat unit can increase sensitivity for this type of mutation.

Data analysis pipelines are constructed by integrating software from diverse sources. We found that the particular choice of a pipeline software is not as critical as how each piece of software is applied and how the results are filtered. In many instances, the approaches used in the different software and the assumptions made therein are not evident to the user and many parts are black boxes. We found that certain combinations show much higher compatibility than others, for example, with Novoalign alignments as input, MuTect produces an SSM call set with the lowest overall precision, while the SSM call set produced by Strelka on the same alignment file has the highest overall precision. Using combinations of tools for the same process step, assuming that a result shared by two different approaches has a higher likelihood to be correct, results in higher accuracy^32^. Indeed, we found that some of the higher accuracy pipelines utilize consensus of more than one mutation caller. Our controlled experiment intersecting Strelka and MuTect calls bore this out as well.

Recommended checklist for WGS cancer studies:

- PCR-free library preparation
- Tumour coverage >100 ×
- Control coverage close to tumour coverage (±10%)
- Reference genome hs37d5 (with decoy sequences) or GRCh38 (untested)
- Optimize aligner/variant caller combination
- Combine several mutation callers
- Allow mutations in or near repeats (regions of the genome likely to be more prone to mutation)
- Filter by mapping quality, strand bias, positional bias, presence of soft-clipping to minimize mapping artefacts

To account for many unknowns, variant calling pipeline developers often resort to calibrating their pipelines against known results, for example from a genotyping experiment performed on similar samples. This approach might only have limited validity genome-wide, as genotyping assays are typically biased towards less complex areas of the genome. We show that for cancer WGS experiments our benchmark set has the potential to be an unbiased and powerful calibration tool. The sequencing reads and curated Gold Set mutations described here are available to the research community through the ICGC DACO and the EGA to benchmark and calibrate their pipelines.

The issues that we have addressed in this study must be resolved (and we think they can, with the use of our benchmark data set) before WGS for cancer analysis can be wholly adopted for clinical use. However, we also suggest that further benchmarks be established to resolve even more difficult mutational features that tumour samples and genomes can present. These include, but are not limited to, low sample purity (contamination of tumour cells by normal cells and also the normal contaminated by tumour cells or viral components), subclonality, structural rearrangements and karyotype aberrations. Real cancers are complex and this complexity continues to challenge somatic mutation calling pipelines. In summary, this valuable resource can serve as a useful tool for the comparative assessment of sequencing pipelines, and gives important new insights into sequencing and analysis strategies as we move into the next big expansion phase of the high-throughput sequencing era.

## Methods

### Patient material

An Institutional Review Board ethical vote (Medical Faculty of the University of Heidelberg) as well as informed consent were obtained according to ICGC the guidelines (www.icgc.org). A limited amount of the original MB DNA can be made available on request.

### Library preparation and sequencing

The libraries were prepared at the different sequencing centres: the National Center for Genome Analysis (CNAG), Barcelona, Spain; the German Cancer Research Center (DKFZ), Heidelberg, Germany; the RIKEN Institute, Tokyo, Japan; the Ontario Institute for Cancer Research (OICR), Toronto, Canada, and the Wellcome Trust Sanger Institute, Hinxton, UK. Some libraries actually comprise a mixture of different libraries (as per the centre's standard protocols); others comprise one library only. An overview of the composition of the different libraries and differences in the library preparation protocols is given in Table 1 and Supplementary Table 1. All samples were sequenced using Illumina technology and chemistry. The majority of reads are of 2 × 100 bp length and are derived from HiSeq2000 or HiSeq2500 sequencers, however, in one read set (L.A), a low number of 2 × 250-bp MiSeq reads are also included.

### Comparison of SSM calls

Each of the participating centres performed mutation calling using the respective in house pipelines (Supplementary Methods). The raw SSM calls were provided in the form of customized Variant Call Format (VCF) files. To provide a fair comparison, only single base point mutations were considered. A call was considered to be equal when both the position and the exact substitution reported were identical. The calls were then sorted according to the number of centres that made this particular call using a custom Perl script. The resulting file was plotted using a custom R-script (both available on request).

### Merging of the bam files to get the 300 × files

To create the high coverage ∼300 × bam files, the raw fastq files were aligned using bwa 0.6.2-r126-tpx aln -t 12 -q 20, followed by bwa-0.6.2-tpx sample -P -T -t 8 -a 1000 -r. The bam files for each centre/library were merged, and duplicates were marked using Picard tools MarkDuplicates Version 1.61. Finally, all merged per-centre bam files were merged using picard-1.95 MergeSamFiles and the header was adjusted using samtools-0.1.19 reheader. Since only reads from different libraries were merged at this step, duplicates were not marked. The coverage was calculated using an in-house tool, taking into account only non-N bases.

### Downsampling of the 300 × files

The ∼300 × bam files were serially downsampled to different coverage levels (250 ×, 200 ×, 150 ×, 100 ×, 50 ×, 30 ×, 20) using picard-1.95 DownsampleSam, and the coverage was determined after each step.

### Determination of library GC bias

To determine the GC bias of the libraries, we first created 10 kb windows over the whole genome using bedtools (v2.16.2) makewindows. Then, the GC content for each window was calculated using bedtools (v2.16.2) nuc. Windows containing more than 100 ‘N' bases were excluded (awk-3.1.6 ‘BEGIN{FS='\t'}{if ($10 <=100 && $11 <=100) print $1"\t"$2"\t"$3"\t"$5}'). Finally, the coverage for each of the remaining windows was calculated using bedtools (v2.16.2) multicov. Since the total coverage of the different libraries was not the same, the coverage was normalized by dividing the coverage for each window by the mean coverage across all windows for each of the samples. To visualize the GC bias, we then plotted the normalized coverage against the GC content.

### Calculation of low coverage in special regions of interest

The regions of interest were defined as previously described^33^. To determine the percentage of bases covered with fewer than 10 reads, we first determined the coverage over the whole genome in per-base resolution using genomeCoverageBed (v2.16.2) -bga. The resulting coverage file was compressed using bgzip, and an index was produced with tabix-0.2.5 -p bed. We then extracted the coverage for our regions of interest using tabix-0.2.5. From the resulting extracted coverage files, we computed the number of bases covered by a certain number of reads using intersectBed and a custom perl script. This table was then used to determine the percentage of bases covered by ≤10 reads.

### Extraction of mutation signatures

Mutational catalogues were generated based on the somatic mutations detected in the tumours. The 3′ and 5′ sequence context of all selected mutations was extracted, and the resulting trinucleotides were converted to the pyrimidine context of the substituted base. Considering six basic substitution types with surrounding sequence context, this results in a mutation-type vector of length 96. The mutational catalogue was set up by counting the occurrence of each of these 96 mutation types per sample.

The proportions of the signatures published by Alexandrov *et al*.^34^,^35^ contributing to the mutational profile of each sample were estimated based on the probabilities of point mutations with their trinucleotide context in the signatures. The respective exposures were extracted sample-wise by quadratic programming. Exposures were plotted if they accounted for at least 5% of the SSMs in a sample.

### Somatic mutation calling benchmark data set

The sequencing reads provided to pipeline benchmark participants were produced at the CNAG using a no-PCR library preparation procedure that was adapted from the KAPA Library Preparation Kit protocol used together with Illumina TruSeq adaptors and omitting PCR amplification, each for the MB tumour and the corresponding normal DNA sample (L.A). For each sample two libraries were prepared with smaller (roughly 300 bases) and larger (roughly 450 bases) insert size. Sizing was performed using agarose gel separation and excision of corresponding size bands. The two tumour libraries were sequenced to 40.5 × and the two normal libraries to 29.6 × using a combination of Illumina HiSeq2000 (2 × 100 bp) and Illumina MiSeq (2 × 250 bp). MiSeq reads contributed about 2 × to each tumour and normal data. Reads in the FASTQ format were generated using the RTA software provided by Illumina.

### Verification by 300 × coverage

All reads produced by the different sequencing centres on the MB tumour-normal pair (including the CNAG reads described above) were combined and analysed to generate a curated set of results (Gold Set). The combined sequences gave 314 × coverage of the tumour and 272 × in the normal. Six different teams carried out mutation calling using their pipelines (different combinations of aligners, mutation callers and filters). A consensus set was generated accepting all calls made by more than three submitters (a subset of 10% was reviewed manually to confirm the quality of these calls). All calls made by three or fewer submitters were reviewed manually. We generated Integrative Genomics Viewer (IGV) screenshots centred on the mutation positions, juxtaposing the normal and tumour BWA^36^ alignments. The images were made available for visual inspection and reviewed manually and voted/commented on by the entire analysis team (more than eight researchers). For calls that did not achieve complete agreement with the reviewers, a final decision was reached as follows. Reads were aligned with GEM^37^ (gem-mapper) and converted to the BAM format using gemtools scorereads. Alignments were filtered to retain only primary alignments with mapping quality ≥20. Duplicates were removed with Picard, indels realigned at 1,000 genomes indel target locations and all indels were left-aligned using GATK. The pileups at SSM positions were extracted using samtools mpileup with base-quality threshold ≥13. Read depth and base counts were extracted using a custom script. Mutant allele and normal counts were compared using in-house software *snape-cmp-counts*^38^, which compares alternate and reference allele counts in tumour and normal and then assigns a score according to the probability that they are derived from different beta distributions. Mappabilities with 0, 1 and 2% mismatches were computed for the reference genome (h37d5). The average mappabilities in 100-bp windows preceding each candidate mutation were stored as tracks for visualization in IGV. In addition, the segmental duplication annotation from the UCSC browser was loaded into IGV. Mutations were then classified as follows. Mutations with sufficient depth (≥20) and a *snape-cmp-counts* score ≥0.98, average mappability of one and no overlap with segmental duplications were automatically classified in the Gold Set according to their mutant AF (class 1: MAF≥0.1, class 2: 0.1>MAF≥0.05 or class 3: MAF<0.05). All other candidates with *snape-cmp-counts* score >0.9 were reviewed visually in IGV. At this point, extensive soft-clipping in BWA, obvious strand bias and positional bias were also taken into consideration. Mutations with ambiguous alignments were assigned to class 4. Abnormally low or high depth mutations (taking also into account large-scale copy number variant regions) were assigned to class 5. Somatic mutation Gold Set tiers were compiled by cumulative addition of classes so that Tier 1 only includes class 1, while Tier 2 includes class 1 and class 2, Tier 3 includes classes 1, 2 and 3 and so on. All other candidate mutations were rejected and assigned to class 0 (Table 1). The estimated mutation AF cutoff is 2%, below which we deemed a call unreliable. The Gold Set was made available to all participants to review why a somatic mutation was wrongly called or missed in their respective submission.

### Evaluation of submissions

Automatic validation was performed on the submission server to minimize formatting problems. In addition, the submitted VCF files were sorted, filtered to restrict calls to chromosomes 1–22, X and Y and SIMs were left-aligned. Submissions of both CLL and MB SSMs and SIMs were evaluated against their respective Gold Sets, whose derivation is described above. For calculation of recall, the curated mutations were classified into three tiers according to alternate (mutation) AF. Only positions were considered, not the genotypes reported. For calculation of precision, all Tier 3 mutations plus ambiguously aligned mutations (class 4) were included so as to not penalize difficult to align but otherwise convincing differences between tumour and normal samples. For SIMs, no stratification into tiers 2 or 3 was performed; for recall, Tier 1 SIMs were used while, for precision, Tier 4 SIMs were used. To compare overall performance, we used a balanced measure of accuracy, the F1 score, defined as 2 × (Precision × Recall)/(Precision+Recall).

Overlap calculations for the purpose of clustering and heat map generation were performed using Tier 3 for SSMs and Tier 1 for SIMs. The Jaccard index is defined as the intersection divided by the union.

Correspondence analysis was performed using the ‘ca' package in R on a table where each row corresponds to a genomic position at which at least one submission calls a somatic mutation in the Gold Set. The columns comprise the presence or absence in each call set, and Boolean values indicating whether certain genomic features such as repeats or presence in a blacklisted region apply, as well as sequence data such as AF or depth.

Rainfall plots represent the distance for each SSM call from its immediately prior SSM on the reference genome. For each submission, the SSM set used was made of SSM called classified as TP or FP and SSM from the Gold Set Tier 4 that were absent from the submission, classified as FN positions.

Feature analysis was conducted as follows. Tandem repeats were annotated with two programmes. Tandem repeats finder^39^ was run on 201-bp windows around each SSM and SIM calls. Any repeats greater than or equal to six repeat units overlapping or immediately adjacent to the mutation position was annotated accordingly. SGA^25^ was also used to annotate homopolymers specifically, giving a richer annotation of the repeat context and change induced by the mutation. Mappability was calculated using gem mappability^40^ with one mismatch at both 100mer and 150mer lengths. The average 100mer and 150mer mappabilities for each mutation were calculated for a window of −90 to −10 or −140 to −10 with respect to its position, respectively. Mult100 and mult150 are defined as 1—mappability 100 and 150. Same AF is defined as 1−(2 × (SCORE_snape-cmp-counts_−0.5)) for SCORE≥0.5 else 0.

### Control of pipeline components

The protocol consisted of choosing a genome reference, mapping, alignment processing, variant calling and then analysis of alignments or variant calls.

Genome references tested:

- ‘b37d' (‘human_g1k_v37_decoy' from GATK bundle 2.8)
- ‘b37' (‘human_g1k_v37' from GATK bundle 2.8)
- ‘hg19r' (‘ucsc.hg19' from GATK bundle 2.3, with its unplaced contigs and haplotype chromosomes removed)

Mappers tested:

- Novoalign2 (v2.08.03) options: -i PE 360,60 -r All 10
- BWA (0.6.2-r126-tpx)
- BWA-mem (0.7.7-r441) options: -t 8 -M -B 3
- GEM (1.828)

Mutation callers tested:

- MuTect^31^ (v. 1.1.4; dbSNP v. 138; COSMIC v. 64)
- Strelka^27^ (1.0.13)

Alignment post-processing ensuring format compatibility with the downstream tools was performed as follows:

- Merge multiple SAM/BAM output files, coordinate-sorting of alignments with Picard tools (v. 1.84)
- Add read group information with Picard tools
- Discard secondary alignments (alignments with SAM FLAG 0 × 100 set) with samtools (v. 0.1.18) (‘samtools view -h -b -F 256')
- Mark duplicates with Picard tools' MarkDuplicates.
- Realignment (RealignerTargetCreator, IndelRealigner) around indels with GATK (v. 2.3-9-ge5ebf34). The tumour and control were processed together.
- Apply Picard tools' FixMateInformation

No base-quality recalibration or mutation filtration was applied. Two hundred fifty-one-nucleotide-long reads mapped by Novoalign were truncated to 150 nucleotides; this affected ∼4.4% of tumour reads (causing possible FNs because of missing mutation support in the tumour) and ∼4.5% of control reads (causing possible FPs because of missing mutation evidence in the control).

The programme qProfiler (http://sourceforge.net/p/adamajava/wiki/qProfiler/) was run on each mapper's alignment files to investigate systematic mapping differences that potentially influenced subsequent mutation calling. Specifically, distributions of values in SAM fields ‘RNAME' (alignment reference sequence ID), ‘MAPQ' (alignment mapping quality score), ‘TLEN' (observed insert size), ‘CIGAR' (alignment-level indel details together with ‘soft clippings'—mapper-induced read trimming) and ‘MD' (alignment mismatch details) were of interest. The same alignment files were used for mutation calling and qprofiler analysis. However, since mutation calling results were limited to chromosomes 1–22, X and Y, alignment files serving as qprofiler input were first filtered so as to contain only alignments to chromosomes 1–22, X and Y in order for the statistics being relevant (contig coverage statistics being the only exception, since mapping to the decoy contig appears to be important).

### Availability of data

Sequence data for this study have been deposited in the European Genome-phenome Archive (EGA) under the accession number EGAS00001001539.

## Additional information

**How to cite this article:** Alioto, T. S. *et al*. A comprehensive assessment of somatic mutation detection in cancer using whole-genome sequencing. *Nat. Commun.* 6:10001 doi: 10.1038/ncomms10001 (2015).

## Supplementary Material

Supplementary Information Supplementary Figures 1-48, Supplementary Tables 1-18, Supplementary Note 1, Supplementary Methods and Supplementary References

Supplementary Data 1 Gold Set mutations, accuracy of pipelines, pipeline parameter tables, and low coverage regions.

## Figures and Tables

**Table 1:** Summary of medulloblastoma tumour-normal pair library construction and sequencing.

Library	Starting DNA (μg)	Fragment Size (bp)	Size selection	Library protocol	PCR cycles	Sequencing machine	Chemistry (Illumina)	Depth (×) control:tumour
L.A	4	∼400	2% Agarose gel	KapaBio	0	HiSeq 2500 HiSeq 2000MiSeq	V1 (RR)V3V2	29.6 : 40.5
L.B	1	∼400	2% Agarose gel, Invitrogen E-gel	TrueSeq DNA	10	HiSeq 2000	V3	44.9 : 62.8
L.C	2.5	∼500	2% Agarose gel	NEBNext	12	HiSeq 2500HiSeq 2000	V1 (RR)V3	58.9 : 66.8
L.D	1	∼550	Agarose gel	TrueSeq DNA	10	HiSeq 2000	V3	35.3 : 39.1
L.E	2.8	∼620	1.5% Agarose gel pippin	NEBNext	0	HiSeq 2000	V3	40.5 : 60.4
L.F	1	∼400	AMPureXP beads	NEBDNA	10	HiSeq 2000	V3	38.7 : 37.9
L.G	1	∼350	AMPureXP beads	TrueSeq DNA PCR-Free	0	HiSeq 2000	V3	19.4 : 19.3
L.H	0.5	∼175	AMPureXP beads	SureSelect WGS	10	HiSeq 2500	V3	28.7 : 26.5

**Table 2:** Classification of SSM and SIM Gold Set mutations for the medulloblastoma benchmark.

	Definition	MB SSM	MB SIM
Class 1	Mutant AF≥0.10	962	337
Class 2	0.05≤Mutant AF<0.10	139	
Class 3	Mutant AF<0.05	154	
Class 4	Ambiguous alignment	8	10
Class 5	High or low depth	29	
Tier 1	Class 1	962	337
Tier 2	Classes 1 and 2	1,101	
Tier 3	Classes 1, 2 and 3	1,255	
Tier 4	Classes 1, 2, 3 and 4	1,263	347
Tier 5	Classes 1, 2, 3, 4 and 5	1,292	

AF, allele frequency; MB, medulloblastoma; SIM, somatic insertion/deletion mutations; SNP, single-nucleotide polymorphisms; SNV, single-nucleotide variant; SSM, somatic single-base mutation.

Numbers of curated mutations falling in each class or tier are shown. Successive tiers represent cumulative addition of lower AF mutations, followed by those supported by ambiguous alignments, and finally those with either too low or too high a depth. SIMs were not subjected to such fine classification, with calls only assigned to classes 1 and 4. Note that we use the terms SSM and SIM for somatic mutations instead of more commonly used terms that ought to be reserved for germline variants such as SNP (refers to a single base variable position in the germline with a frequency of >1% in the general population) or SNV (refers to any single base variable position in the germline including those with a frequency <1% in the general population).

**Table 3:** Summary of accuracy measures.

SSM calls	Aligner	SSM Detection Software	TP	FP	FN	P	R	F1
MB.GOLD	BWA, GEM	Curated	1,255 (8)	0	0	1.00	1.00	1.00
MB.A	BWA	In-house	775 (0)	147	480	0.84	0.62	0.71
MB.B	BWA	samtools, Varscan	788 (1)	12	467	0.99	0.63	0.77
MB.C	GEM	samtools, bcftools	766 (3)	1,025	489	0.43	0.61	0.50
MB.D	n.a.	SMuFin	737 (4)	1,086	518	0.41	0.59	0.48
MB.E	BWA	SomaticSniper	750 (4)	229	505	0.77	0.60	0.67
MB.F	BWA	Strelka	884 (2)	165	371	0.84	0.70	0.77
MB.G	BWA	Caveman, Picnic	899 (3)	140	356	0.87	0.72	0.78
MB.H	Novoalign	MuTect	947 (3)	6,296	308	0.13	**0.76**	0.22
MB.I	BWA	samtools	879 (7)	129	376	0.87	0.70	0.78
MB.J	None, BWA	SGA+freebayes	856 (1)	62	399	0.93	0.68	**0.79**
MB.K	BWA	Atlas2-snp	945 (8)	7,923	310	0.11	0.75	0.19
MB.L1	BWA	MuTect, Strelka	385 (0)	3	870	**0.99**	0.31	0.47
MB.L2	BWA	MuTect, Strelka	900 (1)	253	355	0.78	0.72	0.75
MB.M	BWA mem	samtools, GATK+MuTect	937 (4)	1,695	318	0.36	0.75	0.48
MB.N	BWA	Strelka	847 (1)	289	408	0.75	0.68	0.71
MB.O	BWA	MuTect	944 (3)	272	311	0.78	0.75	0.76
MB.P	BWA	Sidron	833 (3)	256	422	0.77	0.66	0.71
MB.Q	BWA	qSNP+GATK	842 (2)	25	413	0.97	0.67	**0.79**
								
**SIM calls**								
MB.GOLD	BWA, GEM	Curated	337 (10)	0	0	1.00	1.00	1.00
MB.A	BWA	In-house	16 (0)	63	321	0.20	0.05	0.08
MB.B	BWA	GATK SomaticIndelDetector, Varscan	167 (0)	20	173	0.89	0.49	0.63
MB.C	GEM	samtools, bcftools	103 (0)	26	236	0.80	0.30	0.44
MB.D	none	SMuFin	29 (0)	25	308	0.54	0.09	0.15
MB.F	BWA	Strelka	147 (8)	12	193	0.93	0.43	0.58
MB.G	BWA	Pindel	189 (2)	82	152	0.70	0.55	0.61
MB.H	Novoalign	VarScan2	55 (0)	248	282	0.18	0.16	0.17
MB.I	BWA	Platypus	271 (7)	224	70	0.55	**0.79**	**0.65**
MB.J	None	SGA	90 (1)	34	249	0.72	0.26	0.38
MB.K	BWA	Atlas2-indel	268 (6)	444	72	0.38	0.79	0.51
MB.L1	BWA	Strelka	64 (1)	3	273	**0.96**	0.19	0.32
MB.L2	BWA	Strelka	130 (3)	13	210	0.91	0.38	0.53
MB.N	BWA	Strelka	128 (6)	16	209	0.89	0.38	0.53
MB.O	BWA	GATK SomaticIndelDetector	140 (1)	47	197	0.75	0.42	0.53
MB.P	BWA	bcftools, PolyFilter	37 (0)	57	301	0.39	0.11	0.17
MB.Q	BWA	Pindel	100 (2)	61	237	0.63	0.30	0.40

F1, F1 score; FN, false negative; FP, false positives; P, precision; R, recall; TP, true positives.

Shown are the evaluation results with respect to the medulloblastoma Gold Set (Tier 3). Shown are the number of true calls (TP) with additional Tier 4 calls in parentheses, the number of FP, the number of FN, P, R and F1. The submissions with the best precision, recall and F1 score are in bold.
