# Supplementary material for: A comprehensive assessment of somatic mutation detection in cancer using whole-genome sequencing
Source: Nat Commun. 2015 Dec 9;6:10001. doi: 10.1038/ncomms10001 (PMC4682041; doi:10.1038/ncomms10001)
Supplement: Supplementary Information — Supplementary Figures 1-48, Supplementary Tables 1-18, Supplementary Note 1, Supplementary Methods and Supplementary References [file ncomms10001-s1.pdf]

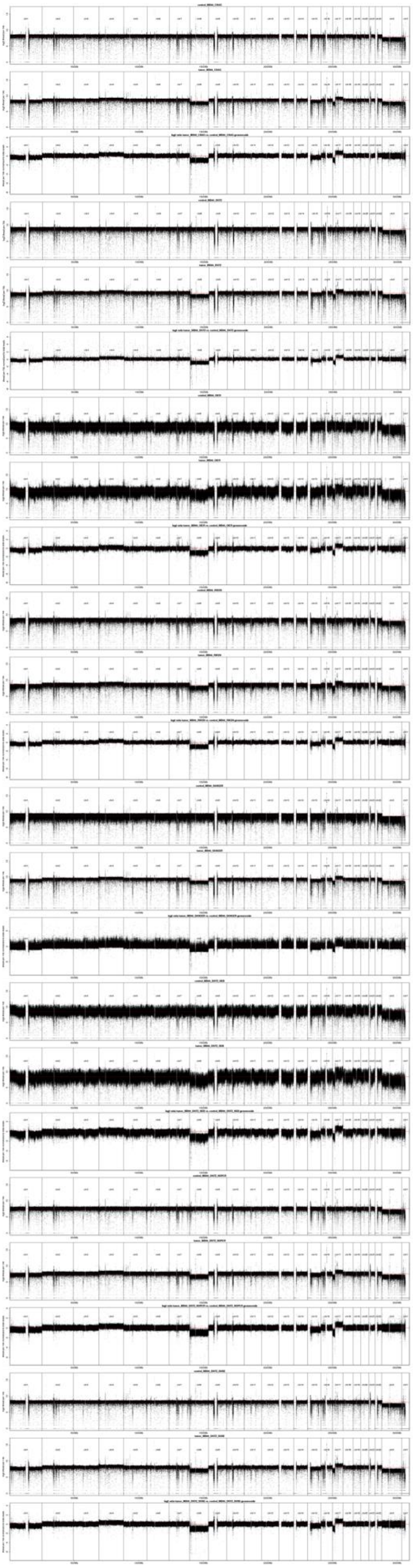

Supplementary Figure 1. Coverage plots for all samples and log2 ratio plots for each submission

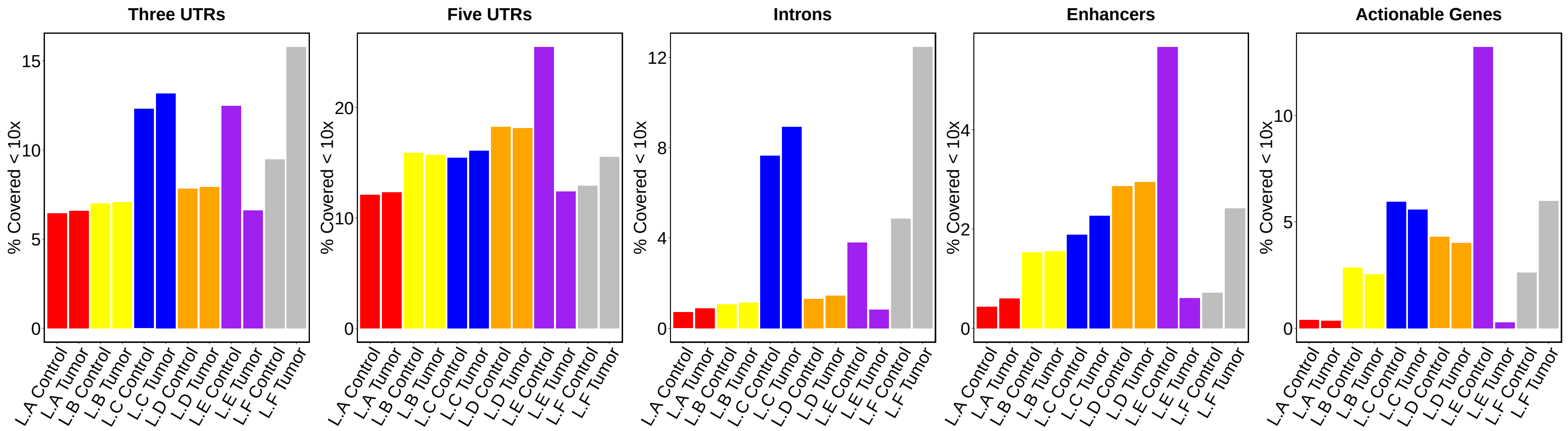

Supplementary Figure 2. Percentage of different regions of interest covered with ≤ 10x (all samples downsampled to 30x).

a

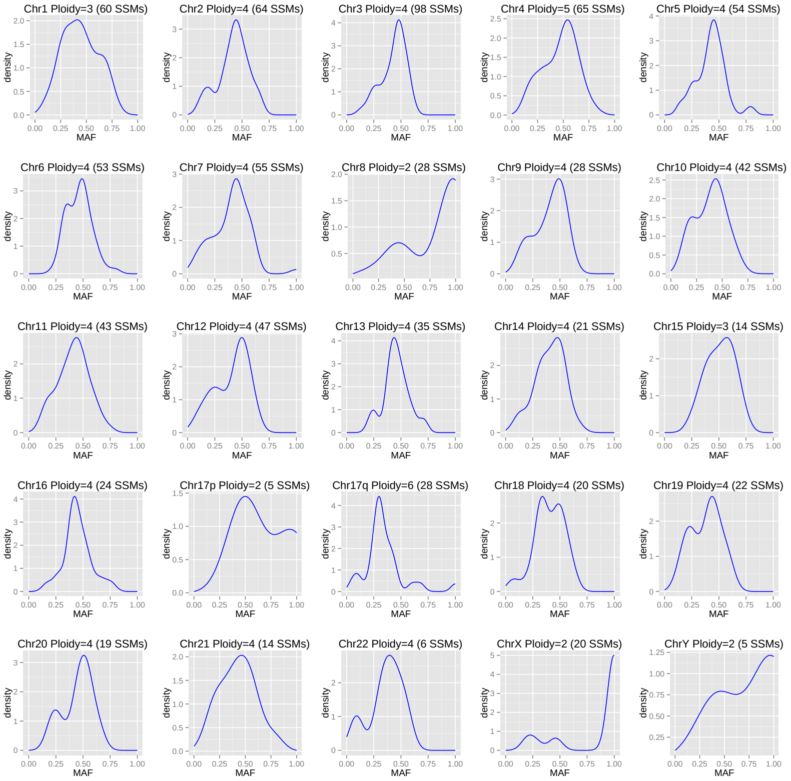

b

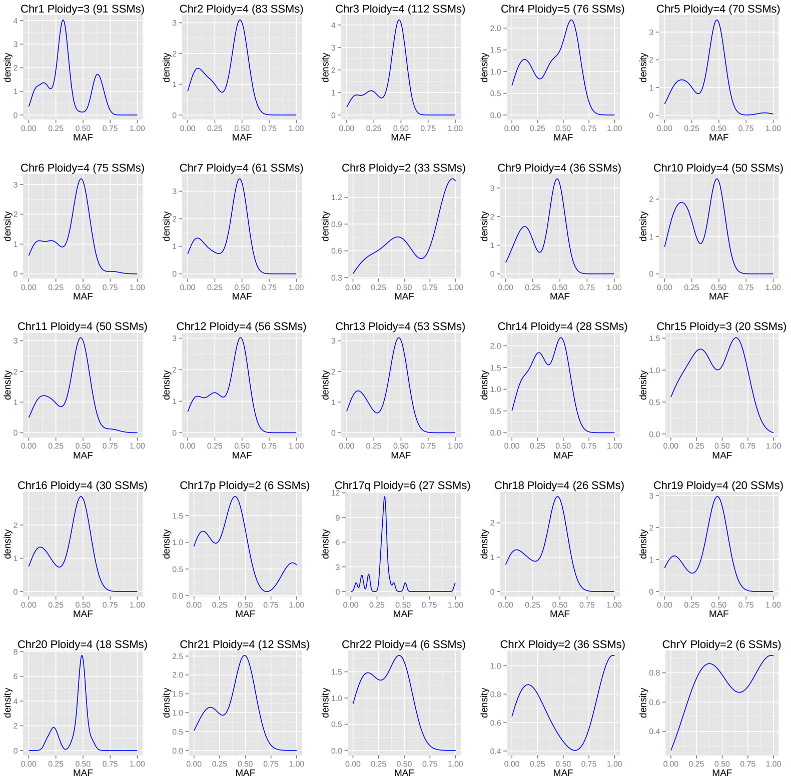

Supplementary Figure 3. Per chromosome variant allele frequency histograms for a) 30x tumor vs. 30x control and b) 250x tumor vs. 250x control.

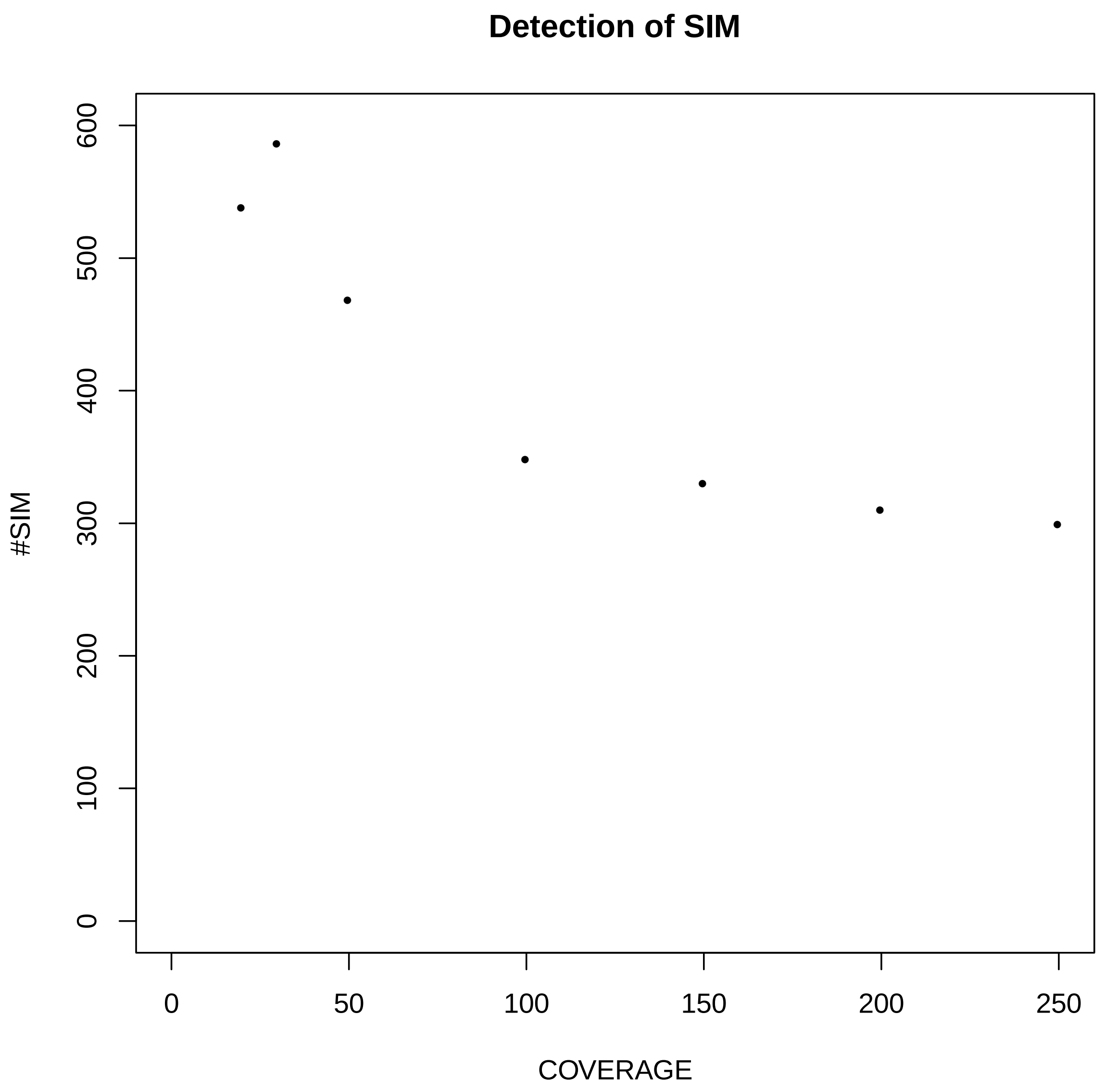

Supplementary Figure 4. Number of SIMs (indels) found dependent on different coverage levels (same coverage for tumor and control).

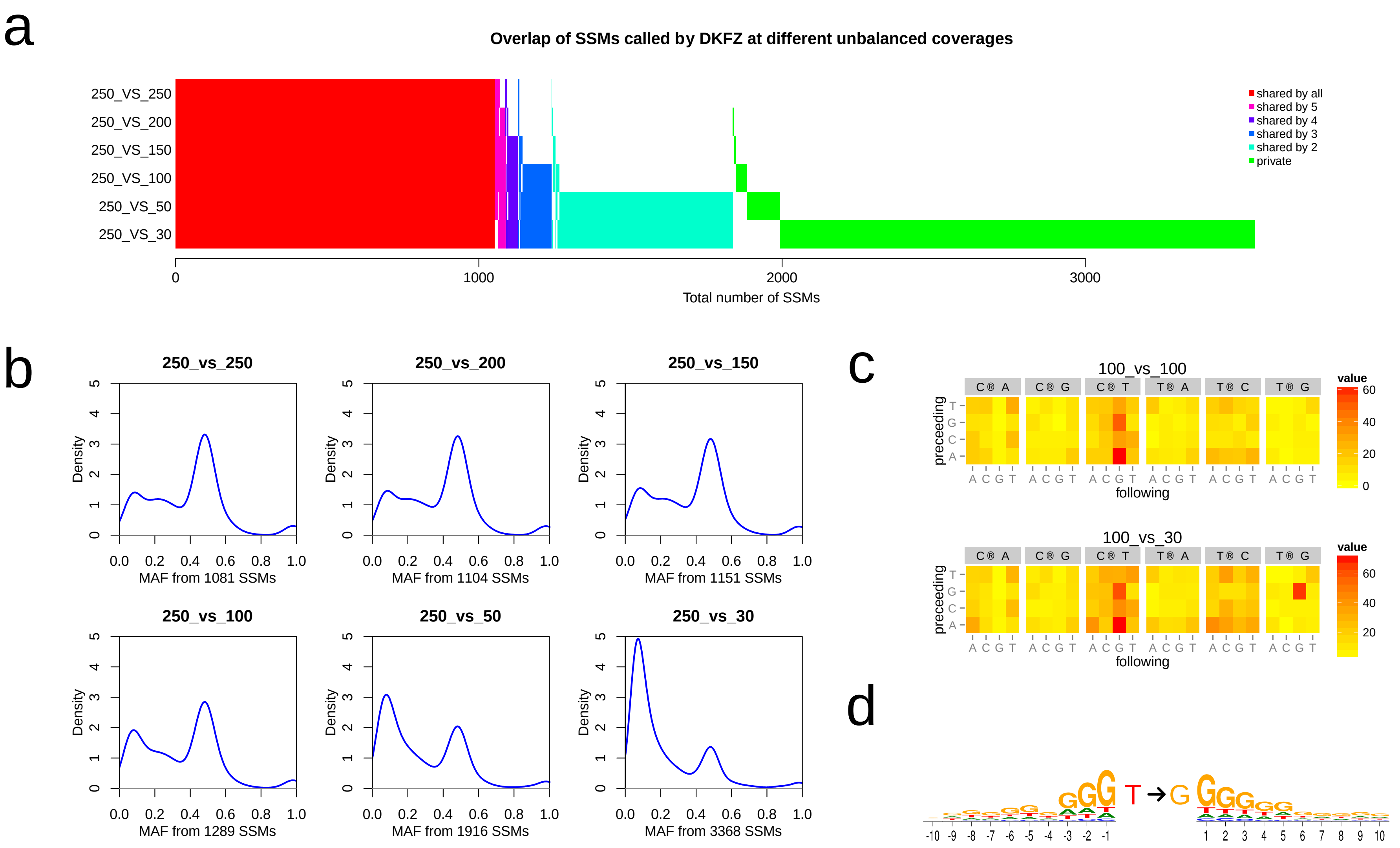

Supplementary Figure 5. Effect of unbalanced coverage between tumor and control on SSM calling. **a**) Overlap of SSMs called on different unbalanced coverages. **b**) Density plots of the variant allele frequencies for different control coverages and a fixed tumor coverage and number of SSMs called in total (calls were done using the DKFZ calling pipeline). **c**) Sequence context of SSM calls derived for two different coverage combinations (100x tumor vs. 100x control and 100x tumor vs. 30x control). **d**) Logo plots showing the window of ten bases upstream and downstream from the presumed T to G transversion artifact.

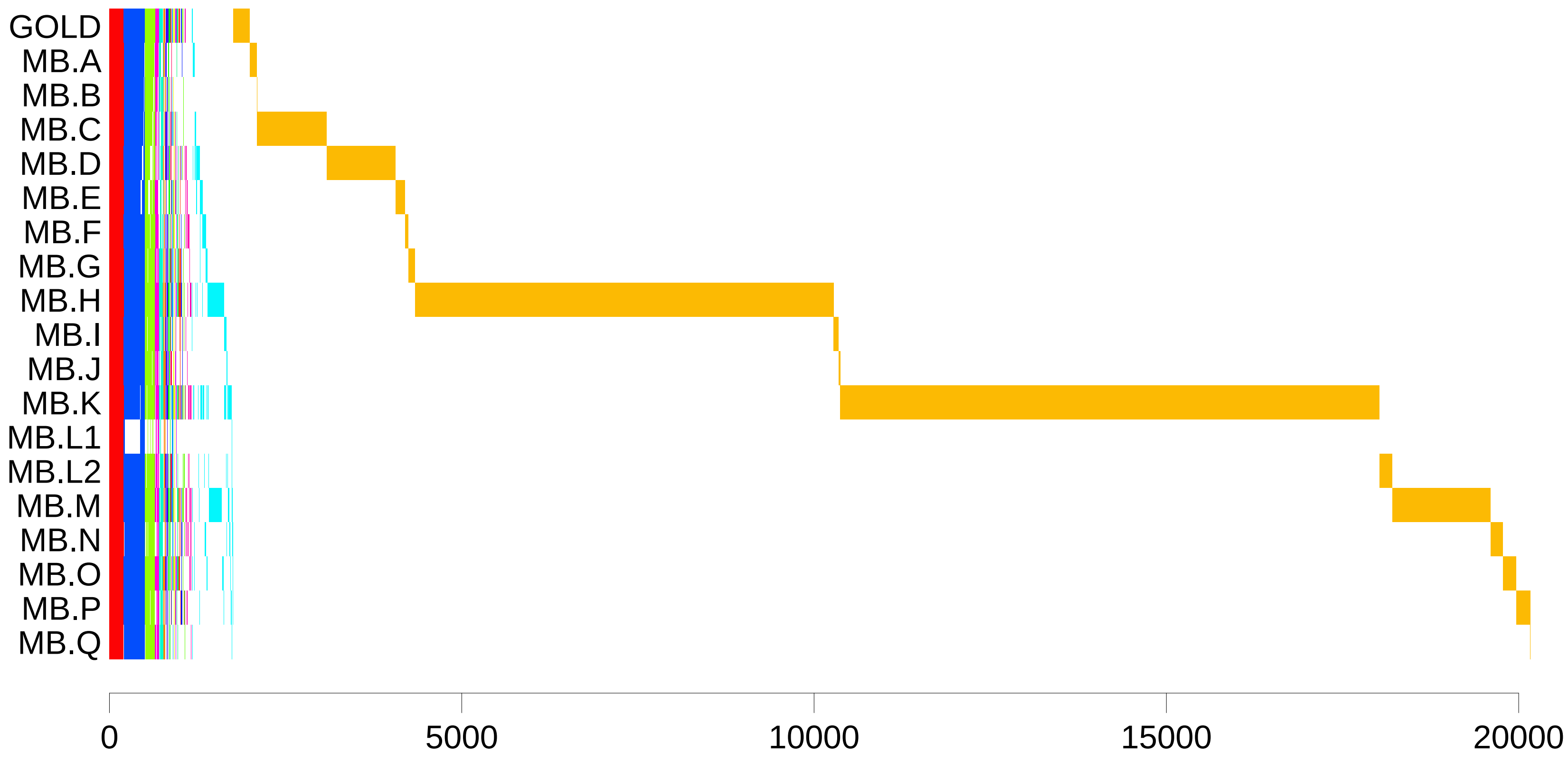

Supplementary Figure 6. Overlap of medulloblastoma SSM calls for each level of concordance. Shared sets of calls are vertically aligned. GOLD indicates the Gold Set.

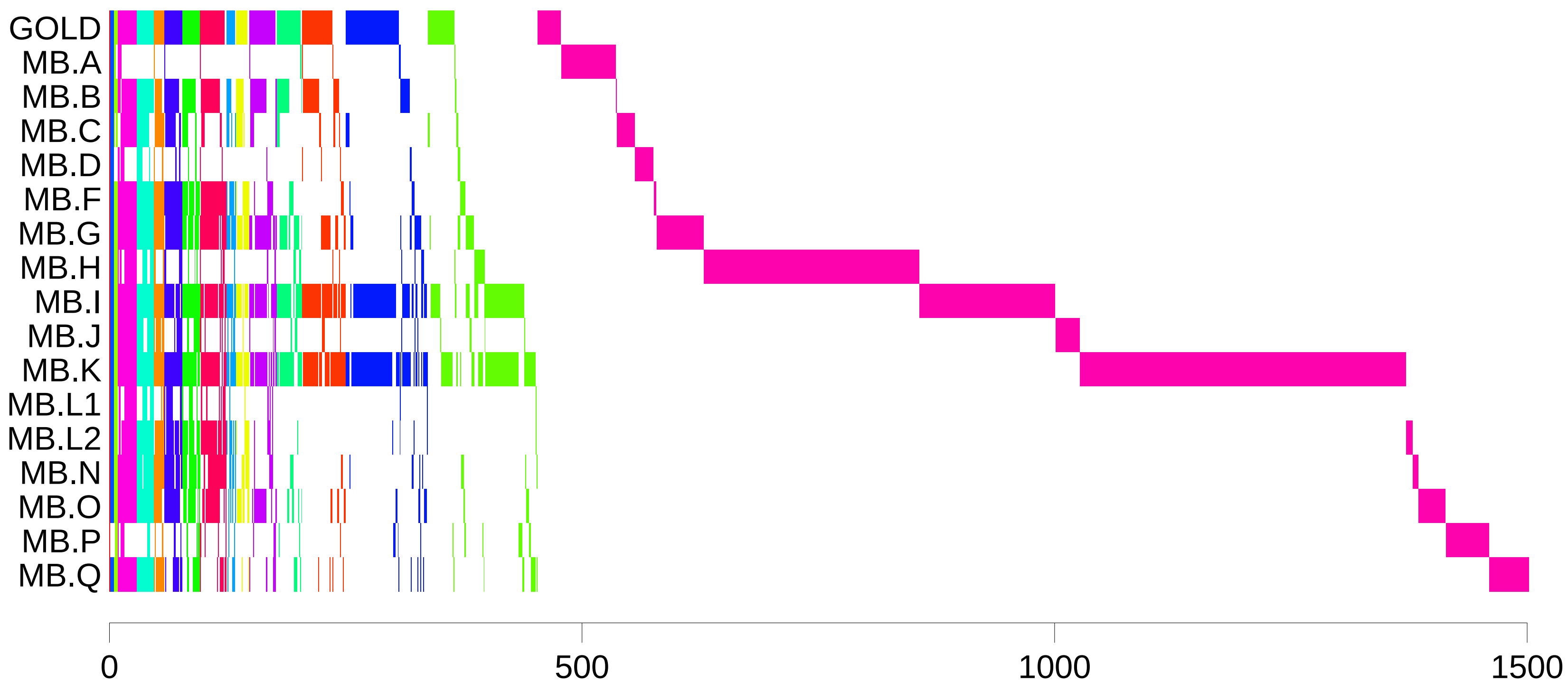

Supplementary Figure 7. Overlap of medulloblastoma SIM calls for each level of concordance. Shared sets of calls are vertically aligned. GOLD indicates the Gold Set.

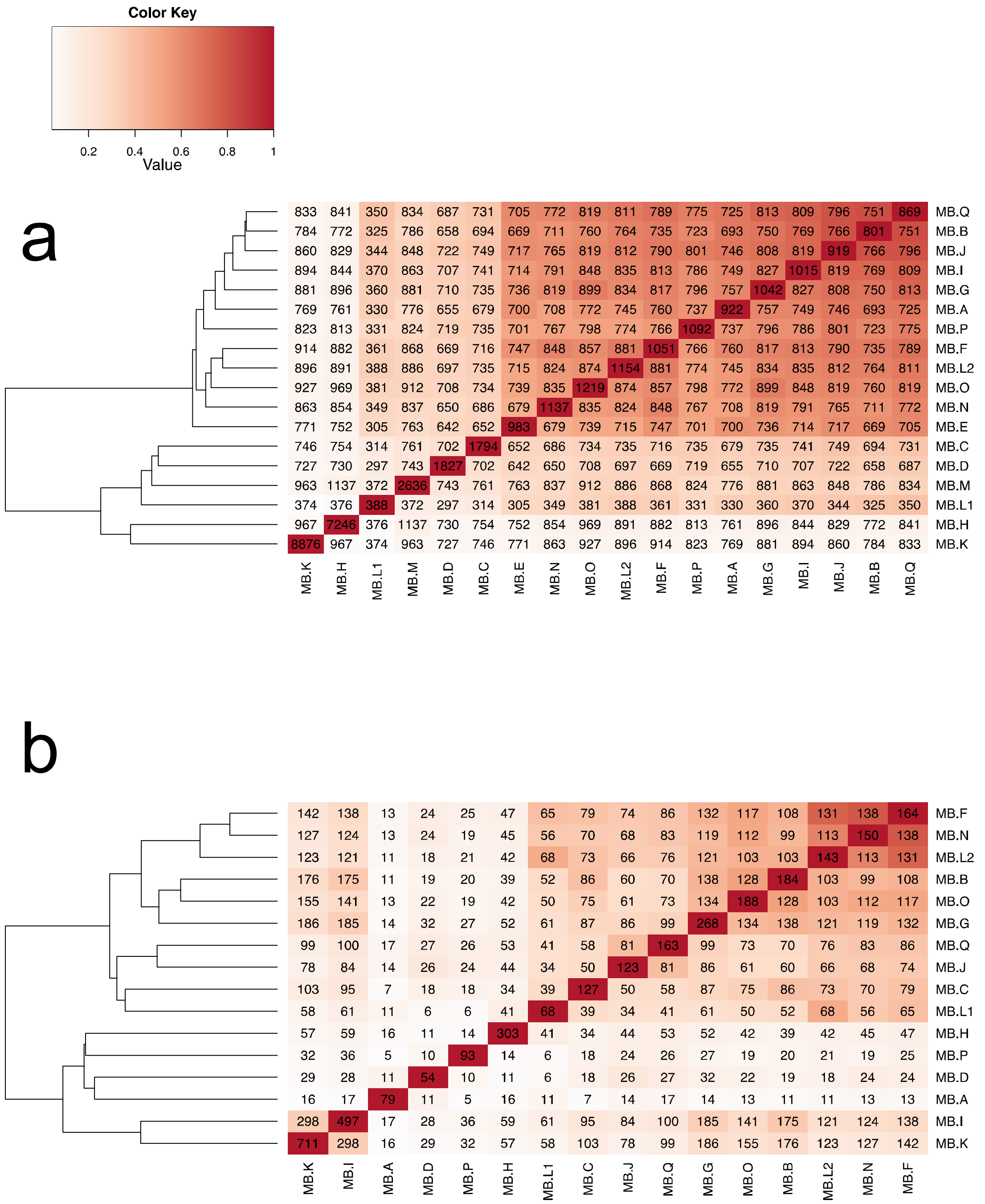

Supplementary Figure 8. Overlap of calls among medulloblastoma mutation call sets. Heat maps and dendrograms reflect the value of the Jaccard index (pairwise ratio of intersection/union). Values in each cell indicate only the number of somatic mutations shared by each pair of submissions. a) Medulloblastoma SSMs. b) Medulloblastoma SIMs.

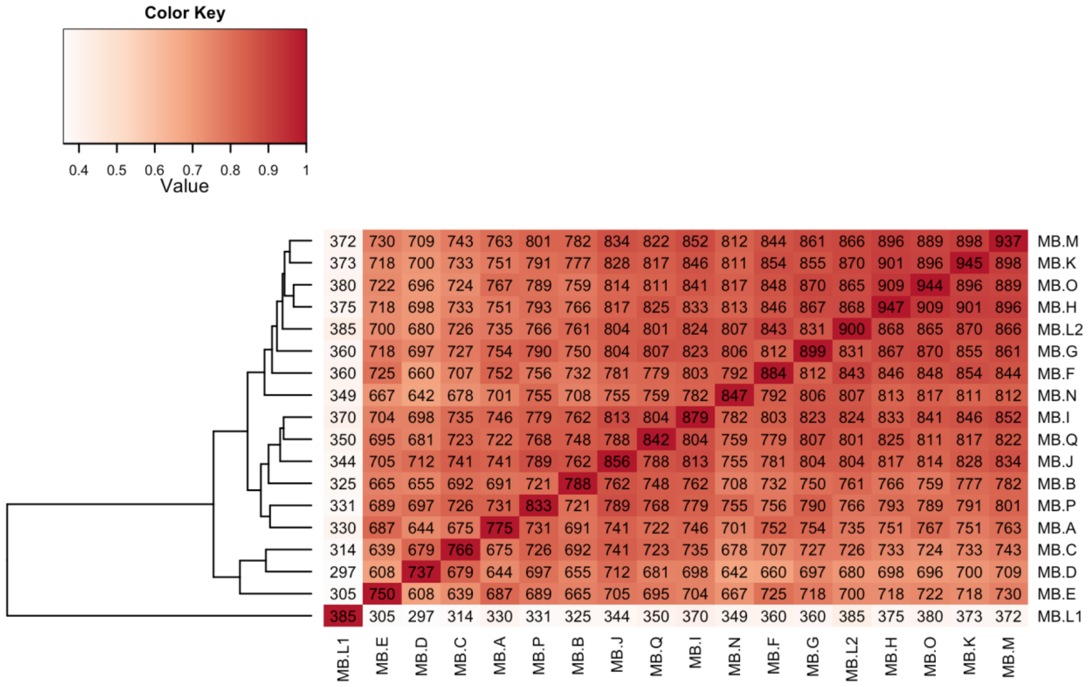

Supplementary Figure 9. Clustering of medulloblastoma SSM true positives.

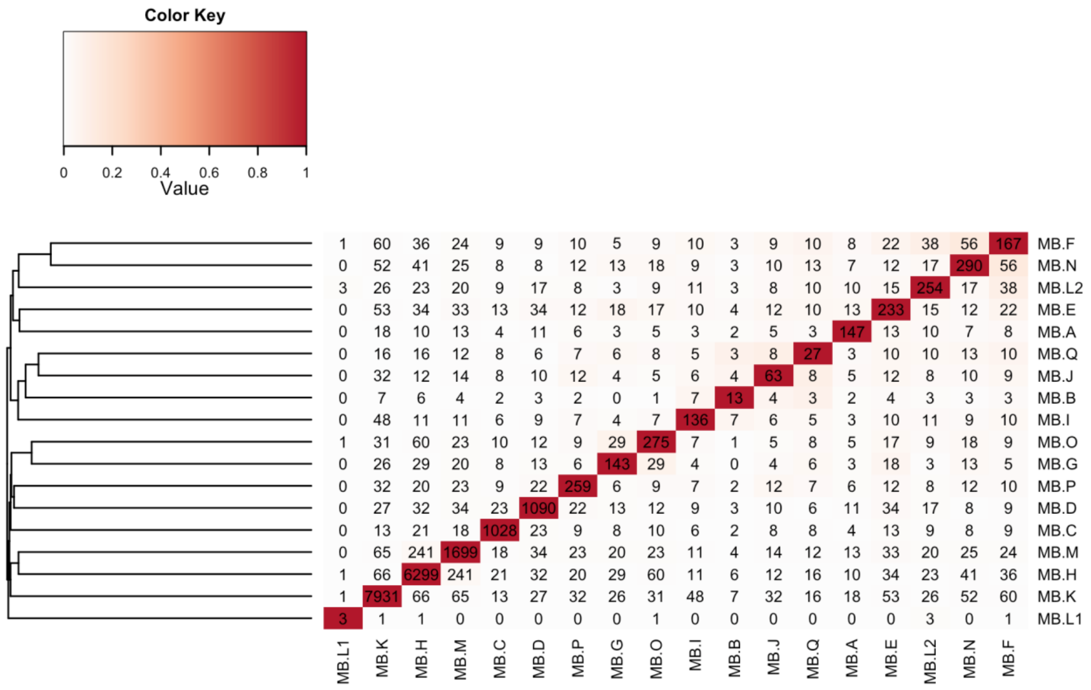

Supplementary Figure 10. Clustering of medulloblastoma SSM false positives.

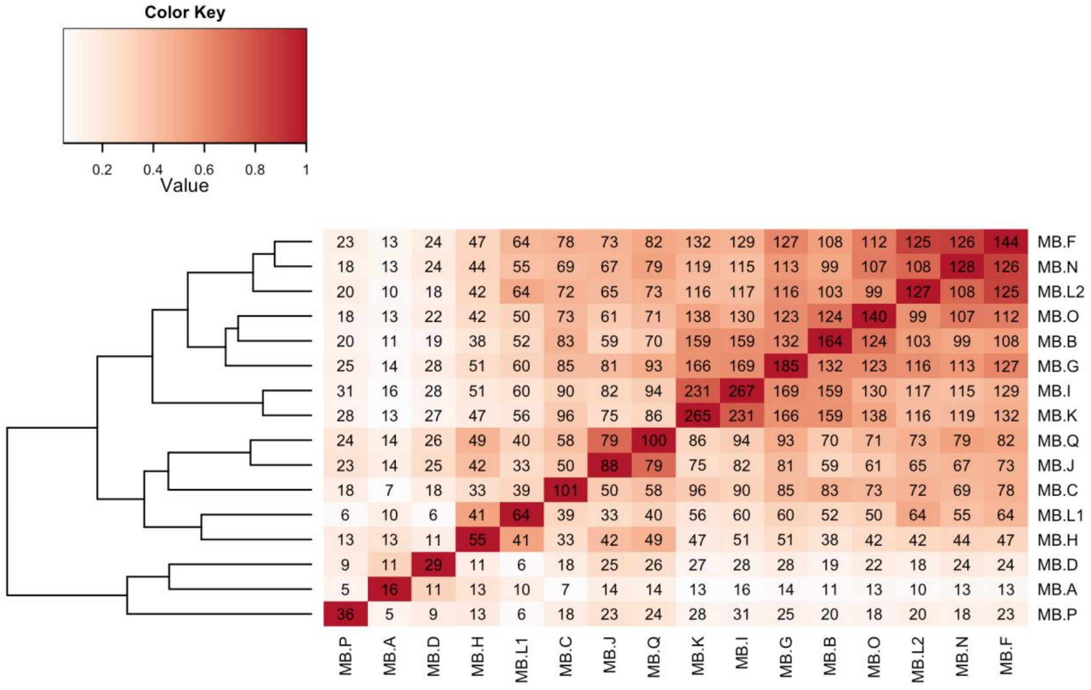

Supplementary Figure 11. Clustering of medulloblastoma SIM true positives.

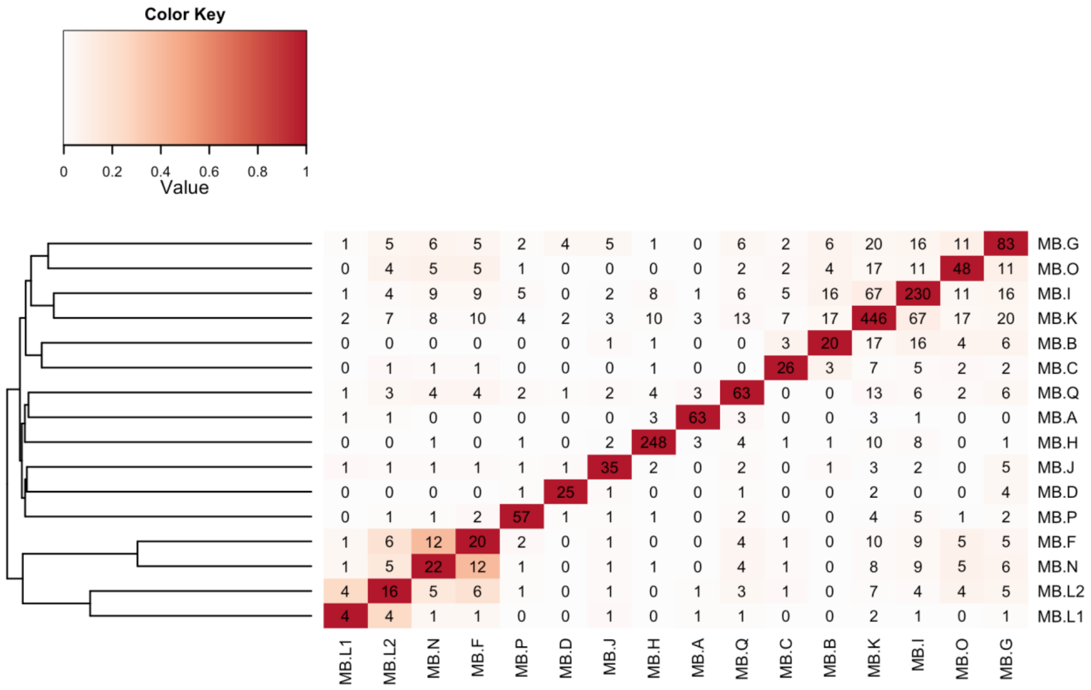

Supplementary Figure 12. Clustering of medulloblastoma SIM false positives.

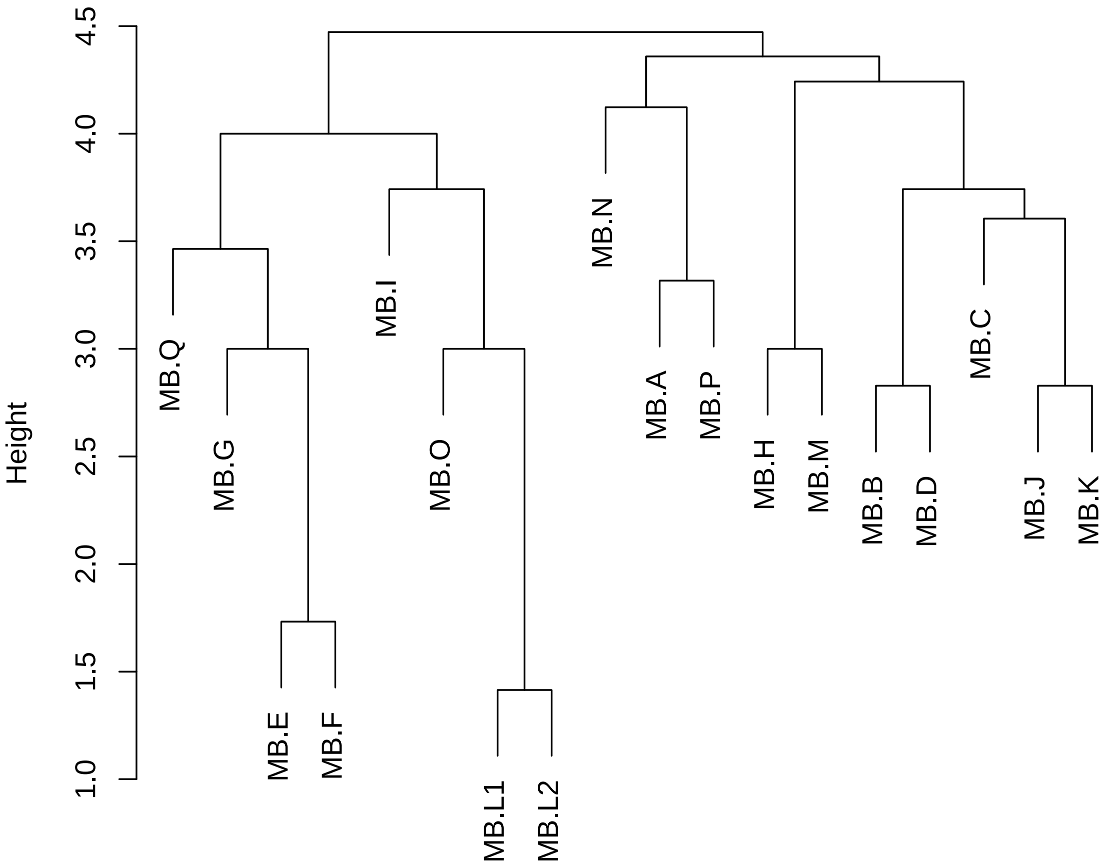

Supplementary Figure 13. Hierarchical clustering of pipeline settings for medulloblastoma submissions.

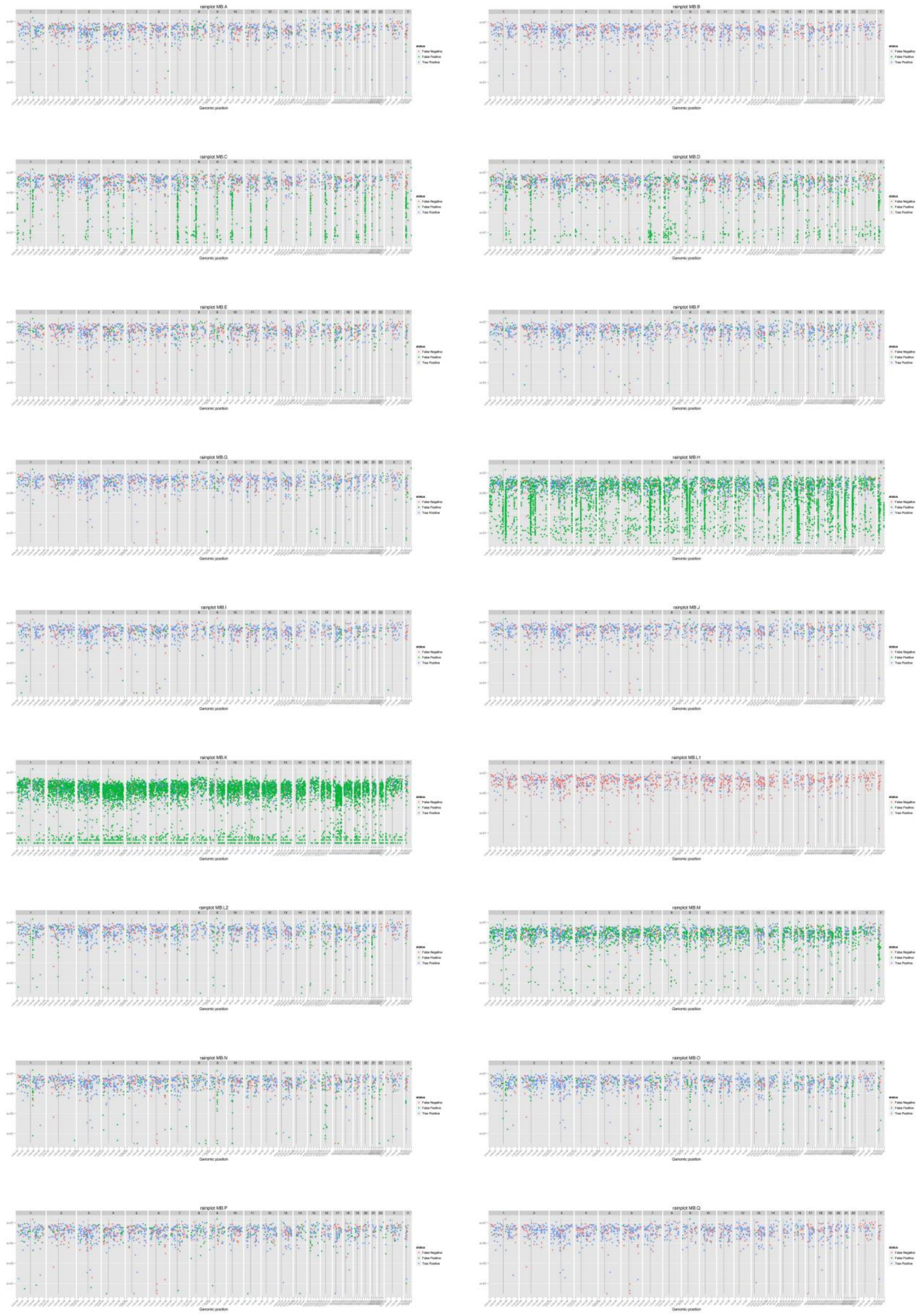

Supplementary Figure 14. Rainfall plots of medulloblastoma submissions. False negatives in red, false positives in green, true positives in blue.

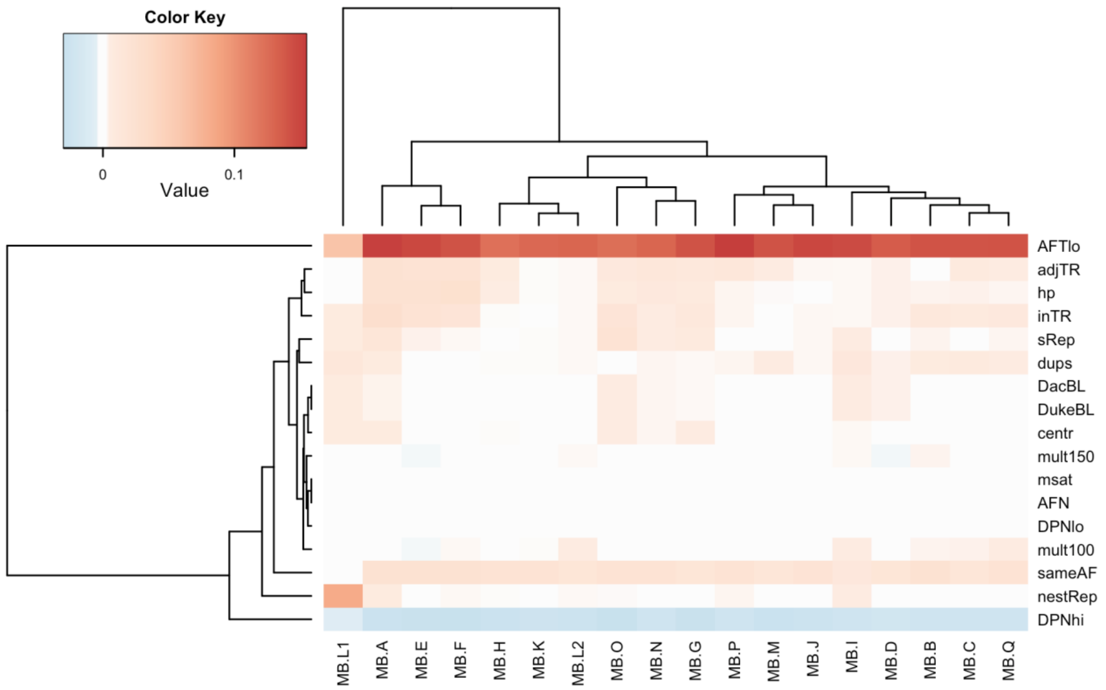

Supplementary Figure 15. Enrichment or depletion of genomic and alignment features in false negative calls for each medulloblastoma SSM submission.

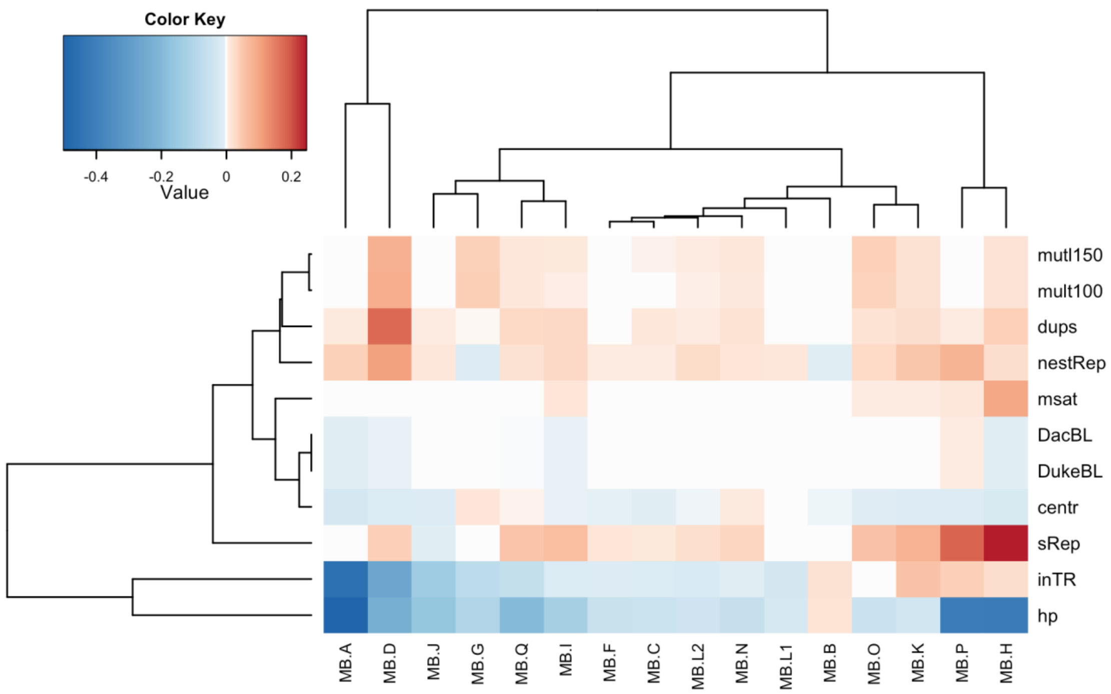

Supplementary Figure 16. Enrichment or depletion of genomic and alignment features in false positive calls for each medulloblastoma SIM submission. Allele frequency and depth were not computed for SIM submissions. Only repeat features were used in this analysis.

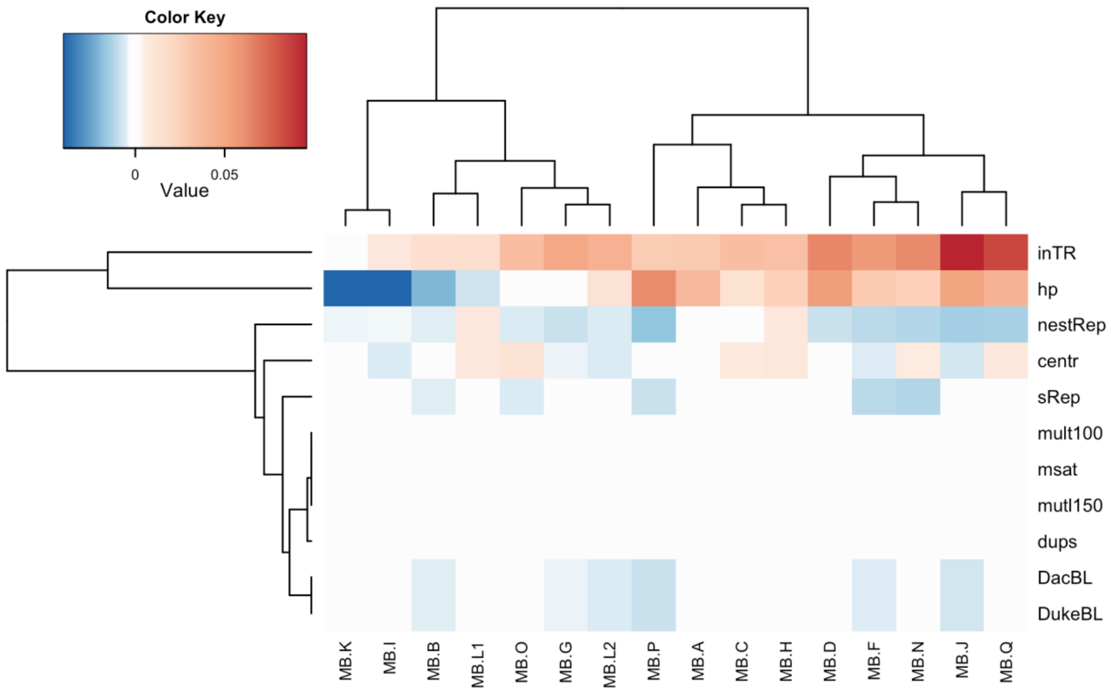

Supplementary Figure 17. Enrichment or depletion of genomic and alignment features in false negative calls for each medulloblastoma SIM submission.

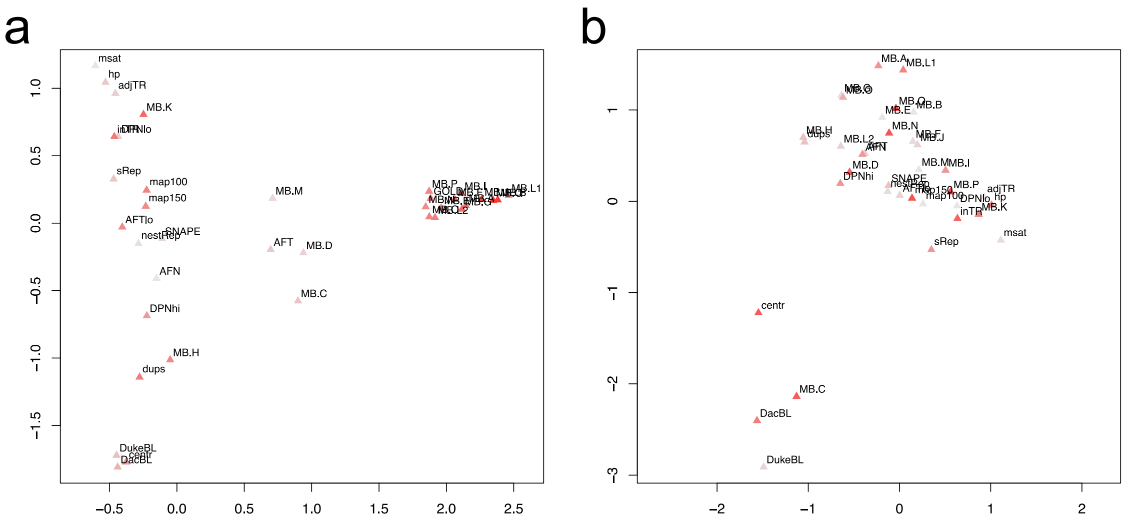

Supplementary Figure 18. Correspondence analysis of submissions for all medulloblastoma SSMs (a) and false-positive-only medulloblastoma SSMs (b) with genomic and alignment features.

a

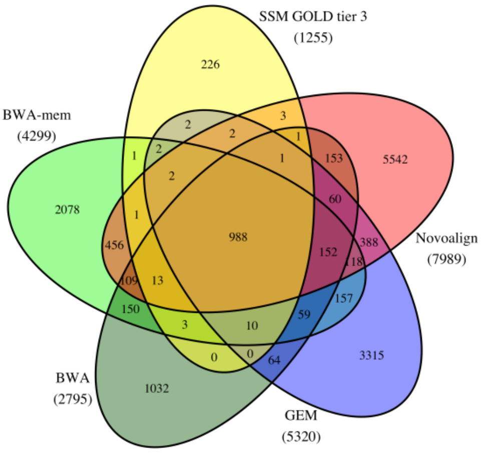

b

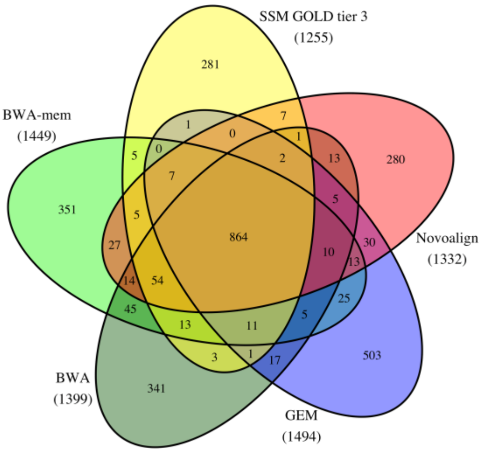

c

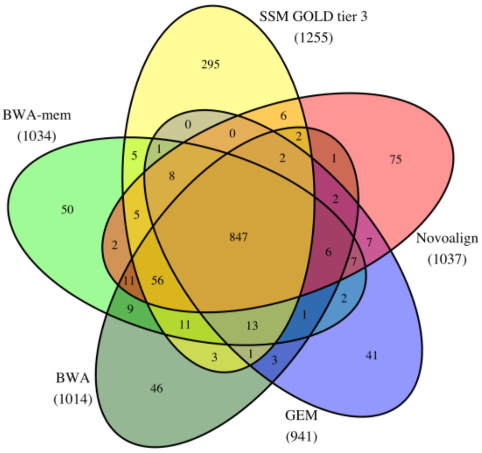

Supplementary Figure 19. Sizes (in parentheses) and overlaps of SSM call sets produced by MuTect (a), Strelka (b) and MuTect+Strelka consensus (c). SSM calling was performed for each mapper-caller combination and the tier 3 SSM Gold Set was used for validation.

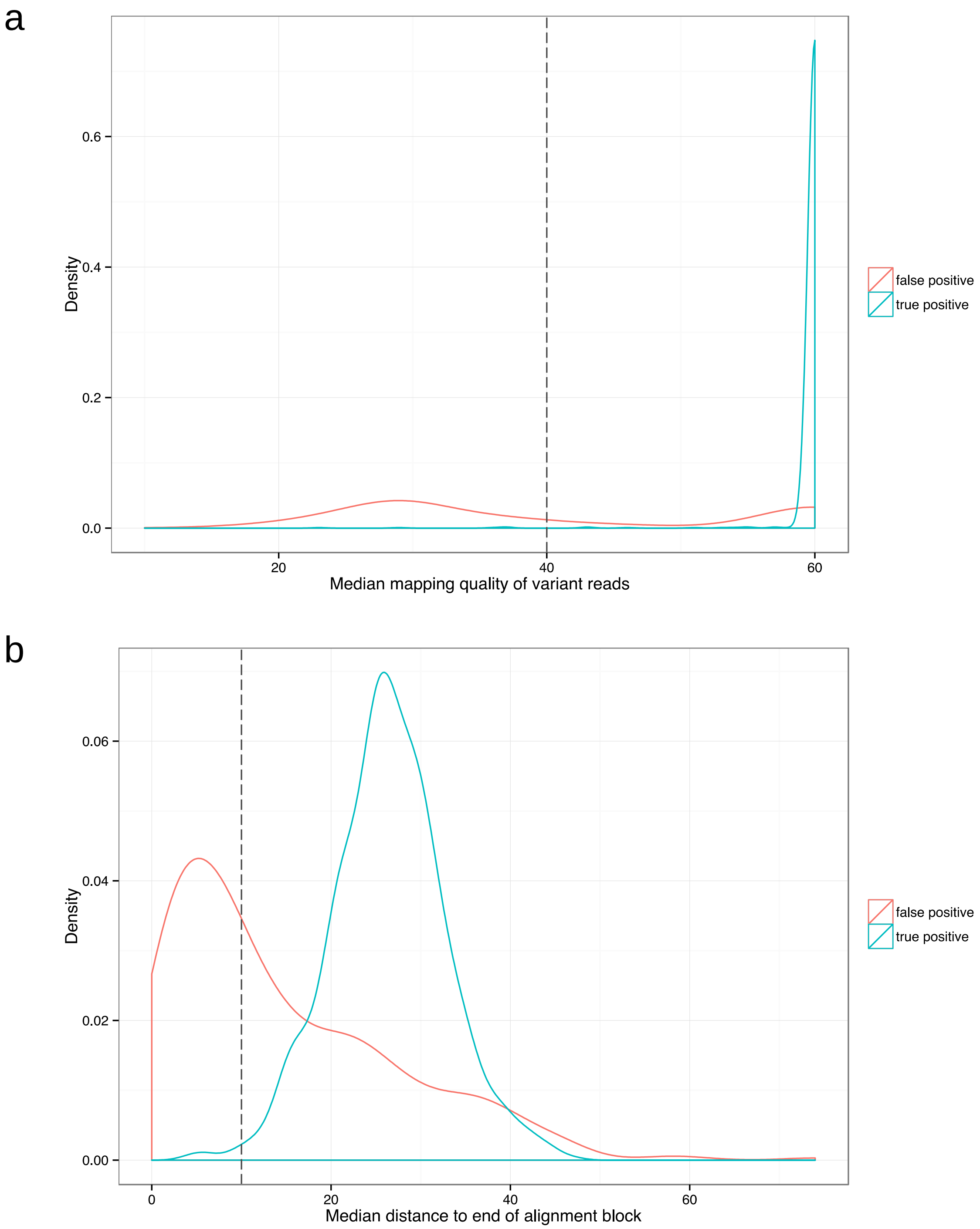

Supplementary Figure 20. (a) Kernel density plot comparing the distribution of true and false positive SSM calls made by Strelka as a function of the median BWA mapping quality for variant reads. True positive calls have higher median mapping qualities, while calls supported by reads with mapping qualities less than 40 are predominantly false negatives (238/243, 98%). Adding the VariantMapQualMedian < 40 filter (supplementary table 9) improves the precision of Strelka calls (those that pass Strelka's built-in filters) from 0.71 to 0.86 with only a slight drop in recall from 0.758 to 0.754. (b) Kernel density plot comparing the distribution of true and false positive Strelka calls as a function of the median distance of the variant position within supporting reads to the end of the alignment block. The DistanceToAlignmentEndMedian < 10 and DistanceToAlignmentEndMAD < 3 filters remove likely false positives caused by misalignments resulting in alternate alleles being clustered at a consistent distance from the start or end of read alignments.

a

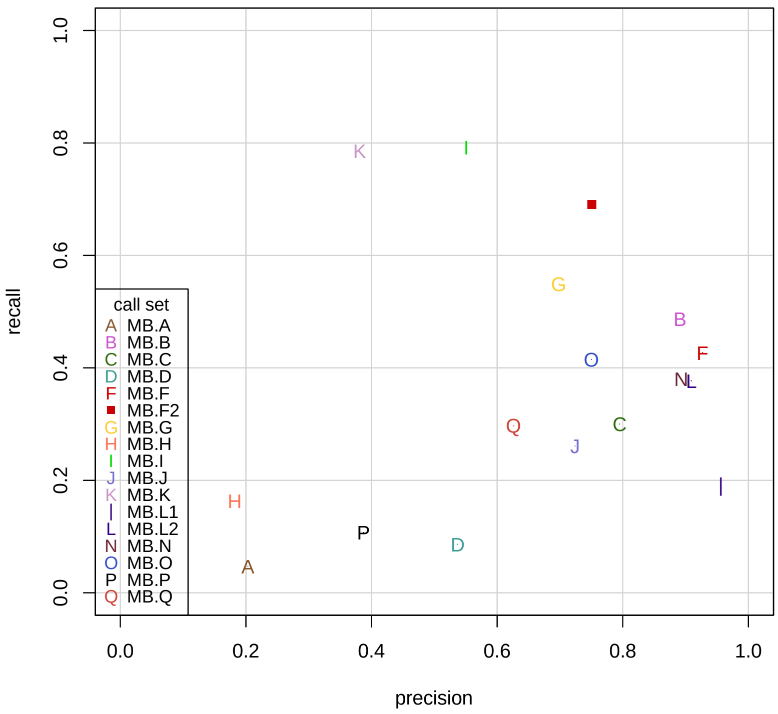

b

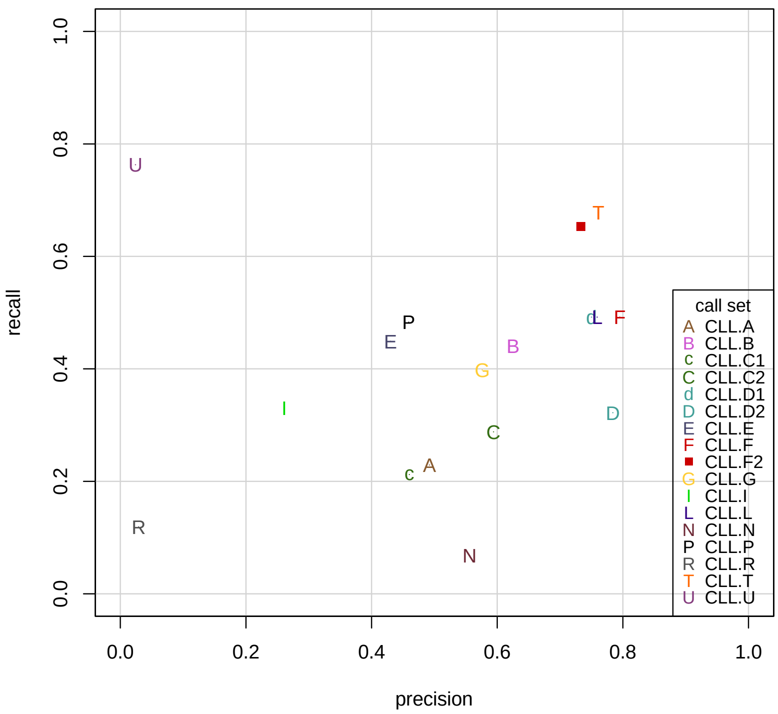

Supplementary Figure 21. By merely turning off the Strelka “repeat copy > 8” filter, we could increase the accuracy of the MB.F and CLL.F pipeline (red squares). Shown are the precision-recall plots for (a) medulloblastoma SIMS and (b) CLL SIMS.

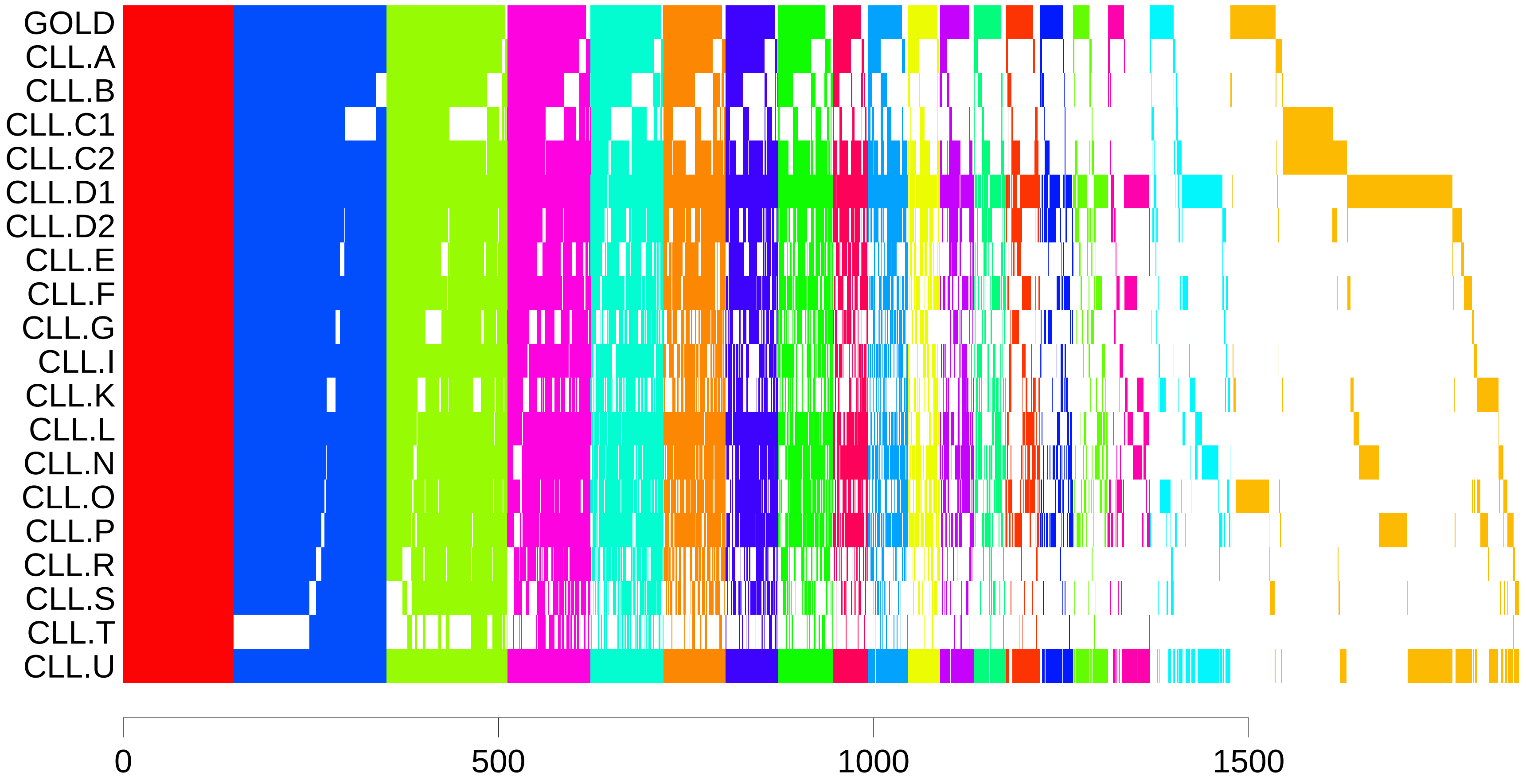

Supplementary Figure 22. Overlap of CLL SSM calls shared by at least two call sets for each level of concordance. Shared sets of calls are vertically aligned. GOLD indicates the Gold Set.

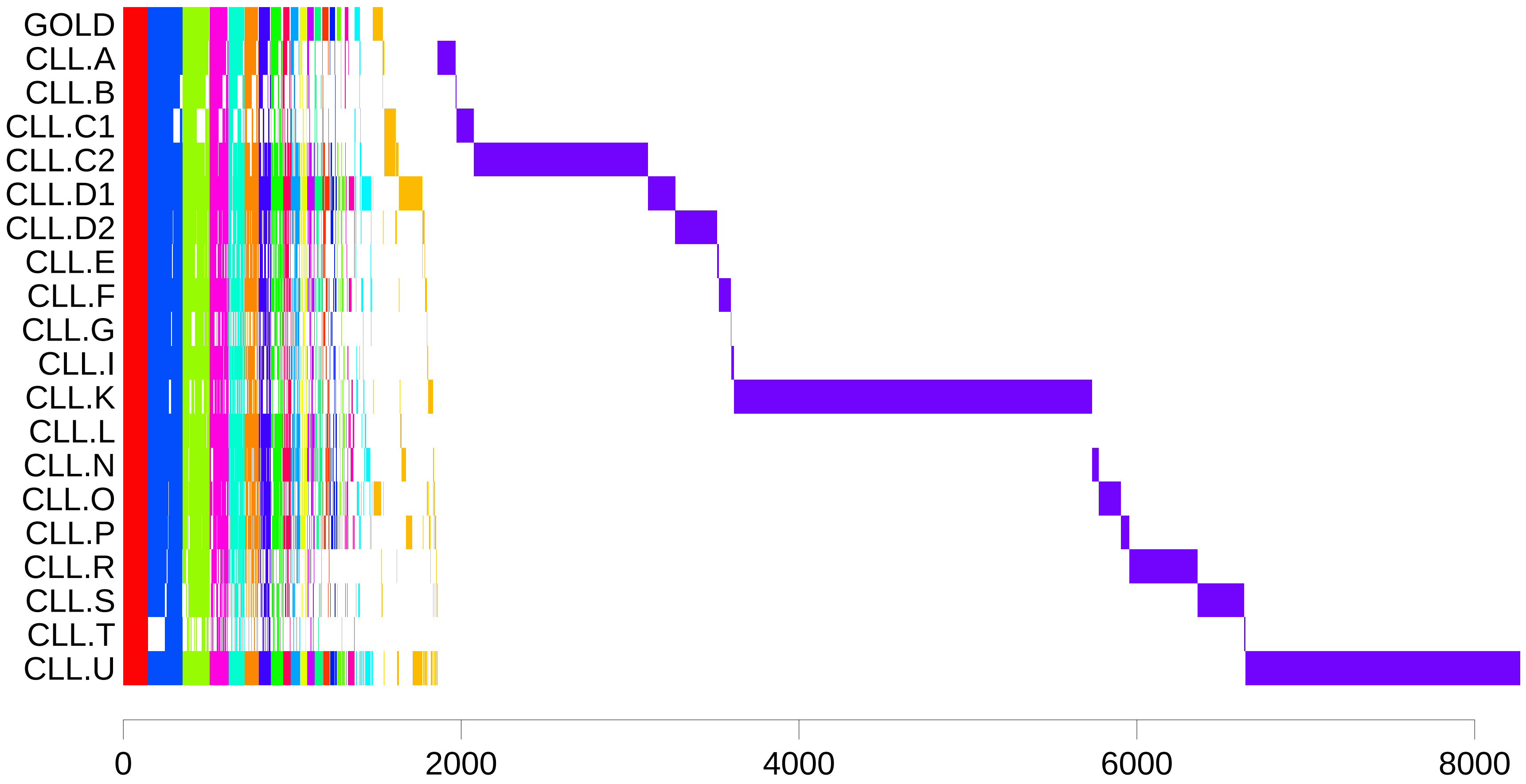

Supplementary Figure 23. Overlap of CLL SSM calls for each level of concordance. Shared sets of calls are vertically aligned. GOLD indicates the Gold Set.

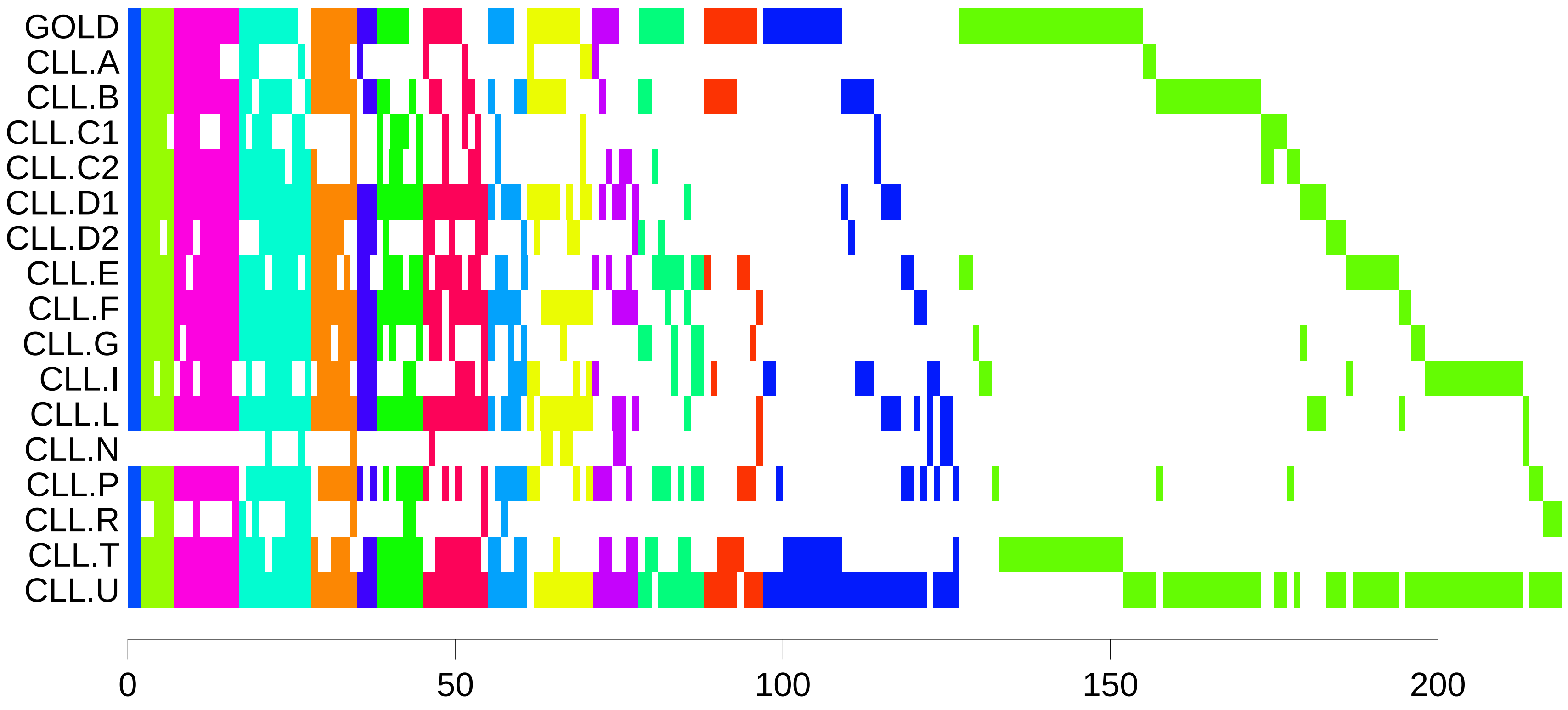

Supplementary Figure 24. Overlap of CLL SIM calls shared by at least two call sets for each level of concordance. Shared sets of calls are vertically aligned. GOLD indicates the Gold Set.

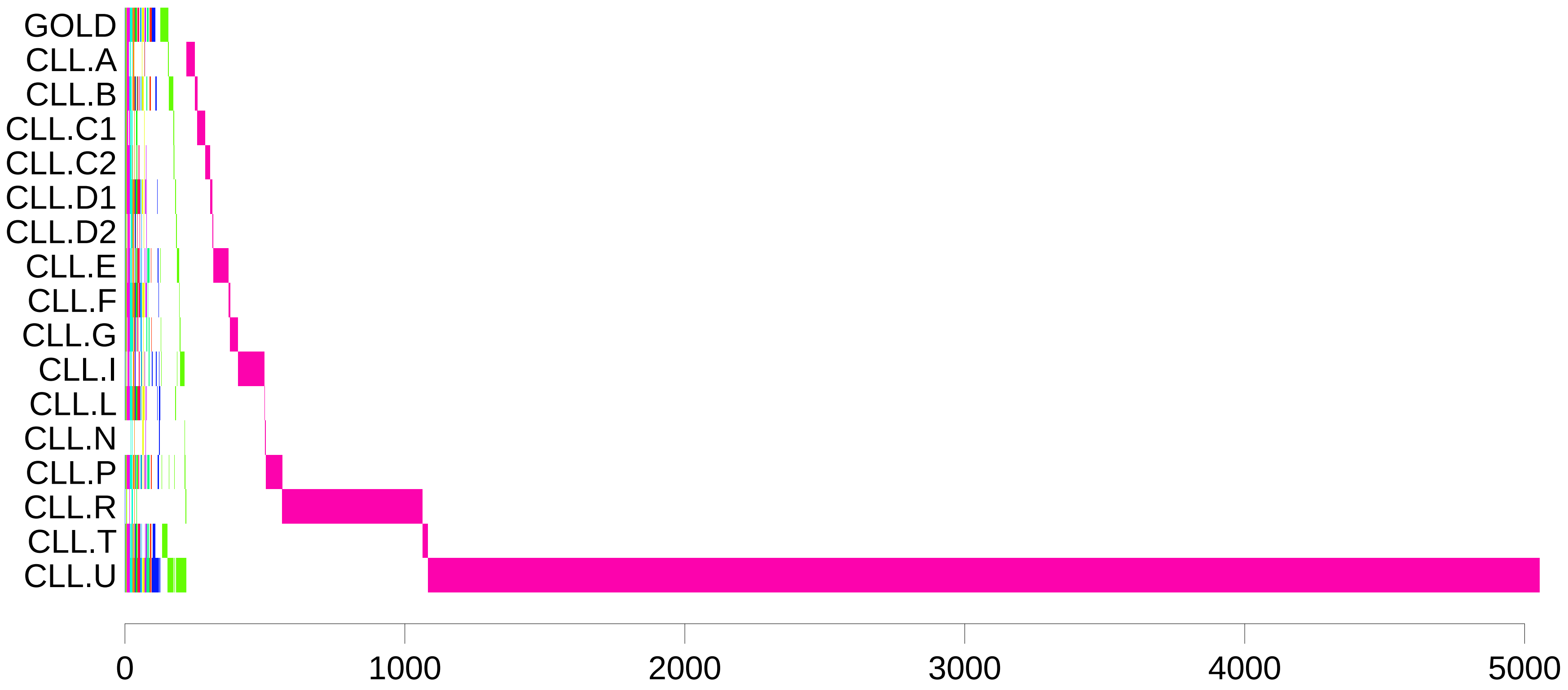

Supplementary Figure 25. Overlap of CLL SIM calls for each level of concordance. Shared sets of calls are vertically aligned. GOLD indicates the Gold Set.

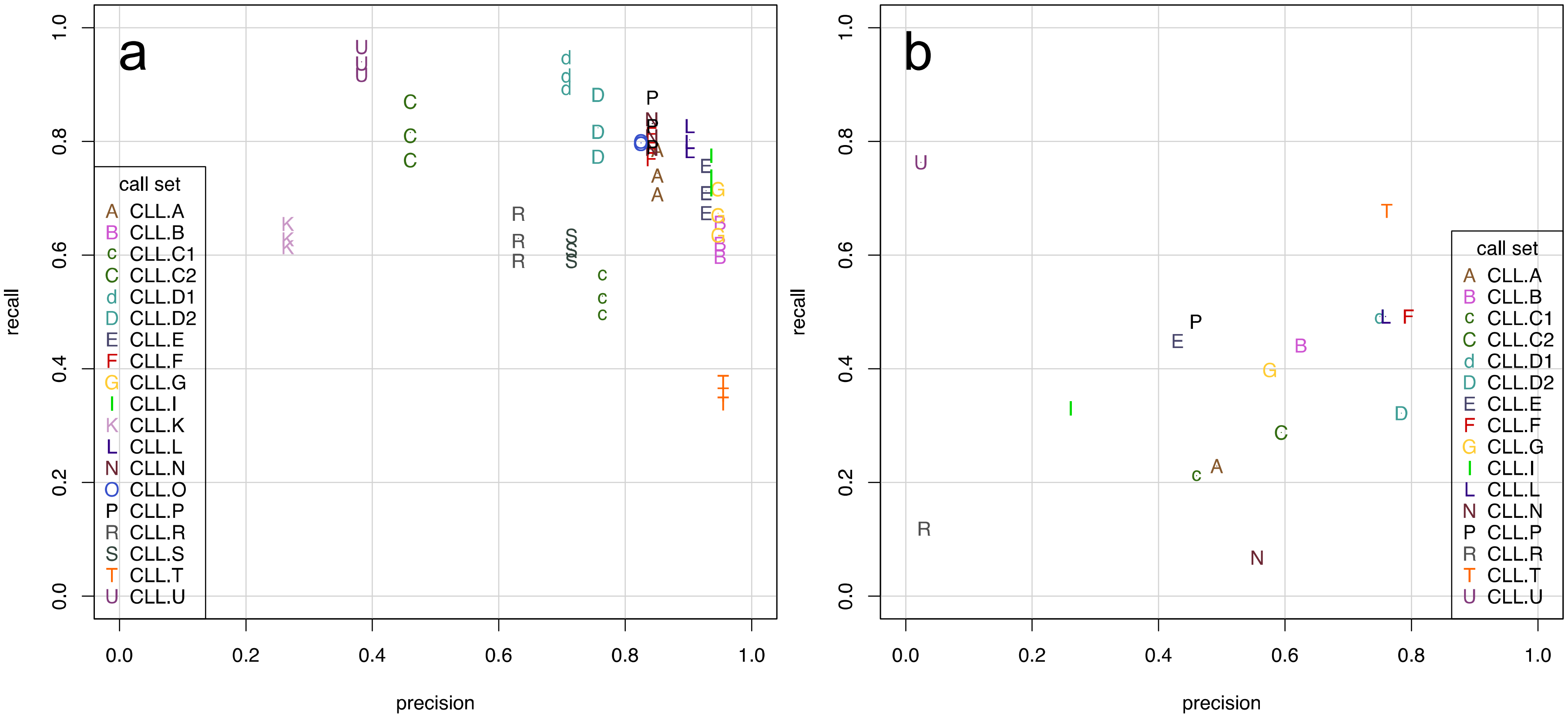

Supplementary Figure 26. Somatic mutation calling accuracy against CLL Gold Set. Decreasing sensitivity on Tiers 1, 2, and 3 shown as series for each SSM call set, while precision remains the same. a) CLL SSMs. b) CLL SIMs.

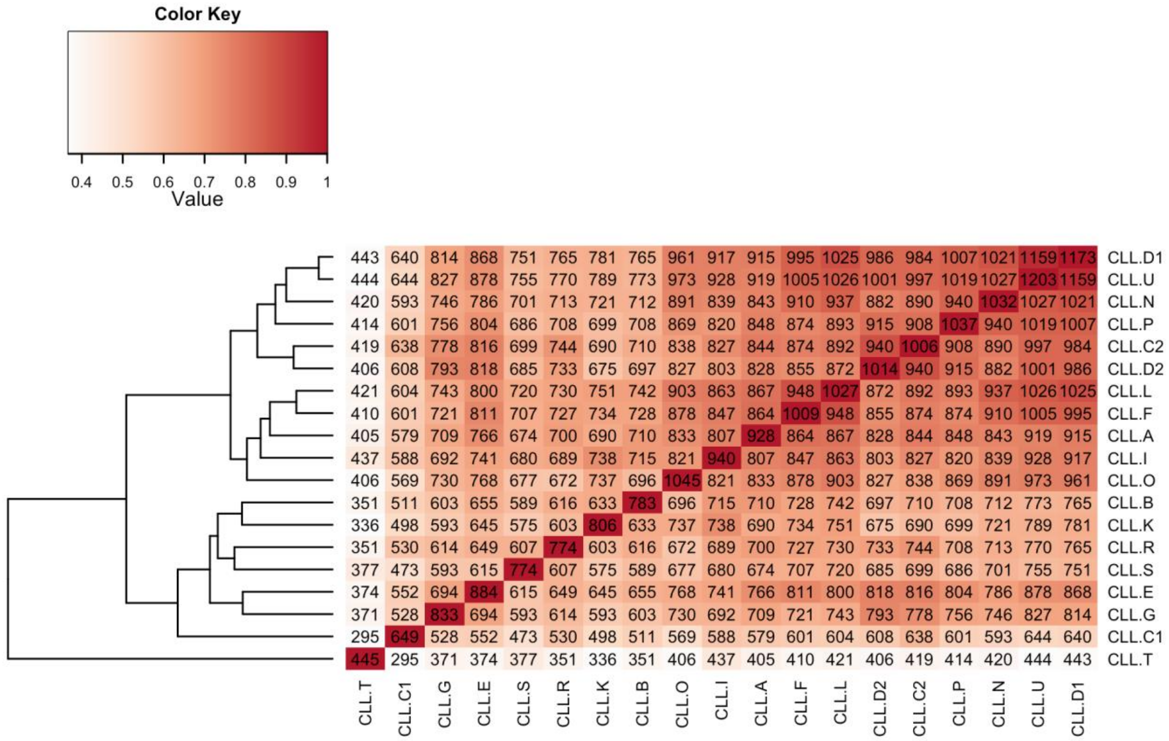

Supplementary Figure 27. Clustering of CLL SSM true positives based on Jaccard index.

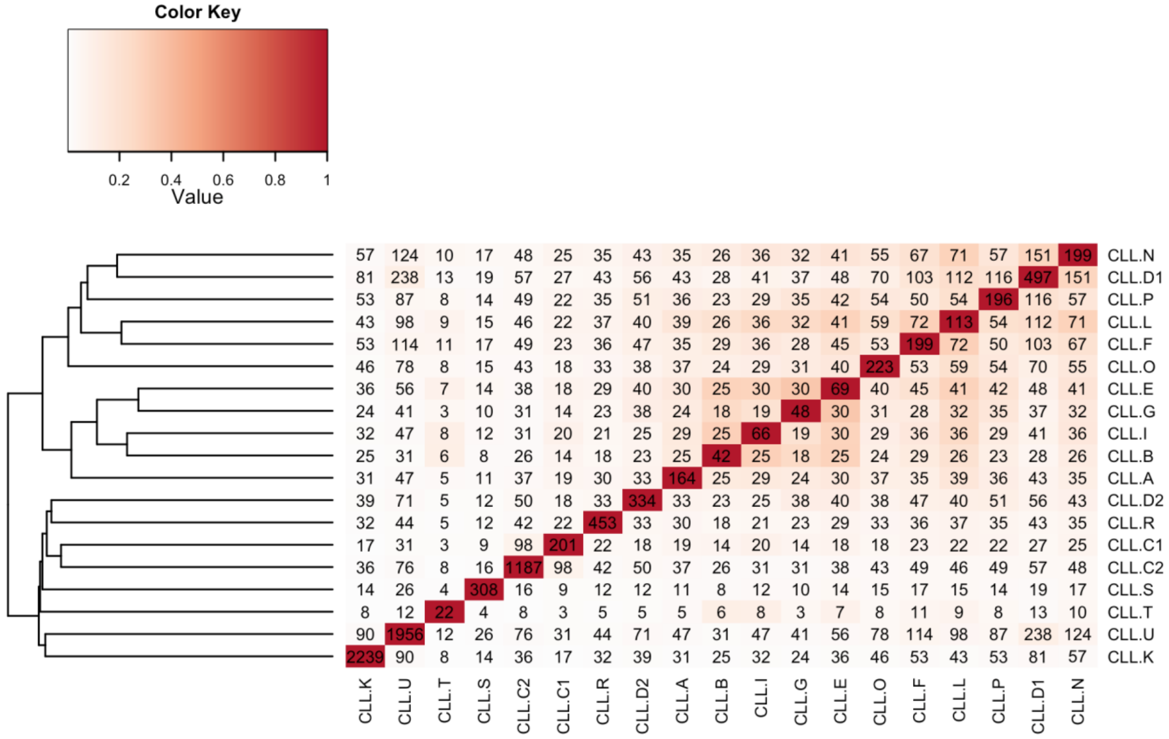

Supplementary Figure 28. Clustering of CLL SSM false positives.

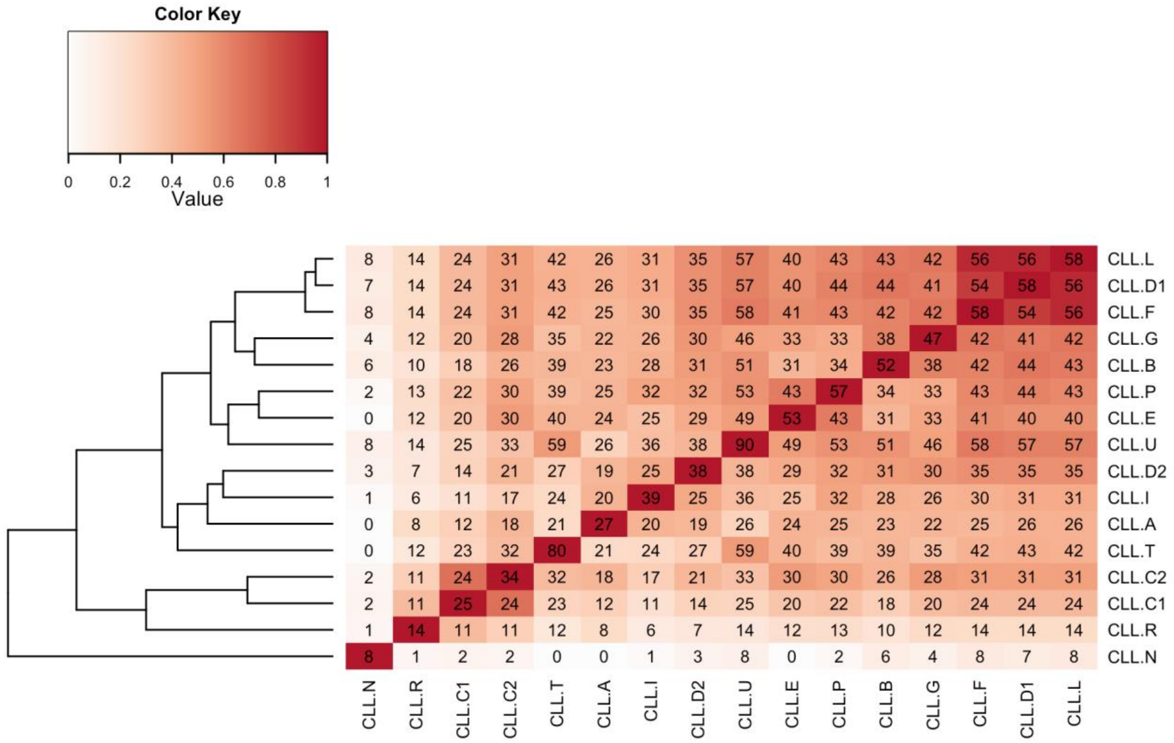

Supplementary Figure 29. Clustering of CLL SIM true positives.

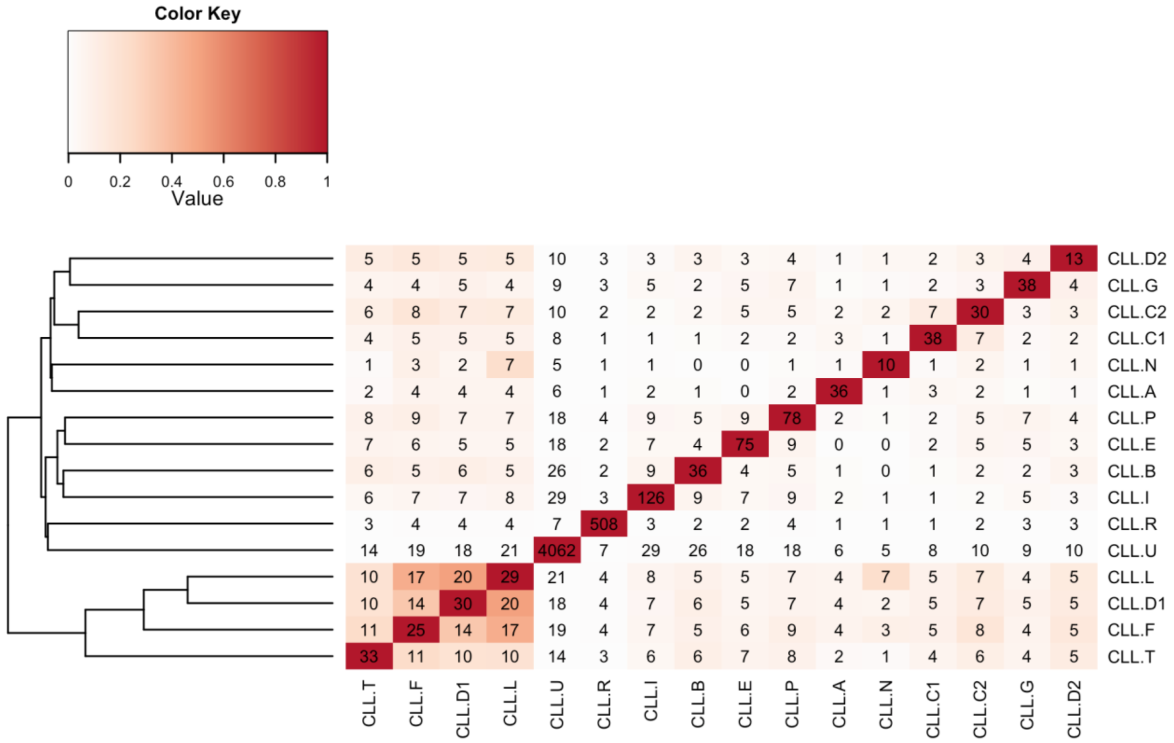

Supplementary Figure 30. Clustering of CLL SIM false positives.

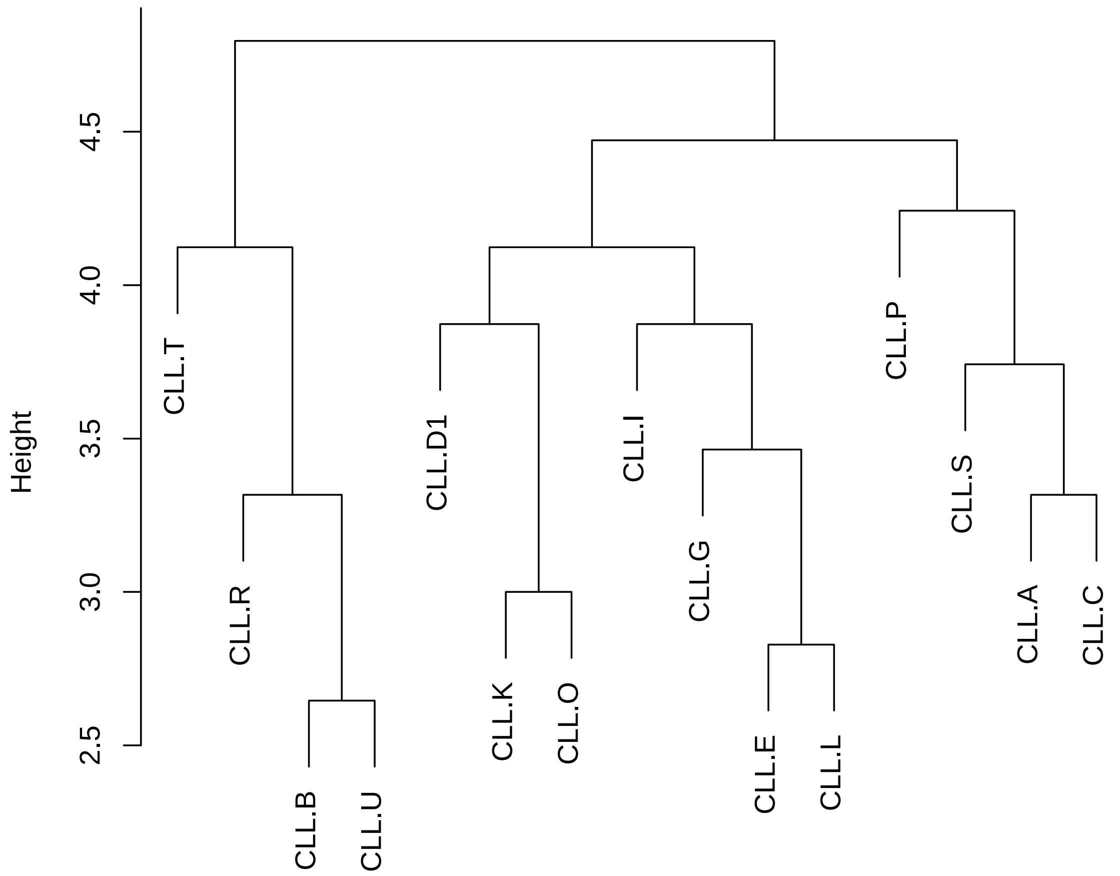

Supplementary Figure 31. Hierarchical clustering of pipeline settings for CLL submissions.

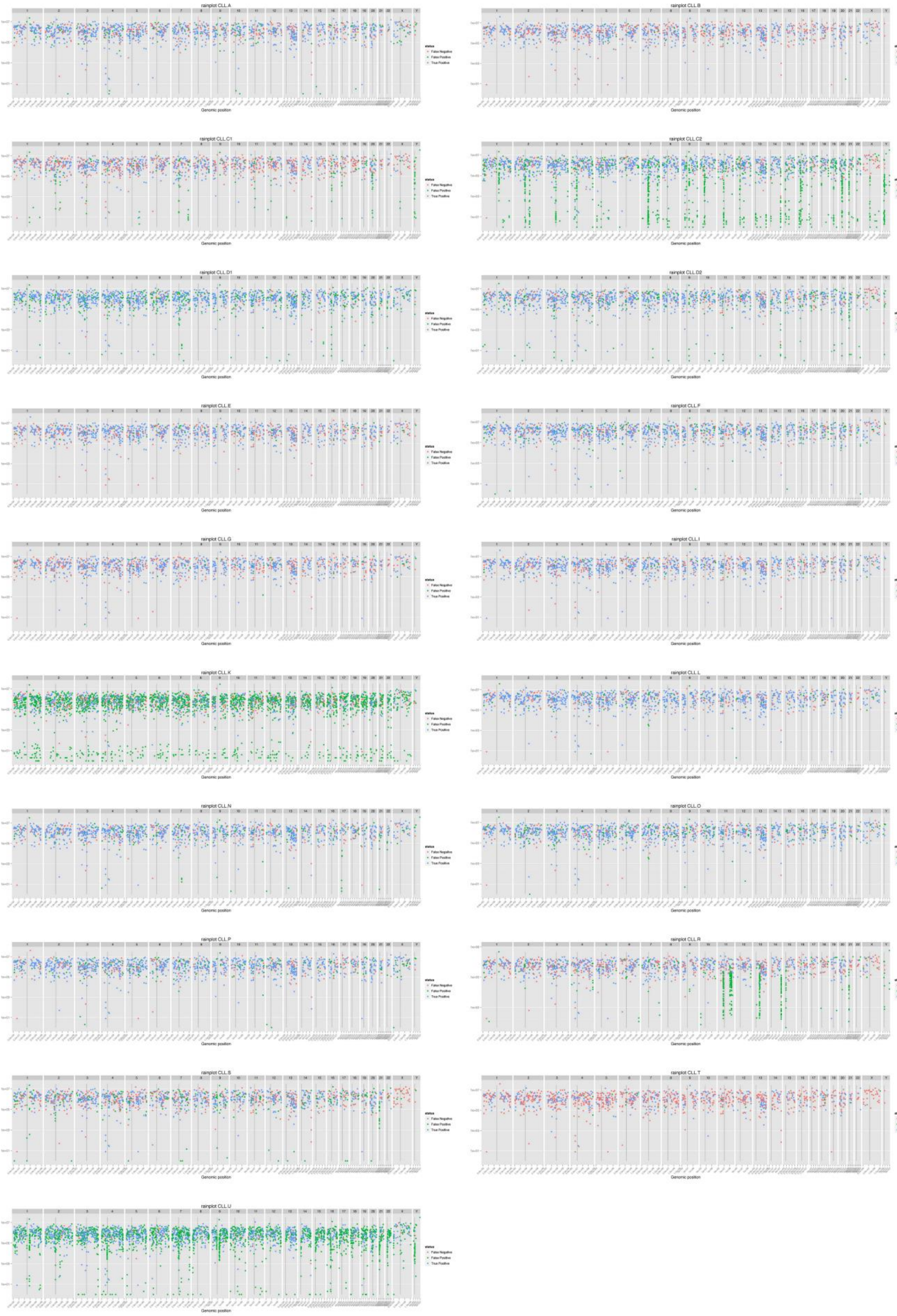

Supplementary Figure 32. Rainfall plots of CLL submissions. False negatives in red, false positives in green, true positives in blue.

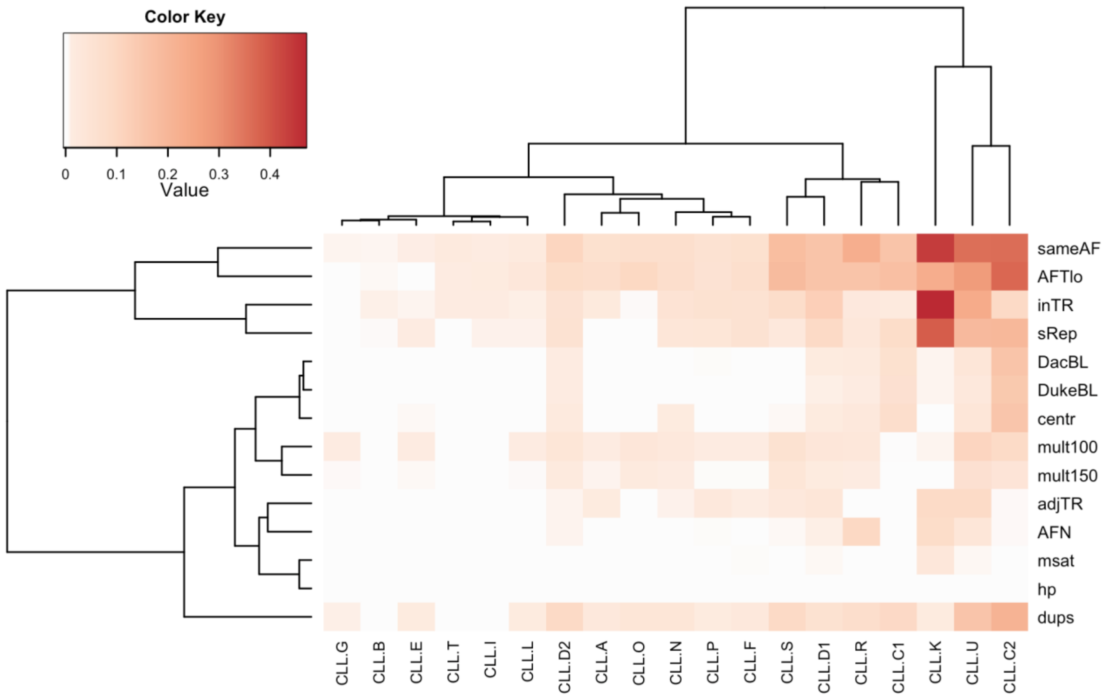

Supplementary Figure 33. Enrichment or depletion of genomic and alignment features in false positive calls for each CLL SSM submission. For each feature, the difference in frequency with respect to the Gold Set is multiplied by the false positive rate. Blue indicates values less than zero and thus the proportion of mutations or their score on that feature is lower in the false positive set with respect to the true mutations. Reddish colors correspond to a higher proportion of mutations or higher scores for the feature in false positive calls versus the Gold Set. Both features and submissions are clustered hierarchically. The features shown here include sameAF (the probability that the allele frequency in the tumor sample is not higher than that in the normal samples, derived from the snape-cmp-counts score), DacBL (in ENCODE DAC mappability blacklist region), DukeBL (in Encode Duke Mappability blacklist region), centr (in centromere or centromeric repeat), mult100 (1 - mappability of 100mers with 1% mismatch), map150 (1 - mappability of 150mers with 1% mismatch), dups (in high-identity segmental duplication), nestRep (in nested repeat), sRep (in simple repeat), inTR (in tandem repeat), adjTR (immediately adjacent to tandem repeat), msat (in microsatellite), hp (in or next to homopolymer of length >6), AFN (mutant allele frequency in normal) and AFTlo (mutant allele frequency in tumor < 10%). Read depth was not used as a feature for the CLL benchmark.

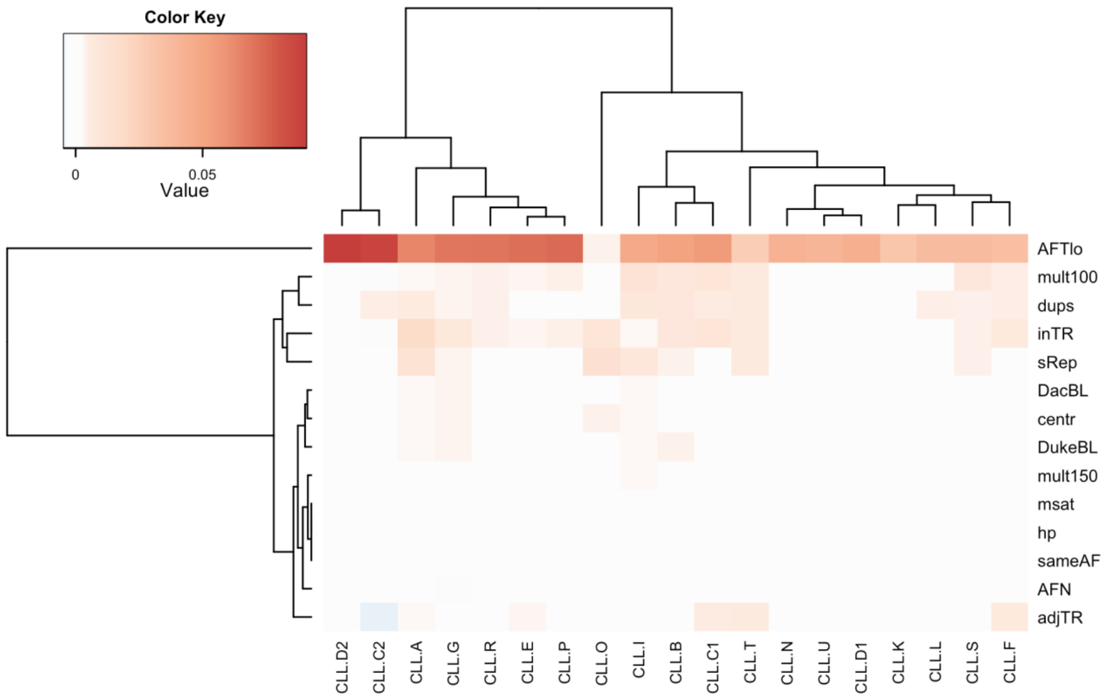

Supplementary Figure 34. Enrichment or depletion of genomic and alignment features in false negative calls for each CLL SSM submission.

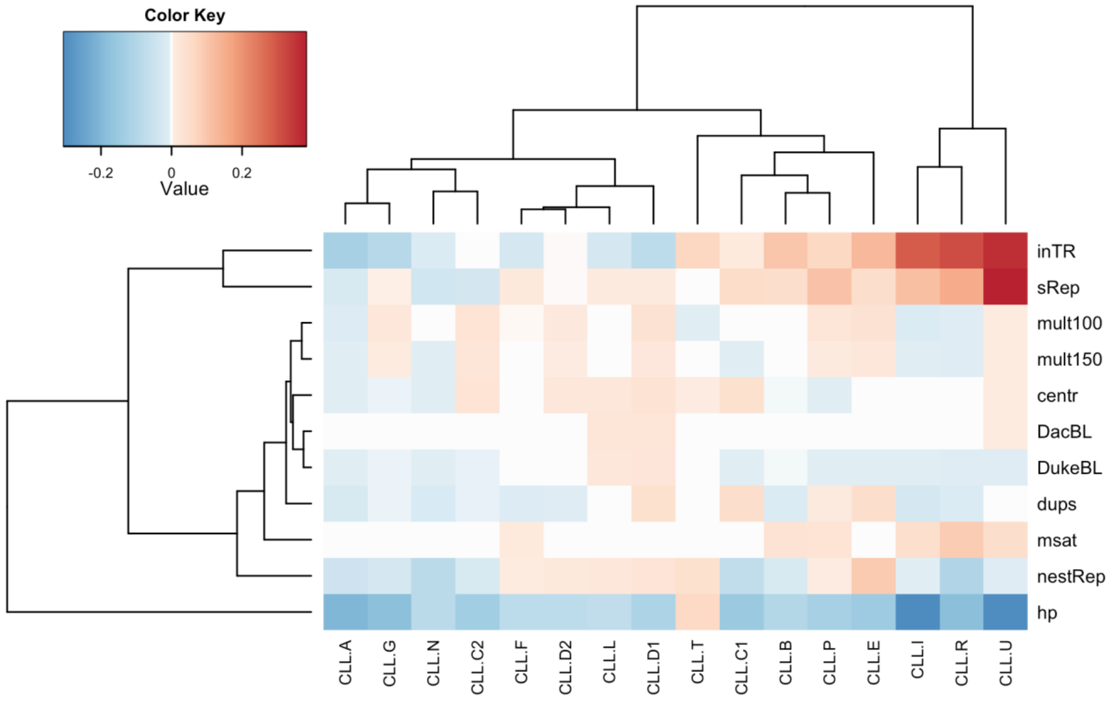

Supplementary Figure 35. Enrichment or depletion of genomic and alignment features in false positive calls for each CLL SIM submission. Allele frequencies for SIMs were not calculated.

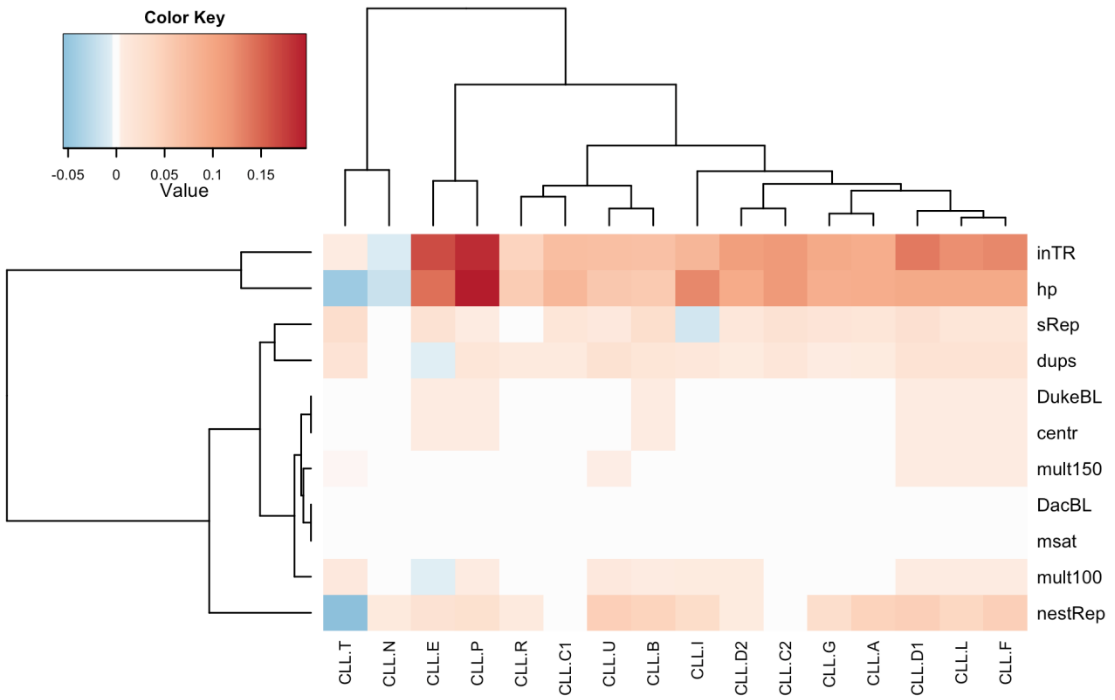

Supplementary Figure 36. Enrichment or depletion of genomic and alignment features in false negative calls for each CLL SIM submission. Allele frequencies for SIMs were not calculated.

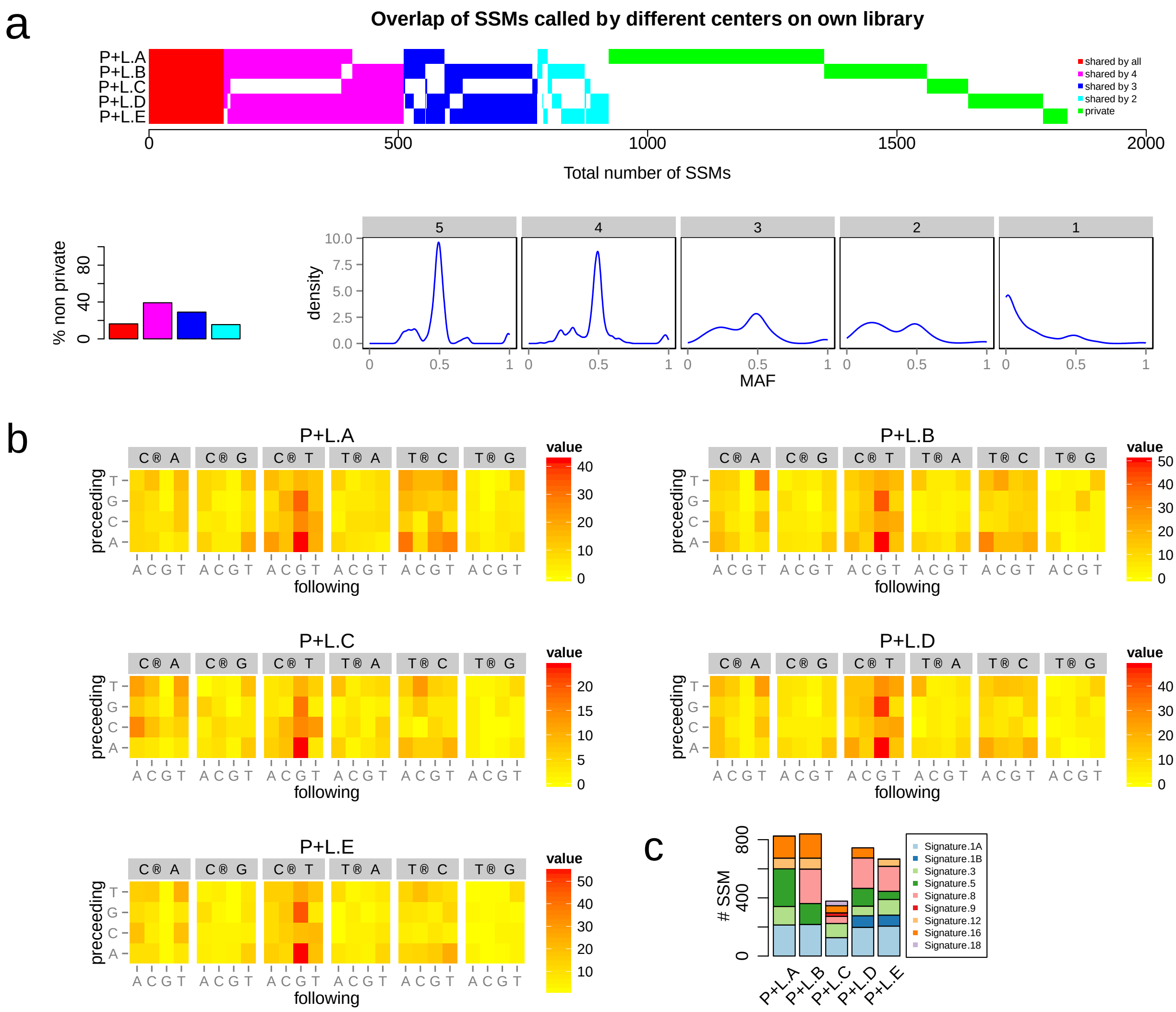

Supplementary Figure 37. Comparison of ability to discover SSMs with different pipelines. **a**) Overlap of SSMs called by each center on its own library. All SSMs detected by at least one center are shown on the x-axis. The SSMs were sorted and colored by recurrence. SSMs were considered to be identical when both the exact position and the base substitution were the same. The bar plot shows the percentage of all non-unique SSMs for the given levels of concordance. Shown on the bottom are the density plots of the variant allele frequencies for each level of concordance. **b**) Sequence context of SSMs detected by each center on its own library. For each single base substitution, the sequence context (plus/minus one base) was determined. The 128 possible combinations are shown in a heat map. **c**) Mutational signatures for SSMs as defined by Alexandrov and colleagues[1]. The calls from each center were used to fit into the predefined signatures. Only signatures composing at least 5% of the total SSMs are shown.

	1	2	3	4	5	6	7	8
GOLD	115,111,0,0<br>114,118,45,46<br>0.28	93,88,1,0<br>70,70,58,70<br>0.48	114,109,1,1<br>79,100,84,92<br>0.5	135,142,1,1<br>105,109,89,107<br>0.48	91,87,1,0<br>70,75,66,61<br>0.47	92,102,1,1<br>1,1,57,54<br>0.98	101,49,0,0<br>125,99,17,8<br>0.1	288,188,1,1<br>204,147,42,27<br>0.16
P+L.A	19,16,0,0<br>17,14,4,4<br>0.21	12,12,1,0<br>9,9,6,12<br>0.5	8,12,0,0<br>13,12,10,7<br>0.4	16,17,0,0<br>8,8,8,7<br>0.48	14,8,0,0<br>2,12,8,10<br>0.56	16,13,0,0<br>0,1,7,3<br>0.91	9,6,0,0<br>19,8,4,3<br>0.21	42,27,0,0<br>24,21,4,2<br>0.12
P+L.B	18,13,0,0<br>15,18,7,9<br>0.33	20,16,0,0<br>17,10,12,10<br>0.45	26,23,1,1<br>23,18,26,19<br>0.52	18,23,1,1<br>15,18,11,20<br>0.48	21,18,1,0<br>20,15,16,17<br>0.49	33,25,1,1<br>0,0,23,14<br>1	23,11,0,0<br>19,35,4,2<br>0.1	39,40,1,0<br>41,32,4,7<br>0.13
P.B+L.F	38,38,0,0<br>40,43,10,7<br>0.17	44,43,0,0<br>27,34,35,22<br>0.48	29,23,0,0<br>26,16,10,7<br>0.29	70,49,0,0<br>27,21,30,29<br>0.55	17,16,0,0<br>6,7,2,10<br>0.48	7,11,0,0<br>0,0,4,11<br>1	69,55,0,0<br>78,59,4,7<br>0.07	64,58,0,0<br>50,28,6,2<br>0.09
P+L.C	45,40,0,0<br>47,37,17,13<br>0.26	31,47,0,0<br>18,36,16,35<br>0.49	49,35,0,0<br>20,34,27,32<br>0.52	84,67,0,0<br>48,59,49,56<br>0.5	19,25,0,0<br>11,14,9,9<br>0.42	19,31,0,0<br>0,0,4,9<br>1	54,20,0,0<br>57,30,5,0<br>0.05	100,45,0,0<br>63,26,13,8<br>0.19
P+L.D	16,20,0,0<br>10,23,7,8<br>0.31	23,9,0,0<br>13,4,10,7<br>0.5	8,18,0,0<br>8,12,10,9<br>0.49	12,23,0,0<br>8,10,6,10<br>0.47	10,16,0,0<br>15,11,12,10<br>0.46	17,18,0,0<br>1,0,9,10<br>0.95	8,10,0,0<br>9,8,2,1<br>0.15	35,34,0,1<br>31,22,8,3<br>0.17
P+L.E	17,22,0,0<br>25,24,9,12<br>0.3	7,4,0,0<br>13,11,13,6<br>0.44	23,17,0,0<br>14,21,11,24<br>0.5	5,11,0,0<br>24,14,14,14<br>0.42	27,19,0,0<br>22,20,20,14<br>0.45	6,11,0,0<br>0,0,14,17<br>1	6,2,0,0<br>19,17,2,2<br>0.1	68,41,0,0<br>42,46,11,7<br>0.17
	chr12 : 51126224 C –> A DIP2B nonsynonymous SSM	chr19 : 39068675 G –> A RYR1 nonsynonymous SSM	chr9 : 123929810 A –> T CNTRL nonsynonymous SSM	chr14 : 90650899 C –> G KCNK13 nonsynonymous SSM	chr9 : 131456174 G –> T SET splicing	chr8 : 108296989 C –> A ANGPT1 stopgain SSM	chr3 : 73433313 C –> T PDZRN3 nonsynonymous SSM	chr1 : 144879387 G –> A PDE4DIP stopgain SSM

	9	10	11	12	13	14	15	16
GOLD	136,147,0,0<br>161,196,8,10<br>0.05	106,102,0,0<br>133,99,5,5<br>0.04	110,112,42,131<br>155,161,30,145<br>0.36	112,63,0,2<br>170,104,5,13<br>0.06	44,48,0,0<br>60,53,52,29<br>0.42	99,81,0,0<br>131,71,1,0<br>0	71,122,0,0<br>63,71,1,1<br>0.01	66,57,7,8<br>96,100,17,15<br>0.14
P+L.A	18,16,0,0<br>20,20,0,2<br>0.05	15,12,0,0<br>24,11,1,1<br>0.05	22,4,0,2<br>14,9,2,10<br>0.34	16,8,0,1<br>19,17,1,1<br>0.05	7,9,0,0<br>2,5,2,0<br>0.22	10,5,0,0<br>14,5,0,0<br>0	7,14,0,0<br>10,4,0,0<br>0	4,6,0,0<br>13,9,0,0<br>0
P+L.B	26,32,0,0<br>35,33,3,2<br>0.07	24,25,0,0<br>28,28,0,1<br>0.02	37,19,8,70<br>34,34,3,76<br>0.54	23,7,0,0<br>29,18,1,4<br>0.1	10,10,0,0<br>11,11,7,2<br>0.29	13,16,0,0<br>30,20,0,0<br>0	24,23,0,0<br>15,23,0,0<br>0	19,12,2,2<br>17,16,8,3<br>0.25
P.B+L.F	19,24,0,0<br>19,27,0,3<br>0.06	27,13,0,0<br>16,8,0,1<br>0.04	11,9,7,19<br>13,10,6,5<br>0.32	70,58,3,9<br>81,80,5,5<br>0.06	37,25,0,0<br>27,16,27,9<br>0.46	42,30,0,0<br>31,24,0,0<br>0	17,35,0,0<br>21,15,0,0<br>0	55,43,5,12<br>45,60,3,8<br>0.09
P+L.C	31,44,0,0<br>41,71,2,3<br>0.04	32,14,0,0<br>33,13,1,0<br>0.02	20,24,6,48<br>34,39,9,40<br>0.4	57,39,0,1<br>78,42,1,7<br>0.06	22,9,0,0<br>27,17,28,9<br>0.46	44,34,0,0<br>25,15,1,0<br>0.02	21,31,0,0<br>20,23,0,0<br>0	27,24,3,5<br>32,44,5,5<br>0.12
P+L.D	25,23,0,0<br>22,29,2,1<br>0.06	20,25,0,0<br>18,13,2,1<br>0.09	14,35,5,4<br>33,26,4,8<br>0.17	11,8,0,0<br>7,9,2,0<br>0.11	5,8,0,0<br>7,5,4,8<br>0.5	19,11,0,0<br>22,13,0,0<br>0	14,21,0,0<br>3,4,1,1<br>0.22	8,7,0,0<br>13,6,2,2<br>0.17
P+L.E	33,27,0,0<br>39,41,1,2<br>0.04	13,24,0,0<br>29,33,1,2<br>0.05	18,17,15,2<br>37,35,3,5<br>0.1	5,1,0,0<br>33,16,1,2<br>0.06	0,12,0,0<br>8,11,8,7<br>0.44	13,15,0,0<br>36,18,0,0<br>0	3,32,0,0<br>15,17,0,0<br>0	7,5,0,1<br>16,21,1,4<br>0.12
	chr16 : 81077733 C –> A ATMIN nonsynonymous SSM	chr11 : 105797546 G –> A GRIA4 nonsynonymous SSM	chr2 : 97808394 C –> G ANKRD36 nonsynonymous SSM	chr19 : 50881825 G –> A NR1H2 nonsynonymous SSM	chrX : 70469481 C –> T ZMYM3 nonsynonymous SSM	chr1 : 65129464 C –> A CACHD1 nonsynonymous SSM	chr8 : 20003354 G –> A SLC18A1 nonsynonymous SSM	chr11 : 117789327 T –> C TMPRSS13 nonsynonymous SSM

7 Low variant count but nothing obvious in the region
8 Overlap with segmental duplication track and high seq depth track
9 Overlap with segmental duplication track and low variant allele count
10 Very low variant count, removed by raw filter
11 Strong strand bias. Call absent when aligned to hg37d5 reference genome
12 Low variant count and known SNP position (possible artifact or germline)
13 Variant present but probably bigger structural event (medium sized indel)
14 Variant only present in this sample (possibly due to different reference genome)
15 Low frequency variant present only in this sample
16 Overlap with tandem repeat and present in germline

Supplementary Figure 38. Coding mutations found by the different submitters and read support in BWA aligned bam files.

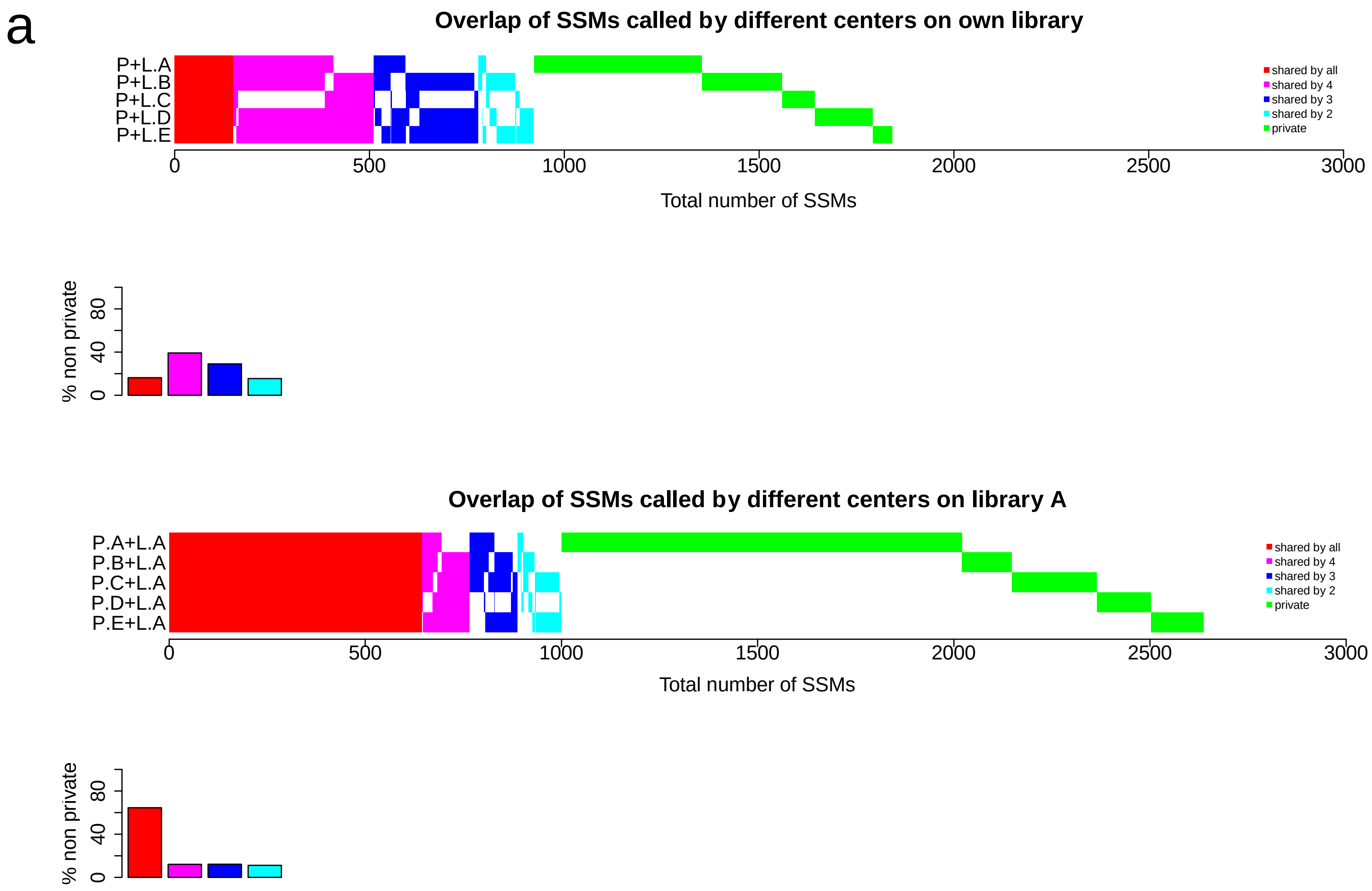

Supplementary Figures 39. Overlap levels of SSMs called by different centers on their own (different) and on one uniform (library A) sample.

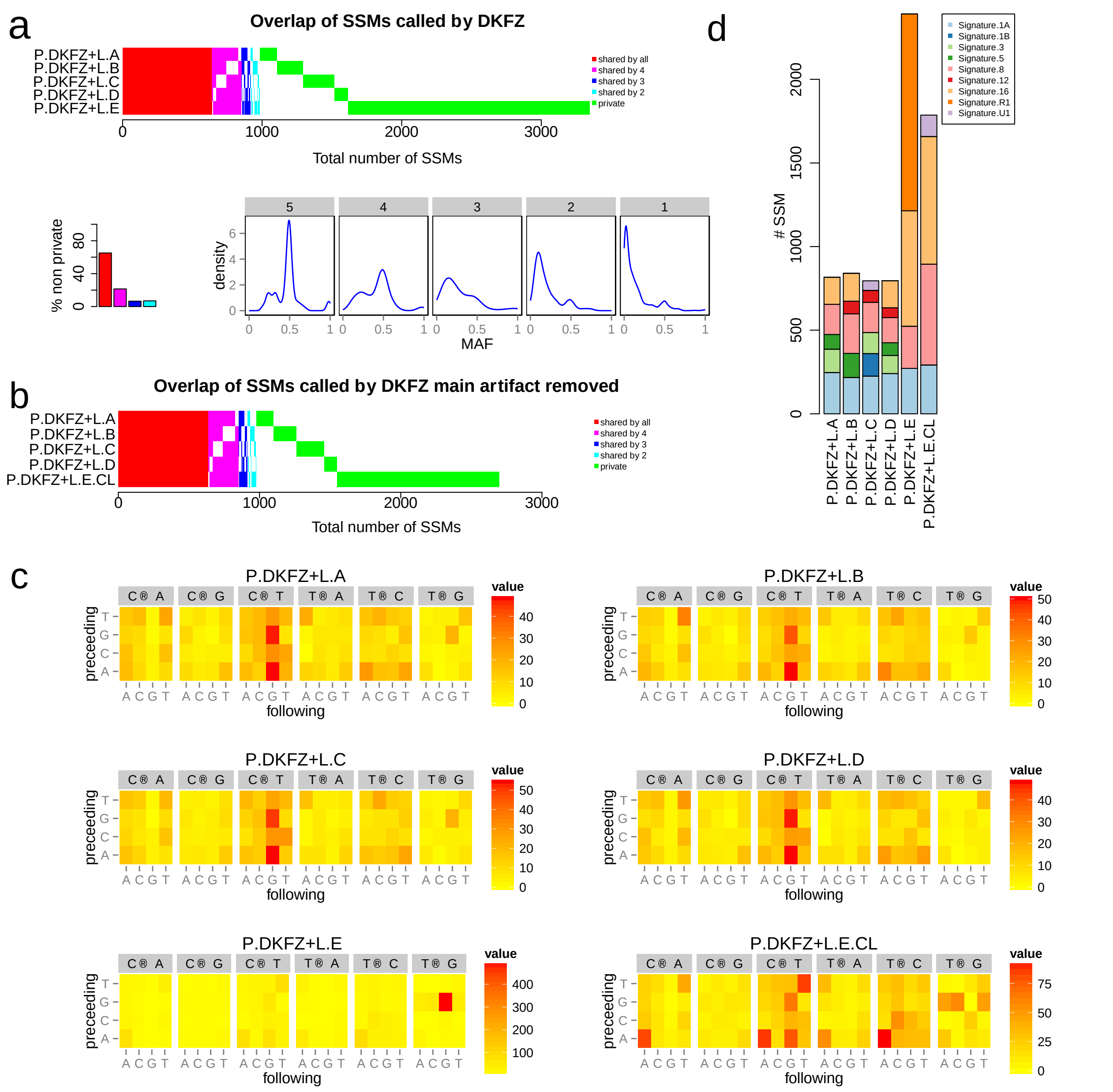

Supplementary Figure 40. Influence of the library and sequencing on SSM calls with one pipeline. **a**) Overlap of SSMs called using one pipeline (DKFZ) on all different data sets. Percentage of concordance of non-unique SSMs are shown in the bar plot. The bottom shows density plots of variant allele frequencies for each concordance level. **b**) Overlap of SSMs called after removal of the most prominent artifact (GpTpG to GpGpG) from the library L.E. **c**) Sequence context of SSM calls derived from the DKFZ pipeline on the different data sets. In order to have a better comparability, the most prominent artifact was removed from L.E (L.E.CL). **d**) Mutational signatures for SSMs as defined by Alexandrov and colleagues[1]. Calls made by DKFZ were fitted to the predefined signatures. Only signatures composing at least 5% of the total SSMs are shown

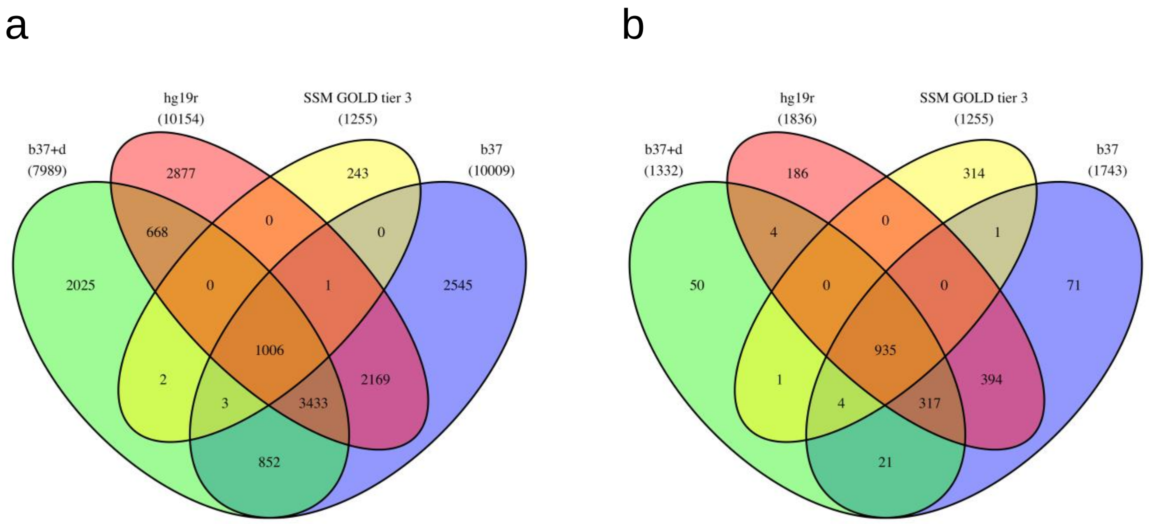

Supplementary Figure 41. Sizes (in parentheses) and overlaps of SSM call sets produced by MuTect (a) and Strelka (b). SSM calling was performed on Novoalign2 alignments against three different human reference genome builds, and the tier 3 SSM Gold Set was used for validation.

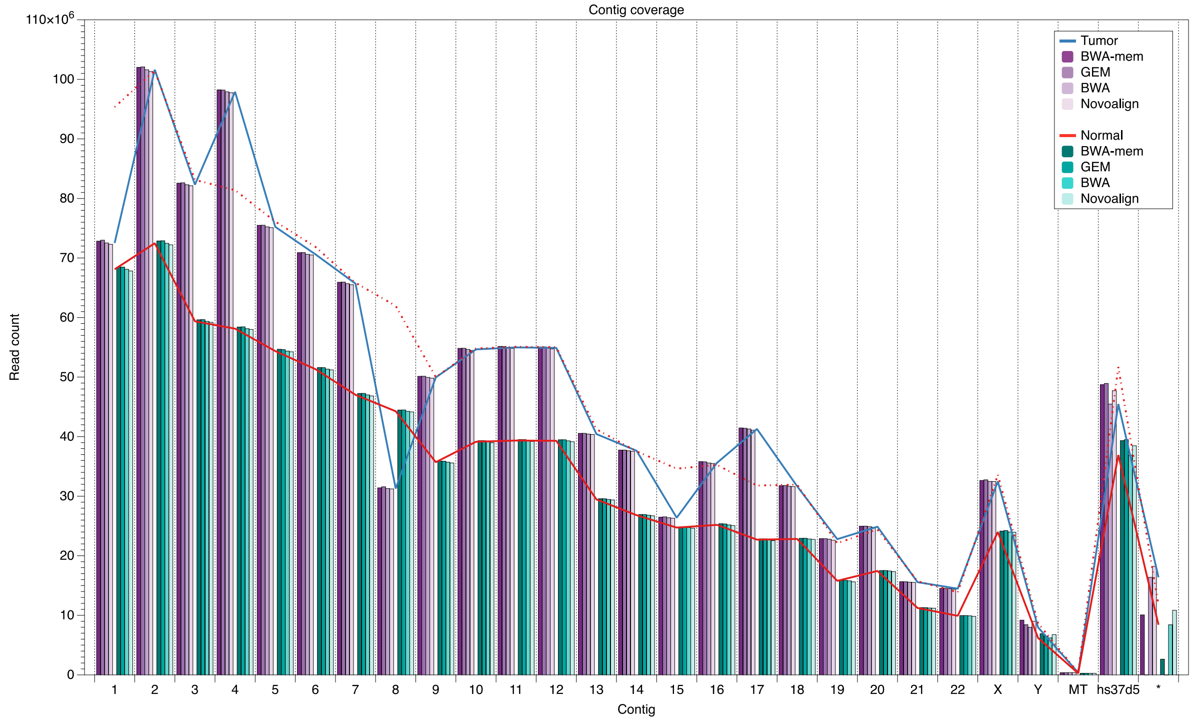

Supplementary Figure 42. Counts of reads aligned to individual reference genome chromosomes by the individual mappers (using qprofiler's "RNAME" SAM-field statistics). Clear differences can be spotted for chromosome Y and the decoy contig (hs37d5). General copy number differences between the tumor and its control sample can be seen on the plot. The blue line tracks the number of tumor sample reads while the red lines track the number of control sample reads (full line for the observed counts, dotted line for the observed counts normalized to tumor sample size). Big deletion events are apparent on chromosomes 1, 8 and 15, big duplication events on chromosomes 4 and 17.

a

b

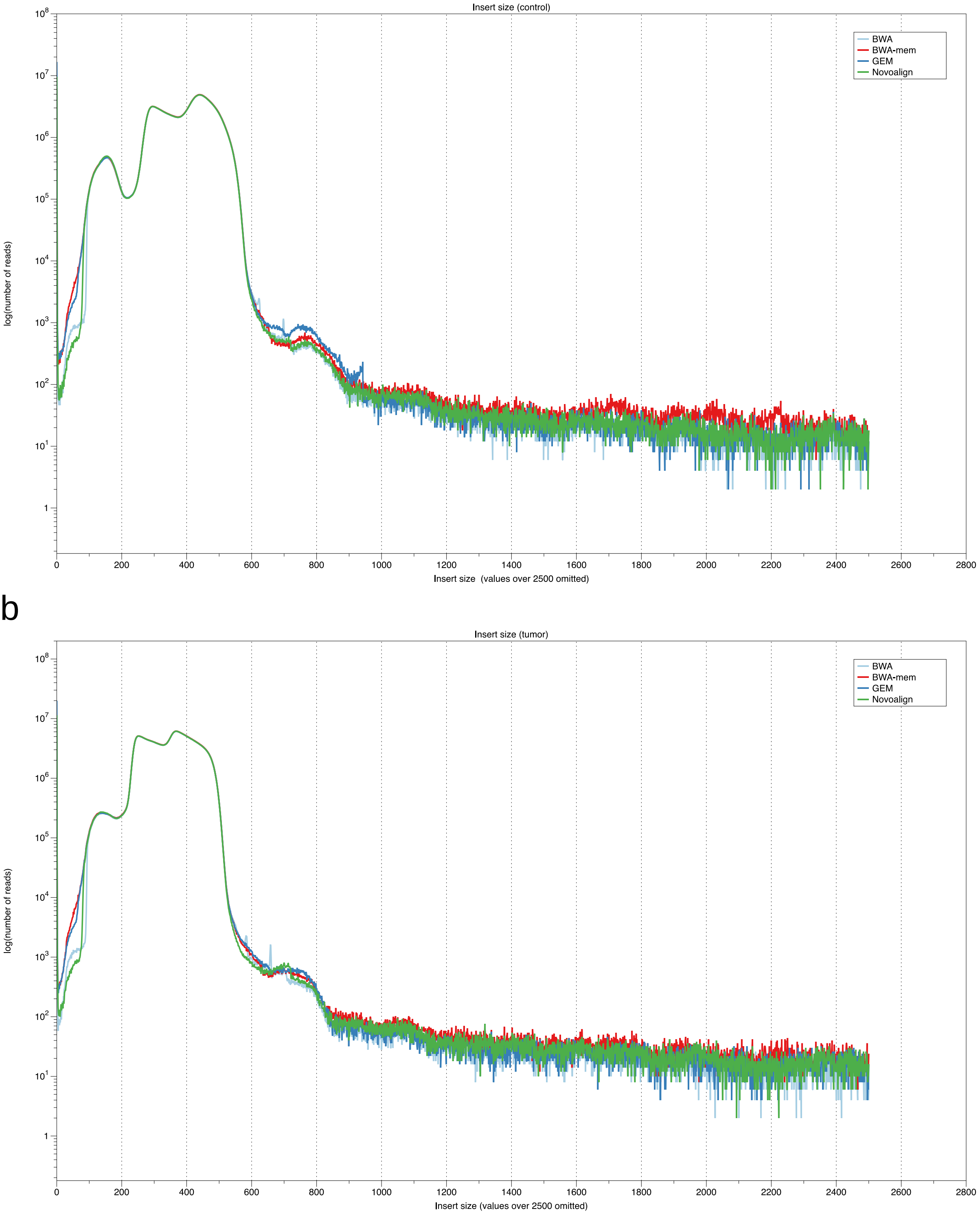

Supplementary Figure 43. Observed insert size distributions for alignments produced by the individual mappers. Good overall agreement is visible, especially in the ranges that are expected to contain the bulk of proper pairs.

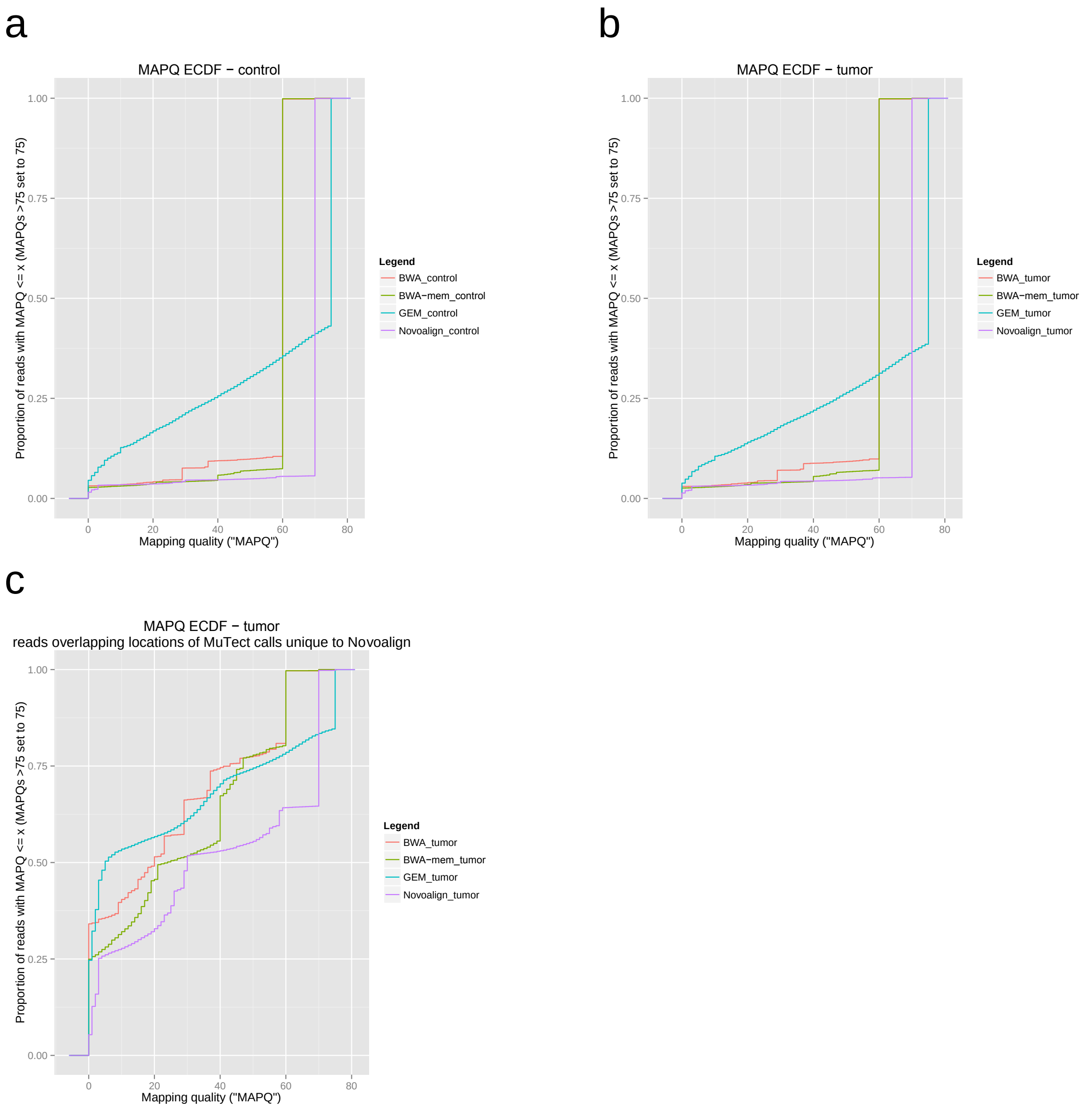

Supplementary Figure 44. Empirical cumulative distribution functions of mapping quality (MAPQ) values assigned to individual read mappings from chromosomes 1-22, X and Y for the control (a) and tumor (b) and in (c) MAPQ values of tumor read mappings that overlap locations of MuTect SSM calls unique to Novoalign2. This plot illustrates a possible contributing factor to the high observed number of false positive calls being made by MuTect on Novoalign2 alignments: reads with MAPQ 1-4 rather than 0 (together with a higher fraction of reads with MAPQ=>20) do not trigger MuTect’s filter aimed at discovering false positive calls caused by misplaced reads.

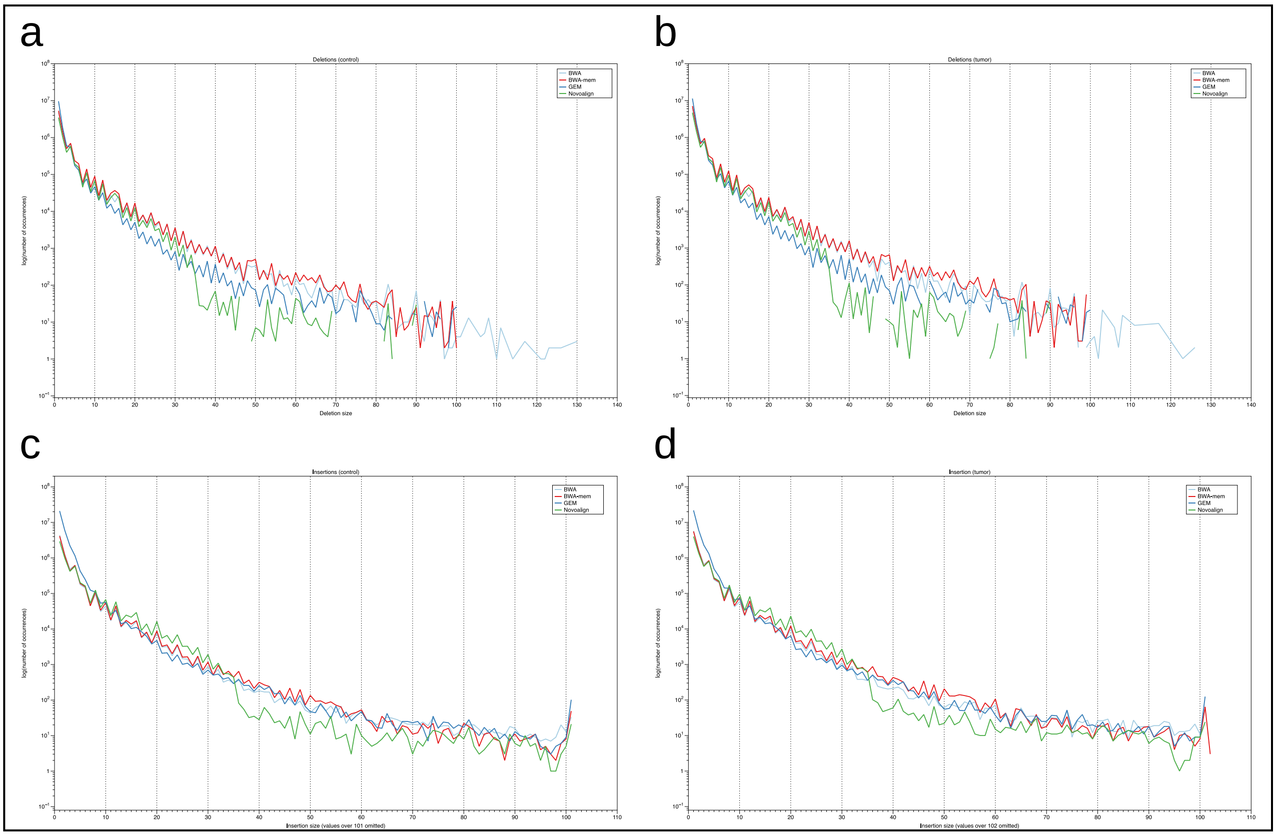

Supplementary Figure 45. Plots based on qprofiler's "CIGAR" SAM-field statistics. Deletion statistics for the control (a) and the tumor (b), as well as insertion statistics for the control (c) and the tumor (d) are plotted for each of the four mappers. GEM alignments contain slightly fewer deletion events (of size at least 15 bp), while for Novoalign2 alignments a similar trend is evident for insertions and deletions with size of at least 35 bp. The latter phenomenon might be caused by Novoalign2 trimming its input reads to 150 bp in length by default.

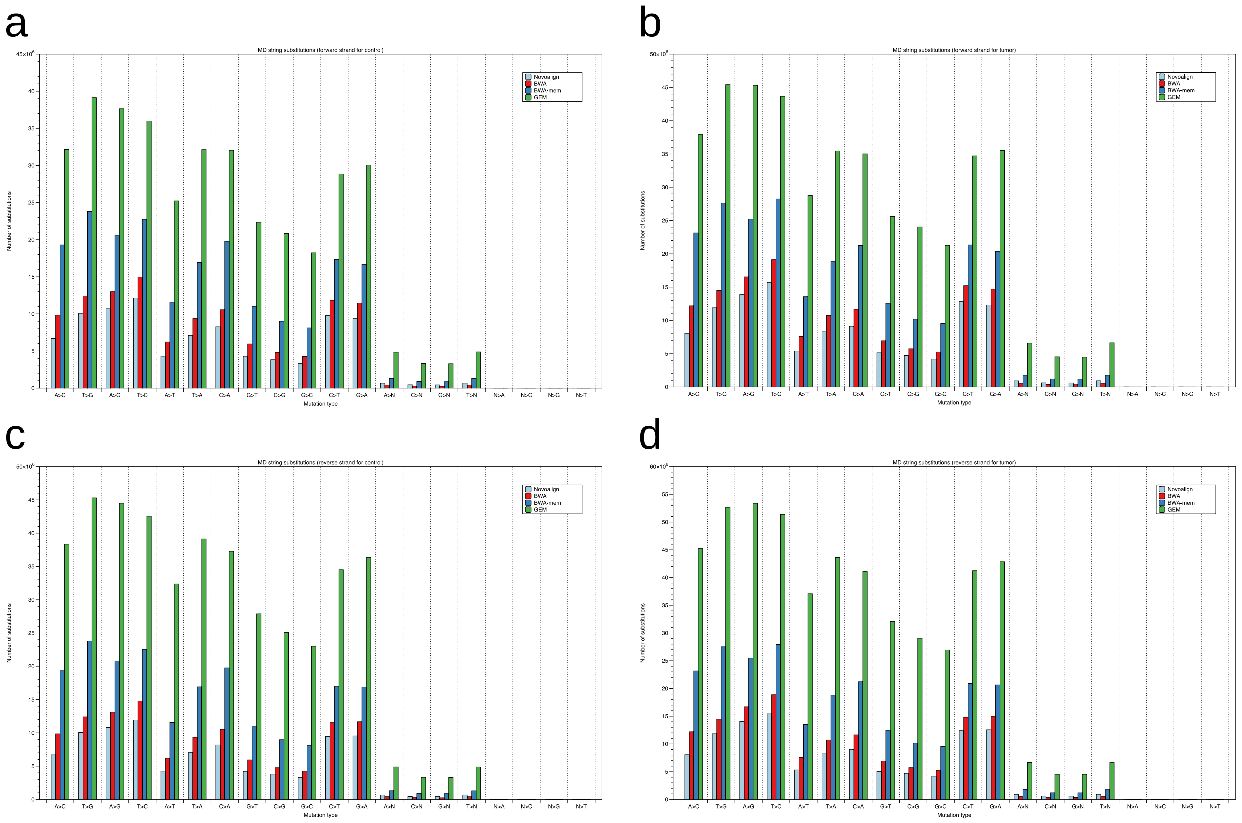

Supplementary Figure 46. For each mapper, the counts of mismatches of different types are shown (using qprofiler's "MD" SAM-field statistics). Consistent differences between the mappers can be observed – for all non-ambiguous substitution types, the counts are highest for GEM alignments, with BWA-mem, BWA and Novoalign2 following always in the same order.

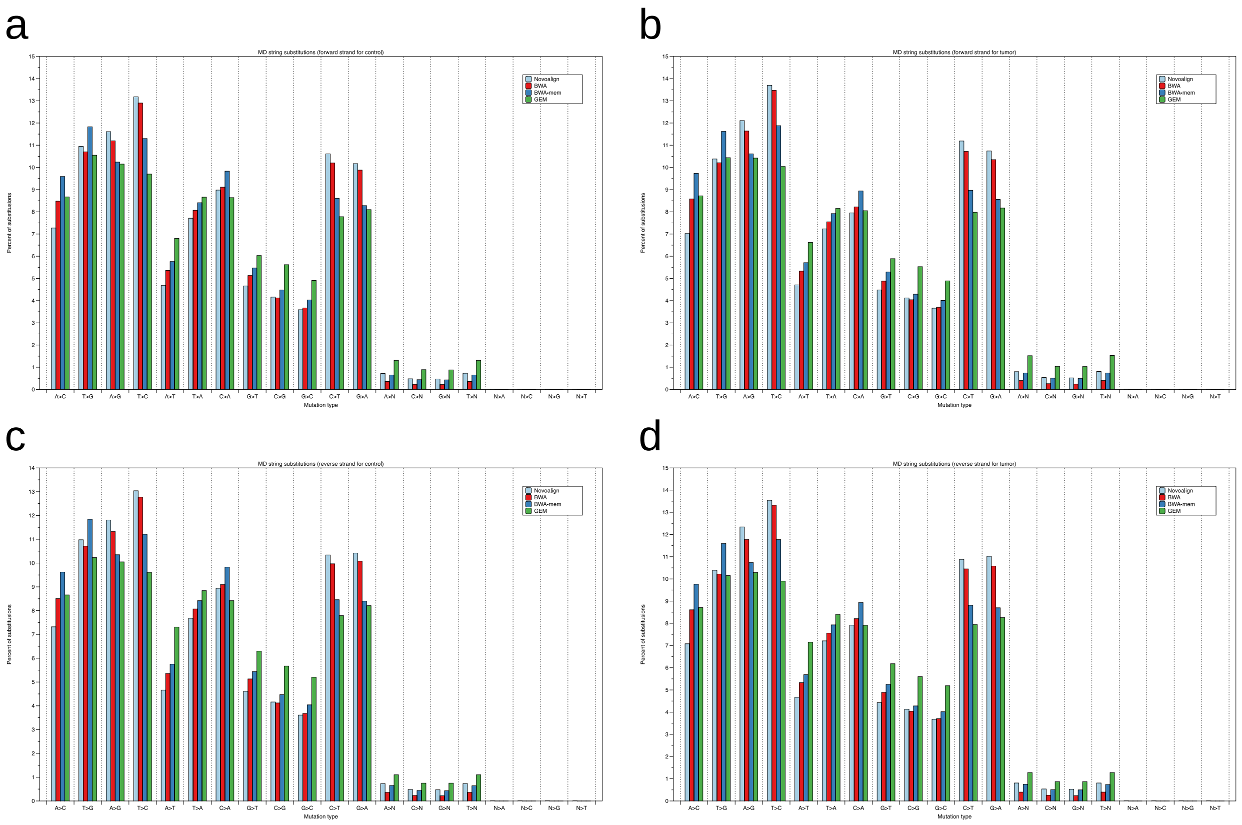

Supplementary Figure 47. For each mapper, the ratios of mismatches of different types are shown (using qprofiler's "MD" SAM-field statistics). Interestingly, some symmetrical mismatches are well balanced (C>T and G>A) while others are not (C>A and G>T). These imbalances are largely mapper-independent. Individual mappers seem to have preferred mutations types (e.g. Novoalign2 alignments contain relatively high fractions of C>T and G>A mismatches and relatively low fraction of A>C mutations, while the situation is reversed for BWA-mem alignments).

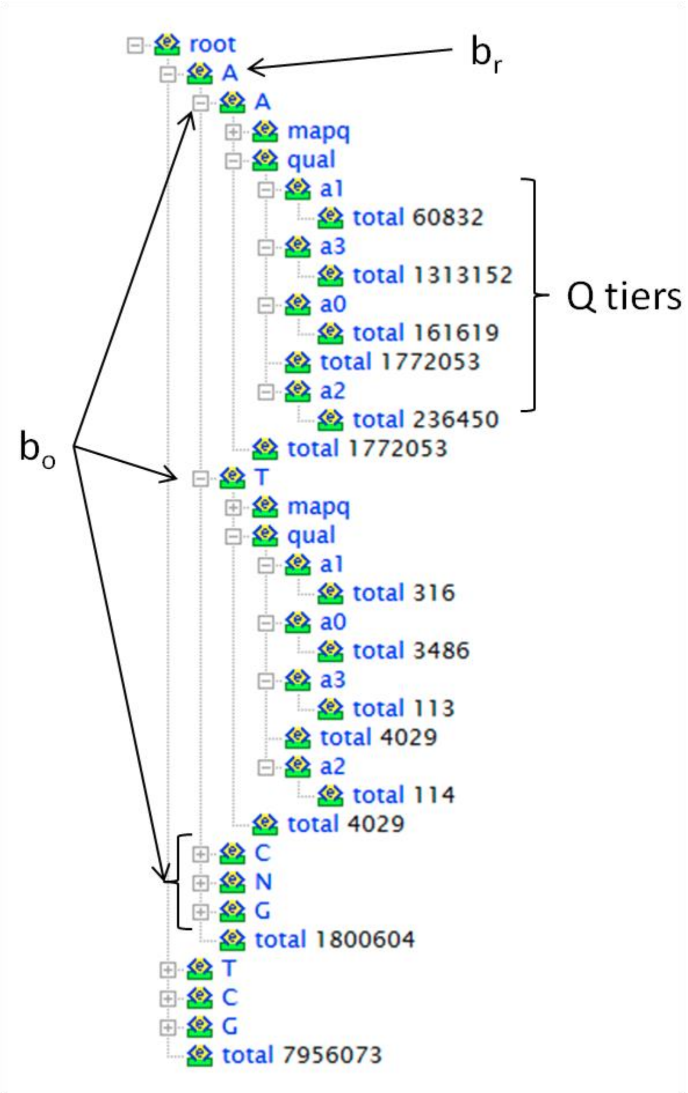

Supplementary Figure 48. An example of part of a Sidron error table (CLL.P and MB.P). In this example, out of the 1,800,604 times that br was "A", the machine read "A" 1,772,053 times (about 96%). However, in 4,029 occasions (0.2%), the machine read "T". As expected, if the quality is low (a0 in the figure), the error frequency is higher. In this case, the machine read "A" 161,319 times and "T" 3,486 times.

Insti-tute	Library	Quanti-fication	Star-ting DNA	Shearing	Fragment Size (bp)	Size selection	Library Protocol	Cleanup steps	PCR	Amount loaded	Cluster-ing	Sequencer	Chemistry	Sequencing mode
**L.A**	Normal	Qubit	4 µg	Covaris	350-500	2% Agarose Gel	KapaBio	beads/std KapaBio	no	17 pM	on board	HiSeq 2500	Rapid	Rapid
**L.A**	Tumor	Qubit	4 µg	Covaris	400-550	2% Agarose Gel	KapaBio	beads/std KapaBio	no	17 pM	on board	HiSeq 2500	Rapid	Rapid
**L.A**	Normal	Qubit	4 µg	Covaris	350-500	2% Agarose Gel	KapaBio	beads/std KapaBio	no	21.4 pM	cBot	HiSeq 2000	V3	High output
**L.A**	Tumor	Qubit	4 µg	Covaris	400-550	2% Agarose Gel	KapaBio	beads/std KapaBio	no	21.4 pM	cBot	HiSeq 2000	V3	High output
**L.A.1**	Normal	Qubit	4 µg	Covaris	400-550	2% Agarose Gel	KapaBio	beads/std KapaBio	no	17 pM	NA	MiSeq	V2	2x250
**L.A.1**	Tumor	Qubit	4 µg	Covaris	400-550	2% Agarose Gel	KapaBio	beads/std KapaBio	no	17 pM	NA	MiSeq	V2	2x250
**L.B**	Illumina	Qubit	1 µg	Covaris	400	E-gel	TrueSeq DNA		10			HiSeq 2000	V3	High output
**L.C**		Qubit	2.5 µg	Covaris	~500	2% Agarose Gel ~650 bp	NEBNext	Shearing/End Repair/Atail/Ligation(Gel):Zymo PCR Purification: 1.8X Ampure Additional Clean Up to remove small fragments (post BA): 0.5X	12 Cycles Post Ligation Clean Up	17 pM	cBot	HiSeq 2500 HiSeq 2000	V1 (RR) V3 (HT)	Both Rapid/High Output
**L.D**	Illumina	PicoGreen	1 µg	Covaris	500-600	Agarose Gel 500-600bp cut	TrueSeq DNA		10	12:00 pm	cBot	HiSeq 2000	V3	High output
**L.E**	Tumour: NEB	Qubit	2.8ug	Covaris	520-720	1.5% Agarose Gel (Pippin)	NEBNext	None extra	No	13.6 pM	cBot	HiSeq2000	V3	High Output (2x100bp)
**L.E**	Normal: NEB	Qubit	2.8ug	Covaris	520-720	1.5% Agarose Gel (Pippin)	NEBNext	None extra	No	8 pM	cBot	HiSeq2000	V3	High Output (2x100bp)
**L.F**	NEB	Qubit	1 µg	Covaris	400	AMPureXP Beads	NEBDNA		10			HiSeq 2000	V3	High output
**L.G**	PCR-FREE	Qubit	1 µg	Covaris	350	AMPureXP Beads	TrueSeq DNA PCR-Free		0			HiSeq 2000	V3	High output
**L.H**	SURE-SELECT	Qubit	503 ng tumor, 318 ng control	Covaris	175	AMPureXP Beads	SureSelect WGS		6+4			HiSeq 2500	V3	High output

Supplementary Table 1. Library construction and sequencing methods.

Cont	Max	30x	% at 30x	50x	% at 50x	100x	% at 100x	150x	% at 150x	200x	% at 200x	250x	% at 250x
**0**	1130	870	77 (77)	968	86 (86)	1068	95 (95)	1079	95 (95)	1086	96 (96)	1081	96 (96)
**17**	1105 (1130)	802	73 (71)	916	83 (81)	1015	92 (90)	1049	95 (93)	1043	94 (92)	1040	94 (92)
**33**	1064 (1130)	718	67 (64)	857	81 (76)	959	90 (85)	987	93 (87)	997	94 (88)	988	93 (87)
**50**	1058 (1130)	590	56 (52)	787	74 (70)	905	86 (80)	931	88 (82)	954	90 (84)	934	88 (83)

Supplementary Table 2. Number and percentages of SSMs found at different coverage and contamination levels. Values in parentheses are percentage of the best possible combination (0% contamination and 250x coverage).

set	AFThi (>0.5)	AFNhi (>0.5)	sameAF	DPNhi	DPNlo	centr	inTR	adjTR	nestRep	msat	dups	sRep	DukeBL	DacBL	hp	AFTlo(<0.1)	mult100	mult150
**GOLD**	198 (16%)	0 (0%)	74 (6%)	10 (0.8%)	2 (0.2%)	18 (1.5%)	112 (9%)	55 (4.4%)	397 (32%)	0 (0%)	42 (3.3%)	34 (2.7%)	6 (0.5%)	6 (0.5%)	52 (4.1%)	341 (27%)	12 (1%)	3 (0.2%)
**MB.A**	172/26/3	0/0/0	13/61/92	4/6/13	0/2/3	4/14/1	35/77/27	9/46/18	234/163/41	0/0/0	17/25/59	0/34/1	1/5/0	1/5/0	2/50/0	21/320/86	7/5/32	2/1/16
**MB.B**	174/24/1	0/0/0	23/51/4	6/4/2	1/1/1	13/5/0	58/54/8	34/21/1	246/151/3	0/0/0	18/24/3	16/18/8	6/0/0	6/0/0	28/24/0	37/304/3	1/11/0	0/3/0
**MB.C**	173/25/9	0/0/0	24/50/865	5/5/237	2/0/13	14/4/454	60/52/249	26/29/17	239/158/457	0/0/0	16/26/393	19/15/520	6/0/436	6/0/474	26/26/11	31/310/904	5/7/155	0/3/80
**MB.D**	166/32/8	0/0/4	24/50/817	5/5/138	2/0/27	12/6/161	59/53/218	30/25/22	232/165/444	0/0/0	19/23/514	16/18/229	3/3/54	3/3/78	28/24/19	31/310/792	10/2/220	3/0/103
**MB.E**	170/28/10	0/0/1	12/62/117	7/3/42	2/0/24	11/7/12	43/69/103	11/44/14	233/164/81	0/0/3	26/16/101	13/21/93	5/1/2	5/1/5	4/48/5	19/322/92	10/2/69	1/2/36
**MB.F**	181/17/3	0/0/0	19/55/102	8/2/39	2/0/21	15/3/5	58/54/106	15/40/13	274/123/51	0/0/2	30/12/52	21/13/95	6/0/0	6/0/0	4/48/0	63/278/110	2/10/14	0/3/6
**MB.G**	189/9/4	0/0/0	28/46/81	6/4/36	0/2/3	9/9/4	66/46/38	25/30/6	282/115/57	0/0/0	26/16/75	11/23/33	1/5/8	1/5/11	27/25/8	68/273/86	10/2/69	2/1/57
**MB.H**	185/13/16	0/0/2	33/41/4771	6/4/1659	2/0/120	13/5/991	81/31/1060	33/22/111	295/102/2478	0/0/7	28/14/4108	24/10/1076	6/0/284	6/0/501	33/19/106	103/238/5242	10/2/3795	3/0/2357
**MB.I**	184/14/4	0/0/2	34/40/76	6/4/15	2/0/7	9/9/2	73/39/86	37/18/11	271/126/32	0/0/0	13/29/16	14/20/62	0/6/0	0/6/0	32/20/6	57/284/80	2/10/0	0/3/0
**MB.J**	193/5/8	0/0/3	30/44/30	9/1/12	2/0/12	14/4/5	73/39/29	35/20/10	268/129/15	0/0/2	27/15/18	20/14/28	6/0/1	6/0/2	35/17/0	45/296/26	6/6/9	2/1/4
**MB.K**	187/11/66	0/0/38	34/40/7176	10/0/221	2/0/884	15/3/69	83/29/5721	40/15/1531	297/100/2651	0/0/256	30/12/402	23/11/3616	6/0/37	6/0/38	37/15/1636	97/244/7419	7/5/85	1/2/36
**MB.L1**	71/127/0	0/0/0	15/59/1	0/10/0	0/2/0	4/14/0	28/84/1	18/37/0	15/382/0	0/0/0	0/42/0	0/34/0	0/6/0	0/6/0	15/37/1	32/309/2	0/12/0	0/3/0
**MB.L2**	183/15/6	0/0/0	32/42/163	5/5/92	1/1/2	15/3/45	78/34/67	36/19/11	279/118/82	0/0/1	26/16/157	20/14/72	6/0/11	6/0/18	33/19/8	81/260/131	2/10/46	0/3/17
**MB.M**	185/13/23	0/0/13	36/38/1239	9/1/153	2/0/109	15/3/62	85/27/895	37/18/251	295/102/635	0/0/63	27/15/438	26/8/705	6/0/12	6/0/22	35/17/166	77/264/1338	11/1/389	3/0/224
**MB.N**	177/21/3	0/0/0	27/47/209	5/5/75	2/0/19	9/9/21	69/43/127	24/31/21	268/129/100	0/0/4	25/17/141	14/20/113	0/6/0	0/6/0	25/27/11	69/272/251	6/6/61	3/0/27
**MB.O**	187/11/1	0/0/0	32/42/166	9/1/82	0/2/0	9/9/29	61/51/50	31/24/36	295/102/81	0/0/0	31/11/169	0/34/0	0/6/3	0/6/12	30/22/26	102/239/212	8/4/82	2/1/58
**MB.P**	190/8/3	0/0/0	22/52/181	8/2/9	2/0/4	12/6/7	69/43/200	23/32/41	259/138/88	0/0/7	25/17/42	20/14/146	5/1/1	5/1/2	29/23/10	33/308/173	9/3/12	1/2/5
**MB.Q**	180/18/0	0/0/0	25/49/12	4/6/10	2/0/1	13/5/2	63/49/16	30/25/2	264/133/5	0/0/0	21/21/11	19/15/10	6/0/0	6/0/0	30/22/2	50/291/14	2/10/3	0/3/1

Supplementary Table 3. True positive, false negative and false positive SSM calls (TP/FN/FP) on the medulloblastoma tumor-normal pair are shown for each analyzed feature. Only true positives are shown for Gold Set.

set	inTR	hp	nestRep	msat	dups	sRep	DukeBL	DacBL	mult100	mutl150	centr
**GOLD**	279 (83%)	240 (71%)	17 (5%)	0 (0%)	2 (0.6%)	9 (2.7%)	2 (0.6%)	2 (0.6%)	0 (0%)	0 (0%)	14 (4.1%)
**MB.A**	2/277/16	1/239/3	2/15/6	0/0/0	0/2/1	0/9/2	0/2/0	0/2/0	0/0/0	0/0/0	0/14/0
**MB.B**	131/148/20	126/114/17	10/7/0	0/0/0	0/2/0	5/4/1	2/0/0	2/0/0	0/0/0	0/0/0	7/7/0
**MB.C**	72/207/19	69/171/12	6/11/2	0/0/0	0/2/2	3/6/2	0/2/0	0/2/0	0/0/0	0/0/0	3/11/0
**MB.D**	2/277/6	2/238/5	5/12/7	0/0/0	0/2/10	0/9/3	0/2/0	0/2/0	0/0/5	0/0/4	1/13/0
**MB.F**	101/178/14	94/146/4	12/5/2	0/0/0	0/2/0	8/1/3	2/0/0	2/0/0	0/0/0	0/0/0	8/6/0
**MB.G**	138/141/47	134/106/30	13/4/1	0/0/0	0/2/2	5/4/2	2/0/0	2/0/0	0/0/13	0/0/12	10/4/7
**MB.H**	34/245/211	31/209/46	1/16/20	0/0/30	0/2/16	0/9/82	0/2/0	0/2/0	0/0/6	0/0/7	0/14/2
**MB.I**	219/60/181	211/29/96	15/2/28	0/0/7	2/0/20	6/3/40	2/0/0	2/0/0	0/0/0	0/0/0	13/1/6
**MB.J**	41/238/11	46/194/4	10/7/3	0/0/0	0/2/1	2/7/0	2/0/0	2/0/0	0/0/0	0/0/0	6/8/0
**MB.K**	219/60/408	209/31/290	15/2/57	0/0/6	2/0/23	7/2/73	2/0/3	2/0/5	0/0/12	0/0/12	11/3/10
**MB.L1**	47/232/1	48/192/1	1/16/1	0/0/0	0/2/0	0/9/0	0/2/0	0/2/0	0/0/0	0/0/0	0/14/0
**MB.L2**	92/187/10	88/152/5	9/8/5	0/0/0	0/2/1	3/6/4	2/0/0	2/0/0	0/0/1	0/0/1	8/6/0
**MB.N**	84/195/17	82/158/6	11/6/3	0/0/0	0/2/3	6/3/6	0/2/0	0/2/0	0/0/1	0/0/1	3/11/2
**MB.O**	103/176/39	100/140/24	8/9/8	0/0/1	0/2/4	4/5/12	0/2/0	0/2/0	0/0/8	0/0/8	2/12/0
**MB.P**	21/258/51	6/234/1	7/10/10	0/0/1	0/2/1	4/5/19	2/0/1	2/0/1	0/0/0	0/0/0	3/11/1
**MB.Q**	53/226/41	56/184/12	9/8/6	0/0/0	0/2/6	1/8/11	0/2/0	0/2/0	0/0/3	0/0/2	3/11/3

Supplementary Table 4. True positive, false negative and false positive SIM calls (TP/FN/FP) on the medulloblastoma tumor-normal pair are shown for each analyzed feature. Only true positives are shown for Gold Set.

PCT1	MuTect				Strelka			
	Somatic calls	Overlap with T3*	Sensitivity	Precision	Somatic calls	Overlap with T3*	Sensitivity	Precision
BWA	2795	1016	0.81	0.36	1399	949	0.76	0.68
BWA-mem	4299	1020	0.81	0.24	1449	959	0.76	0.66
GEM	5320	1007	0.8	0.19	1494	886	0.71	0.59
Novoalign	7989	1011	0.81	0.13	1332	940	0.75	0.71
*set GOLD SSM T3 containing 1255 mutations								

Supplementary Table 5. Sizes of SSM call sets produced by MuTect and Strelka in combination with the tested mappers. Overlaps with the tier 3 SSM GOLD set are displayed together with sensitivity and precision ratios.

**Precision and sensitivity of MuTect – Strelka call-set overlaps when compared to the GOLD SSM sets**												
	GOLD SSM T1* (AF > 10 %)				GOLD SSM T2* (AF > 5 %)				GOLD SSM T3* (T2 + AF < 5 %)			
	Somatic calls	Overlap with T1	Sensitivity	Precision	Somatic calls	Overlap with T2	Sensitivity	Precision	Somatic calls	Overlap with T3	Sensitivity	Precision
BWA	1014	883	0.92	0.87	1014	926	0.84	0.91	1014	935	0.75	0.92
BWA-mem	1034	896	0.93	0.87	1034	937	0.85	0.91	1034	946	0.75	0.91
GEM	941	833	0.87	0.89	941	865	0.79	0.92	941	872	0.69	0.93
Novoalign	1037	883	0.92	0.85	1037	918	0.83	0.89	1037	926	0.74	0.89
*sets GOLD SSM T1, T2 and T3 contain 962, 1101 and 1255 mutations respectively												

Supplementary Table 6. Sizes of SSM call sets formed by MuTect+Strelka consensus for the individual tested mappers. Overlaps with Tier 1-3 SSM GOLD sets are displayed together with sensitivity and precision ratios.

Filter	Description
DistanceToAlignmentEndMedian	The median shortest distance of the variant position within the read to either aligned end is less than 10
DistanceToAlignmentEndMAD	The median absolute deviation of the shortest distance of the variant position within the read to either aligned end is less than 3
LowMapQual	The proportion of reads at the variant position with low mapping quality (less than 1) is greater than 10%
MapQualDiffMedian	The difference in the median mapping quality of variant reads (in the tumor) and reference reads (in the normal) is greater than 5
VariantMapQualMedian	The median mapping quality of variant reads is less than 40
VariantBaseQualMedian	The median base quality at the variant position of variant reads is less than 30
VariantAlleleCount	The number of variant-supporting reads in the tumor is less than 4
VariantAlleleCountControl	The number of variant-supporting reads in the normal is greater than 1
StrandBias	The strand bias for variant reads covering the variant position, i.e. the fraction of reads in either direction, is less than 0.02, unless the strand bias for all reads is also less than 0.02.
Repeat	The length of repetitive sequence adjacent to the variant position, where repeats can be 1-, 2-, 3-, or 4-mers, is 12 or more
SNVCluster50	The largest number of variant positions within any 50 base pair window surrounding, but excluding, the variant position is greater than 2; variant positions are those in which the number of alternate allele is supported by at least 2 reads and at least 5% of all reads covering that position.
SNVCluster100	The largest number of variant positions within any 100 base pair window surrounding, but excluding, the variant position is greater than 4; variant positions are those in which the number of alternate allele is supported by at least 2 reads and at least 5% of all reads covering that position.

Supplementary Table 7. Summary of refined SSM filters developed using the medulloblastoma GOLD set and Strelka calls on BWA-aligned sequence data. Filters were created using thresholds chosen by assessing kernel density plots for Strelka SSM calls on BWA alignments for the medulloblastoma dataset.

BM	Call set	SSM calls	TP calls	FP calls	Precision	Recall	Jaccard	F1
MB	Strelka	1337	951	386	0.76	0.74	0.58	0.73
	Strelka + filters	911	897	14	0.99	0.72	0.71	0.83
CLL	Strelka	1497	1065	432	0.71	0.81	0.61	0.76
	Strelka + filters	1045	980	65	0.94	0.75	0.71	0.83

Supplementary Table 8. Applying filters derived using the medulloblastoma GOLD set improves accuracy in calling for both for the MB (training) and CLL (test) datasets. The “Strelka” call set are SSM calls that pass Strelka’s built-in filters but have had no further filters applied; “Strelka + filters” are SSM calls that pass the GOLD set-tuned filters.

	**14**	**13**	**12**	**11**	**10**	**9**	**8**	**7**	**6**	**5**	**4**	**3**	**2**	**1**
**CLL.A**	149	218	175	117	86	82	54	41	25	12	8	7	9	109
**CLL.O**	149	216	172	118	89	73	56	47	40	37	32	29	33	177
**CLL.E**	149	210	163	102	74	71	49	40	36	20	17	6	4	12
**CLL.U**	149	218	181	128	102	100	76	76	71	65	78	77	173	500(1665)
**CLL.D1**	149	218	181	126	102	99	75	75	71	58	71	67	167	211
**CLL.C1**	149	175	121	80	43	32	23	23	15	8	7	1	2	171
**CLL.B**	149	202	161	101	64	59	22	19	11	8	13	3	5	8
**CLL.R**	149	211	158	89	65	51	29	29	19	8	5	3	6	405
**CLL.K**	149	205	152	93	71	56	28	23	23	29	21	23	46	500(2126)
**CLL.I**	149	218	181	124	85	71	39	36	28	22	15	13	10	15
**CLL.G**	149	211	148	89	67	56	43	34	28	19	15	10	9	3
**CLL.S**	149	208	146	71	58	46	38	21	17	13	10	2	18	285
**CLL.T**	149	113	64	49	25	22	15	9	4	3	4	1	1	8
**CLL.P**	149	211	169	121	89	82	61	66	56	33	44	31	69	52
**Total**	**149**	**218**	**181**	**128**	**102**	**100**	**76**	**77**	**74**	**67**	**85**	**91**	**276**	**2456**

Supplementary Table 9. CLL SSMs selected for verification. Column heading indicate concordance, i.e. the number of submissions that agree on mutation call. In the cases CLL.U and CLL.K, only up to 500 private SSMs were chosen randomly for verification. The total row indicates the total number of SSMs selected for verification at each level of agreement.

	Definition	CLL SSM	CLL SIM
**Class 1**	Mutant AF >= 0.10	1142	118
**Class 2**	0.05 <= Mutant AF < 0.10	96	
**Class 3**	Mutant AF < 0.05	74	
**Class 4**	Ambiguous alignment	7	16
**Class 5**	High or low depth	53	
**Tier 1**	Class 1	1142	118
**Tier 2**	Classes 1 and 2	1238	
**Tier 3**	Classes 1, 2 and 3	1312	
**Tier 4**	Classes 1, 2, 3 and 4	1319	134
**Tier 5**	Classes 1, 2, 3, 4 and 5	1372	

Supplementary Table 10. Classification of SSM and SIM Gold Set mutations for the CLL benchmark. Numbers of curated mutations falling in each class or tier are shown. Successive tiers represent cumulative addition of lower allele frequency mutations, followed by those supported by ambiguous alignments, and finally those with either too low or too high a depth. SIMs were not subjected to such fine classification, with calls only assigned to classes 1 and 4.

	**Aligner**	**SSM calling software**	**Num SSMs**	**SSM precision**	**SSM recall**	**SSM F1**	**SIM calling software**	**Num SIMs**	**SIM precision**	**SIM recall**	**SIM F1**
**CLL.GOLD**	BWA/GEM	curated	1313	1.00	1.00	1.00	curated	118	1.00	1.00	1.00
**CLL.A**	BWA	In-house tool	1092	0.85	0.71	**0.77**	in-house	63	0.49	0.23	0.31
**CLL.B**	BWA	samtools, Varscan	825	0.95	0.60	0.73	GATK, Varscan	88	0.63	0.44	**0.52**
**CLL.C1**	GEM	samtools, bcftools	850	0.76	0.50	0.60	samtools, bcftools	63	0.46	0.21	0.29
**CLL.C2**	GEM	samtools, bcftools	2193	0.46	0.77	0.58	samtools, bcftools	64	0.59	0.29	0.39
**CLL.D1**	none	SMuFin	1670	0.71	0.89	**0.79**	SMuFin	88	0.75	0.49	**0.59**
**CLL.D2**	none	SMuFin	1348	0.76	0.77	**0.76**	SMuFin	51	0.78	0.32	0.46
**CLL.E**	BWA	SomaticSniper	953	0.93	0.67	**0.78**	samtools, Pindel	128	0.43	0.45	0.44
**CLL.F**	BWA	Strelka	1208	0.84	0.77	**0.80**	Strelka	83	0.80	0.49	**0.61**
**CLL.G**	BWA	Caveman, Picnic NSG	881	0.95	0.64	**0.76**	pindel	85	0.58	0.40	0.47
**CLL.I**	BWA	samtools, bcftools	1006	0.94	0.72	**0.81**	samtools, bcftools	165	0.26	0.33	0.29
**CLL.K**	BWA	Atlas-SNP2, CARNAC	3045	0.27	0.61	0.37	BreakDancer, Pindel				
**CLL.L**	BWA	Mutect, Strelka	1140	0.90	0.78	**0.84**	Strelka	87	0.76	0.49	**0.60**
**CLL.N**	BWA	Strelka	1231	0.84	0.79	**0.81**	Strelka	18	0.56	0.07	0.12
**CLL.O**	BWA	MuTect	1268	0.83	0.80	**0.81**					
**CLL.P**	BWA	Sidrón	1233	0.84	0.79	**0.82**	bcftools, PolyFilter	135	0.46	0.48	0.47
**CLL.R**	BWA	samtools	1227	0.63	0.59	0.61	samtools	522	0.03	0.12	0.05
**CLL.S**	SNAP	BamBam	1082	0.72	0.59	0.65					
**CLL.T**	Novoalign	GATK, Varscan2	467	0.96	0.34	0.50	GATK, Varscan	113	0.76	0.68	**0.72**
**CLL.U**	BWA	Varscan, Strelka	3159	0.38	0.92	0.54	GATK, Pindel, Varscan, Strelka	4152	0.02	0.76	0.05

Supplementary Table 11. Summary of accuracy measures. Shown are the evaluation results with respect to the CLL Gold Set (Tier 3). Shown are the main software used and number of calls, precision, recall and F1 score.

set	hp	nestRep	msat	dups	sRep	DukeBL	DacBL	mult100	mult150	centr	AFThi (>0.5)	AFTlo (<0.1)	AFNhi (>0.5)	sameAF	inTR	adjTR
**GOLD**	28	402	0	27	24	4	3	15	5	10	83	165	0	1	94	27
**CLL.A**	4/24/1	285/117/50	0/0/0	11/16/30	0/24/0	0/4/0	0/3/2	8/7/13	1/4/6	2/8/2	52/31/13	29/136/103	0/0/0	0/1/68	45/49/35	14/13/22
**CLL.B**	12/16/0	229/173/15	0/0/0	6/21/4	10/14/5	1/3/0	1/2/0	4/11/1	1/4/0	3/7/2	48/35/11	30/135/10	0/0/0	0/1/6	47/47/10	15/12/2
**CLL.C1**	4/24/2	187/215/76	0/0/0	5/22/88	10/14/74	2/2/52	1/2/55	0/15/1	0/5/0	3/7/65	35/48/9	3/162/172	0/0/0	0/1/142	33/61/31	10/17/4
**CLL.C2**	22/6/24	315/87/495	0/0/0	14/13/502	20/4/455	4/0/326	3/0/350	10/5/190	2/3/74	6/4/344	48/35/41	10/155/969	0/0/1	0/1/875	71/23/280	26/1/35
**CLL.D1**	24/4/97	363/39/185	0/0/8	22/5/112	21/3/165	4/0/15	3/0/23	14/1/53	5/0/16	10/0/31	78/5/30	88/77/327	0/0/1	1/0/255	85/9/244	27/0/71
**CLL.D2**	22/6/11	319/83/112	0/0/0	20/7/133	21/3/87	4/0/16	3/0/15	13/2/49	5/0/20	8/2/27	59/24/25	7/158/152	0/0/0	1/0/144	73/21/95	23/4/17
**CLL.F**	8/20/3	309/93/64	0/0/5	15/12/32	19/5/72	4/0/1	3/0/3	9/6/13	1/4/3	8/2/3	60/23/16	79/86/109	0/0/0	1/0/72	65/29/83	13/14/16
**CLL.G**	19/9/0	256/146/24	0/0/0	14/13/9	9/15/2	1/3/0	0/3/0	9/6/8	3/2/2	1/9/1	52/31/14	14/151/9	0/0/0	0/1/6	49/45/2	19/8/1
**CLL.I**	21/7/3	254/148/18	0/0/0	8/19/5	7/17/9	0/4/0	0/3/0	0/15/0	0/5/0	2/8/2	57/26/14	53/112/26	0/0/0	0/1/12	66/28/16	19/8/2
**CLL.K**	18/10/298	250/152/783	0/0/83	15/12/85	12/12/1223	2/2/16	1/2/16	9/6/21	3/2/5	7/3/23	52/31/166	57/108/985	0/0/11	0/1/1301	60/34/1599	16/11/320
**CLL.L**	19/9/6	319/83/50	0/0/0	16/11/22	20/4/11	4/0/1	3/0/4	11/4/11	2/3/4	8/2/4	62/21/17	79/86/48	0/0/0	0/1/18	75/19/18	23/4/6
**CLL.N**	19/9/23	326/76/76	0/0/3	19/8/54	16/8/47	0/4/0	0/3/0	11/4/32	2/3/19	5/5/19	63/20/13	72/93/121	0/0/0	1/0/82	71/23/72	21/6/13
**CLL.O**	19/9/10	328/74/86	0/0/0	22/5/58	0/24/0	0/4/0	0/3/4	10/5/42	3/2/22	3/7/7	57/26/11	125/40/153	0/0/0	0/1/79	61/33/22	18/9/5
**CLL.P**	20/8/28	322/80/71	0/0/1	22/5/25	20/4/52	4/0/2	3/0/5	10/5/14	3/2/5	10/0/5	69/14/24	33/132/91	0/0/0	1/0/66	70/24/88	20/7/35
**CLL.R**	16/12/6	232/170/148	0/0/0	10/17/103	13/11/47	2/2/13	1/2/22	9/6/28	2/3/9	3/7/36	48/35/118	5/160/254	0/0/2	0/1/280	52/42/60	16/11/14
**CLL.S**	15/13/76	227/175/102	0/0/0	10/17/106	10/14/34	2/2/1	1/2/3	2/13/64	0/5/41	3/7/10	20/63/10	49/116/241	0/0/0	0/1/196	53/41/107	16/11/30
**CLL.T**	8/20/4	35/367/3	0/0/0	0/27/0	0/24/0	0/4/0	0/3/0	0/15/0	0/5/0	0/10/0	13/70/2	14/151/9	0/0/0	1/0/10	25/69/7	4/23/2
**CLL.U**	24/4/350	375/27/704	0/0/22	24/3/548	23/1/655	4/0/81	3/0/122	14/1/351	5/0/198	10/0/139	78/5/178	97/68/1142	0/0/9	1/0/1139	87/7/881	26/1/313

Supplementary Table 12. True positive, false negative and false positive SSM calls (TP/FN/FP) on the CLL tumor-normal pair are shown for each analyzed feature.

set	inTR	mult100	mult150	centr	hp	nestRep	msat	dups	sRep	DukeBL	DacBL
**GOLD**	62	2	0	1	54	29	0	5	8	1	0
**CLL.A**	3/59/11	0/2/0	0/0/0	0/1/0	1/53/4	1/28/6	0/0/0	0/5/0	0/8/1	0/1/0	0/0/0
**CLL.B**	19/43/28	0/2/0	0/0/0	0/1/0	17/37/8	7/22/7	0/0/3	0/5/0	0/8/7	0/1/0	0/0/0
**CLL.C1**	4/58/21	0/2/0	0/0/0	0/1/3	2/52/8	6/23/5	0/0/0	0/5/5	0/8/6	0/1/0	0/0/0
**CLL.C2**	4/58/16	0/2/2	0/0/2	0/1/2	2/52/5	8/21/6	0/0/0	0/5/1	0/8/0	0/1/0	0/0/0
**CLL.D1**	14/48/9	0/2/4	0/0/1	0/1/3	15/39/4	8/21/10	0/0/0	0/5/5	1/7/3	0/1/2	0/0/2
**CLL.D2**	7/55/7	0/2/1	0/0/0	0/1/1	6/48/2	8/21/4	0/0/0	1/4/0	1/7/1	0/1/0	0/0/0
**CLL.F**	15/47/11	0/2/0	0/0/0	0/1/0	15/39/5	8/21/7	0/0/1	0/5/0	2/6/3	0/1/0	0/0/0
**CLL.G**	13/49/12	0/2/2	0/0/1	0/1/0	11/43/2	8/21/7	0/0/0	1/4/1	1/7/3	0/1/0	0/0/0
**CLL.I**	11/51/115	0/2/0	0/0/0	0/1/1	3/51/7	5/24/30	0/0/8	0/5/0	4/4/28	0/1/0	0/0/0
**CLL.L**	16/46/13	0/2/0	0/0/0	0/1/2	15/39/7	9/20/9	0/0/0	0/5/1	2/6/3	0/1/2	0/0/2
**CLL.N**	5/57/5	0/2/0	0/0/0	0/1/0	6/48/3	0/29/1	0/0/0	0/5/0	0/8/0	0/1/0	0/0/0
**CLL.P**	8/54/50	0/2/3	0/0/2	0/1/0	3/51/19	11/18/20	0/0/4	1/4/5	3/5/20	0/1/0	0/0/0
**CLL.R**	2/60/431	0/2/3	0/0/1	0/1/4	0/54/136	2/27/70	0/0/48	0/5/11	1/7/115	0/1/1	0/0/1
**CLL.T**	41/21/25	0/2/0	0/0/0	1/0/1	42/12/22	26/3/13	0/0/0	1/4/1	2/6/2	1/0/0	0/0/0
**CLL.U**	39/23/3650	0/2/94	0/0/50	1/0/67	34/20/611	16/13/970	0/0/185	1/4/173	5/3/1874	1/0/18	0/0/23

Supplementary Table 13. True positive, false negative and false positive SIM calls (TP/FN/FP) on the CLL tumor-normal pair are shown for each analyzed feature. Only TP are shown for Gold Set.

Library	Std dev coverage	Std dev |coverage tumor - coverage control|
**L.A control**	8.612266	5.13933
**L.A tumor**	8.578797	
**L.B control**	10.24628	5.385501
**L.B tumor**	10.07358	
**L.C control**	32.29665	10.58849
**L.C tumor**	38.49272	
**L.D control**	10.93364	5.26062
**L.D tumor**	11.15144	
**L.E control**	14.605	7.781308
**L.E tumor**	9.040918	
**L.F control**	26.88905	11.37083
**L.F tumor**	36.19055	

Supplementary Table 14. Evenness of coverage for each medulloblastoma sequencing library as measured by standard deviation of the coverage.

Feature	Feature length	Range Ross et al. (human)	Range current study	L.A control	L.A tumor	L.B control	L.B tumor	L.C control	L.C tumor	L.D control	L.D tumor	L.E control	L.E tumor	L.F control	L.F tumor
**AT_15**	788710	0.25-0.26	0.10-0.69	0.61	0.58	0.32	0.30	0.16	0.15	0.59	0.57	0.57	0.69	0.12	0.10
**GC_10**	3842519	0.39-0.43	0.10-0.84	0.79	0.76	0.38	0.45	0.15	0.13	0.84	0.83	0.47	0.84	0.13	0.10
**GC_75**	9889452	0.83-0.97	0.26-4.00	0.86	0.85	0.52	0.55	0.99	1.00	0.42	0.43	0.26	0.76	3.44	4.00
**GC_85**	1449541	0.37-0.62	0.20-3.74	0.82	0.82	0.20	0.22	0.43	0.45	0.24	0.24	0.20	0.74	3.20	3.74
**GC_80**	119251	0.49-0.96	0.18-1.20	0.96	1.20	0.25	0.23	0.39	0.44	0.18	0.19	0.71	0.59	0.94	1.06

Supplementary Table 15. Bias as measured by relative coverage in regions of biased nucleotide composition. Features are defined as in Ross et al. (Genome Biol. 14, R51 2013). AT_15, window of 130 bp with the center 30 bp being repeats of AT. GC_10, region of 200 bp with the center 100 bp having less or equal than 10% GC content. GC_75, window of 200 bp with the center 100bp having at least 75% GC content. GC_85, window of 200 bp with the center 100 bp having at least 85% GC content. GC_80, window of 130 bp with the center 30 bp being either >= 80% G or >= 80% C. Values in the table are given as relative coverage defined as follows: $Relative\ coverage\ = \frac{coverage\ of\ a\ given\ reference\ base\ in\ a\ genome}{mean\ coverage\ of\ all\ reference\ bases}$.

	Reads (control + tumor)			Minutes
	Total	Aligned	Uniquely aligned	CPU time
b37+d	2152793590 (100.0 %)	2112982704 (98.15 %)	2018366589 (93.76 %)	79348.8 (100.0 %)
b37	2152793590 (100.0 %)	2071267988 (96.21 %)	1996504352 (92.74 %)	92517.7 (116.6 %)
hg19r	2152793590 (100.0 %)	2058694644 (95.63 %)	1990780196 (92.47 %)	95894.3 (120.85 %)

Supplementary Table 16. Comparisons of reference genome build versions on alignment rates and alignment times. (All mapping was performed with Novoalign2.) Using a larger reference genome build leads to higher mapping rates and shorter mapping times.

SAMPLE	TYPE	JOBS	WALL(h)	CPU(h)	DISK(GB)
**PD12336a**	BRASS	41	17	169.79	0.27
**PD12336a**	BWA	111	239.2	738.69	0
**PD12336a**	CAVEMAN	3293	3070.41	3196.16	24.38
**PD12336a**	EXT_SEQ	0	0	0	113.72
**PD12336a**	GBROWSE	1	3.5	4.12	0
**PD12336a**	NGSCN	29	16.86	16.25	0
**PD12336a**	PINDEL	158	227.83	224.14	6.16
**PD12336a**	VCF	3	10.42	7.87	0.15
**PD12336b**	BRASS	38	21.17	210.2	0.26
**PD12336b**	BWA	115	278.27	884.4	0
**PD12336b**	EXT_SEQ	0	0	0	133.86
**PD12336b**	GBROWSE	1	4.48	5.28	0
**PD12336b**	NGSCN	27	20.22	19.38	0
		**3817**	**3909**	**5476**	**278.8**

Supplementary Table 17. CLL.G Computational resources.

Step	Lane1	Lane2
**bwa aln**	2h15	1h20
**bwa sampe**	4h45	1h40
**sort/index bam**	1h30	1h10
**realign**	1h05	50min
**markDup**	1h05	1h
**recal1**	16h10	14h
**recal2**	4h50	3h20
	**Tumor**	**Normal**
**merge**	10h	11h40
**rmDup**	11h20	13h20

Supplementary Table 18. CLL.O Computational resources.

Supplementary Note 1

The ICGC Verification group was tasked with providing guidelines for sequencing and somatic mutation calling. We first carried out a mutation calling benchmark exercise on chronic lymphocytic leukemia (CLL), which was followed by a second exercise using a case of medulloblastoma (MB), in which we compared both sequencing methods and somatic mutation calling pipelines. The experience gained from the organization of CLL benchmark was used to improve the organization and methods of the MB benchmark. Here we present the entire CLL benchmark methods and results as well as additional results of the MB benchmark analysis.

Chronic Lymphocytic Leukemia Benchmark

CLL Whole genome sequencing

Whole genome sequencing reads were produced using DNA from a patient suffering from CLL who had given informed consent for sample collection and analysis. Tumor samples were from before treatment and tumor cells were separated from non-tumor cells by immunomagnetic depletion of T cells, natural killer cells, monocytes and granulocytes[2]. Tumor cell purity was >98% as assessed by flow cytometry. Normal blood cells from the same patient were used for the normal sample that contained less than 0.05% tumor cell contamination as assessed by flow cytometry. The tumor sample was sorted using FACS CD. Two different libraries each for the tumor and the corresponding normal DNA sample using Illumina TruSeq™ DNA Sample Preparation procedures with slight modifications. While one library was prepared following the standard protocol with 10 cycles of PCR, the second library was heated to 72 degrees Celsius before adapter ligation and cooled down suddenly to 4 degrees Celsius. This resulted in a biased proportion of high GC content reads and counterbalances some of Illumina's sample preparation methods' GC-bias (improved coverage of elevated GC-content regions of the genome. The normal and GC enriched libraries were sequenced on Illumina GAIIx (2x150 bp) and Illumina HiSeq2000 (2x100 bp) instruments. The same amount of data (~40x coverage) was produced for tumor and corresponding normal sample with the proportions of the two library types - 600 million reads for the standard and 200 million reads for the GC enriched library. Reads in FASTQ format were generated using the RTA software provided by Illumina.

Submission of CLL mutation calls

Illumina sequence corresponding to 40x coverage of a CLL tumor sample and the corresponding normal sample (above) were made available to members of the ICGC. Participants were asked to return VCF v4.1 files for simple somatic mutations (SSM) and somatic indel mutations SIM), as well as a list of structural variations. We specified that submitters should classify calls that they had high confidence in and calls that might be present, but in which they had less confidence. The threshold for high and medium confidence was left up to the participants. We received 19 SSM and 16 SIM submissions, of which several were independent submissions by the same group using different versions of their pipelines (*e.g.* CLL.C1 and CLL.C2, CLL.D1 and CLL.D2) or a pipeline used for the MB benchmark (see below) that was run later on the CLL benchmark data (CLL.F, CLL.L and CLL.N, for example).

Verification by target capture and orthogonal sequencing technologies

Verification of mutation calls and generation of a "Gold Set" was carried out after the first 14 submissions. 4080 SSMs and 883 SIMs were selected for Haloplex target capture (**Supplementary Table 9**). This corresponded to all mutation calls shared by at least two submissions and up to 400 randomly selected

private calls from each submission. Haloplex target design and capture were performed independently by two different groups, using different sequencing platforms: MiSeq and IonTorrent.

For verification with MiSeq, Haloplex custom reagent was designed for the Illumina HiSeq platform through the Agilent SureDesign portal (https://earray.chem.agilent.com/suredesign). The input contained 4,674 target segments encompassing 192,069bp. Design parameters included an optimal amplicon length of <150bp and each selected variant was padded by 25 bases both 5' and 3' to be contained within the assayed segment. Target enrichment was designed from human reference genome hg19 (GRC37, February 2009). The designed Haloplex custom reagent contained 19,392 amplicons encompassing 170,430bp covering 88.73% of the input target. Samples were processed using 200ng of input DNA following the manufacturer provided Haloplex protocol. Each sample was sequenced using the Illumina MiSeq platform with 2 x 150 base reads. 3.3 and 5.0 million reads were obtained covering the Haloplex products for the reference and tumor samples, respectively. Greater than 80% of the original 4,674 input targets were sequenced at over 20x coverage.

For verification of somatic mutations using IonTorrent, a HaloPlex custom capture was designed to enrich a total of 5179 mutations (4492 substitutions and 687 small indels) with a target region of 100 bp centered on the mutation position using the SureDesign software (Agilent). Sequencing was performed using two runs of the IonTorrent 418 chip, resulting in a total of more than 5 and 8 million reads for the tumor and normal samples, respectively.

Generation of a CLL Gold Set

All 40x reads (GAIIx and HiSeq2000 reads that were provided to the participants), MiSeq and IonTorrent verification reads were aligned with GEM (gem-mapper) and converted to BAM format. Alignments were filtered to retain only primary alignments with mapping quality >= 20. Duplicates were removed with Picard, indels realigned at 1000 genomes indel target locations, and indels were left-aligned using GATK. The pileups at SSM positions were extracted using samtools mpileup with base quality threshold >= 13. Read depth and base counts were extracted using a custom script. Mutant allele and normal counts were compared using in-house software snape-cmp-counts, which compares alternate and reference allele counts in tumor and normal, and then scored according to the probability that they are derived from different beta distributions. Mappabilities with 0, 1 and 2% mismatches were computed for the reference genome (h37d5). The average mappabilities in 100bp windows preceding each candidate mutation were stored as tracks for visualization in IGV. In addition, the segmental duplication annotation from the UCSC browser was loaded into IGV. Mutations were then classified as follows. Mutations with sufficient depth (>=20) and a snape score >=0.98, average mappability of one, and no overlap with segmental duplications were automatically classified in the Gold Set according to their mutant allele frequency (class 1: MAF>=0.1, class 2: 0.1>MAF>=0.05 or class 3: MAF<0.05). All other candidates with snape score >0.9 were reviewed visually in IGV. Mutations with ambiguous alignments were assigned to class 4. Low depth but otherwise plausible mutations were assigned to class 5. Somatic mutation Gold Set tiers were compiled by cumulative addition of classes so that Tier 1 only includes class 1, while Tier 2 includes class 1 and class 2, Tier 3 includes classes 1, 2 and 3, etc. All other candidate mutations were rejected and assigned to class 0 (**Supplementary Table 10**). The CLL Gold Set had a total of 1507 bona fide mutation calls across all, with 1142, 1238, 1312, and 1319 SSMs in Tiers 1, 2, 3 and 4, respectively, and 118 and 134 SIMs in Tiers 1 and 4, respectively. This corresponds to a mutational load of almost one verified mutation every two Mbp.

Initial assessment and revisions

Submitted mutation calls on the CLL benchmark data exhibited a low level of concordance (**Supplementary Figures 22-25**). In the initial submission we observed an alarmingly low degree of agreement: the number of CLL SSMs called in any one call set ranged from 250 to 18000 in the first 14 submissions prior to correction, and after correction ranged from 500 to 15,000, while the number of SSMs that all call sets agreed on was initially only 77, increasing to 149 after correction. CLL.C2, CLL.K and CLL.U contributed a large number of private calls (see **Supplementary Fig. 23**) whereas several other submissions had hardly any private calls. For CLL SIMs, the number of calls ranged from 18 (CLL.N) to 4152 (CLL.U) (**Supplementary Fig. 25**). It is clear that there is substantially less overlap among SIM call sets. The intersection of all SIM call sets resulted in only five SIMs. As the goal of the exercise was not to compete but to cooperate in order to improve the state of affairs, submitters were allowed to revise their submissions several times, each time using feedback on the level of concordance among all submitted call sets, but with no information about specific mutations. The overall results of the resubmissions and additional new submissions were not much improved, with the exception that submissions with very high numbers of SSM/SIM calls had revised call rates more in line with the rest of the pack.

Evaluation of CLL submissions with respect to Gold Set

Precision and recall of CLL SSM and SIM submissions are shown in **Supplementary Fig. 26** and tabulated in **Supplementary Table 11**. Clustering of TP or FP mutation calls based on Jaccard score (overlap) is shown in **Supplementary Figures 27-30**. Clustering of pipelines based on parameters is shown in **Supplementary Fig. 31** (input parameters are available in the **Supplementary Data File**). A rainfall plot of genomic positional clustering of calls is shown in Supplementary Figure 32. **Supplementary Figures 33-36** show enrichment or depletion of genomic and alignment features features in FP and FN CLL SSMs and SIMs. The absolute numbers for SSMs and SIMs are tabulated in **Supplementary Tables 12 and 13**, respectively.

Medulloblastoma Benchmark

Effect of library preparation and sequencing methods

A single tumor-blood DNA pair was sequenced at multiple sites (the National Center for Genome Analysis (CNAG), Barcelona, Spain; the German Cancer Research Center (DKFZ), Heidelberg, Germany; the RIKEN institute, Tokyo, Japan; the Ontario Institute for Cancer Research (OICR), Toronto, Canada and the Wellcome Trust Sanger Institute, Hinxton, UK). The results were subsequently compared using both local and centralized analysis. The tumor chosen for this analysis was a medulloblastoma (a malignant pediatric brain tumor arising in the cerebellum[3,4]) from the ICGC PedBrain Tumor project. This tumor type typically shows a very high tumor purity (usually >95%), but also often carries ploidy changes and other copy number alterations, thereby allowing for analysis of mutation detection performance at different allele frequencies[5]. Merging data from the different contributing centers and analyzing the combined dataset resulted in an extremely high WGS coverage of >300x for the tumor and >250x for the germline control. This allowed us to investigate variant-calling parameters at very low allele frequencies, as well as the impact of imbalanced tumor *vs.* control coverage levels and of total sequencing coverage on mutation detection performance.

Influence of library preparation on sequencing metrics

An even coverage is important for RNA and DNA sequencing[6]. The evenness of the coverage will be highly affected by structural events such as chromothripsis. Therefore, chromosome 22, being a small chromosome without copy number aberrations in this tumor (**Supplementary Figure 1**), was chosen to

further assess base-wise coverage. The standard deviation of coverage ranged from 8.58 in the most evenly covered library to 38.49 in the most unevenly covered, which could have a significant impact on the ability to call copy number variations (**Supplementary Table 14**). Differences in coverage between tumor and control also influence the ability to call simple somatic mutations (SSMs) and small insertions/deletions (somatic indel mutations, SIMs), so we additionally calculated the standard deviation of the absolute pairwise coverage difference (tumor vs. control). The values ranged from 5.14 in a good library to 11.37 in a strongly biased library (**Supplementary Table 14**). Two methods showed a marked variation in coverage, with a dramatic and unexpected increase in the number of sequencing reads mapping to regions of high GC content. This also resulted in much 'noisier' copy number profiles derived from these libraries, likely reducing the resolution at which structural variants could be reliably called (**Supplementary Figure 1**). One possible explanation for this may be DNA-binding beads used during the clean-up process, which could feasibly bind more strongly to GC-rich sequences at a given fragment size under certain concentration and/or temperature conditions.

While combining all libraries to give a coverage of close to 300x reduced the 'missing' exon fraction to just 0.1%, some regions of the genome (including part or all of ~80 genes) were still only covered at <=10x (**Supplementary Data 1;** none of these genes are listed in the Cancer Gene Census[7]). The vast majority of these regions (>98%) were in non-uniquely mappable areas such as telomeric or centromeric repeats. These will likely never be covered using routine short-read methods, regardless of the total read count (*e.g.* in stretches of long, highly homologous repeats). Library A also contained some longer 2x250bp MiSeq reads as opposed to standard HiSeq 2x101bp, but the overall contribution of these (below 2x) was too low to assess whether they may help in covering some of the missed regions.

We also examined the performance of each dataset in regions of biased nucleotide composition that were previously reported to be challenging to sequence across different platforms[8]. There was a marked variation in coverage in these regions, in keeping with the notable GC-bias observed in some libraries (**Supplementary Table 15**). The best overall performance in terms of evenness of coverage was seen with the PCR-free library, and this also outperformed the methods previously reported in the study of Ross *et al.*[8]. Of note, some regions showed a significant discrepancy in coverage between tumor and normal in certain regions, which would likely compromise variant calling in these loci.

Comparison of variant calling on the different libraries

The first comparison of variant calls that we performed was using each individual center's own mutation calling algorithm on their sequencing output, which resulted in a surprising amount of variation. Whilst there was a core set of mutations called by all 5 centers, this was the case for less than 20% of the total number of called variants (**Supplementary Figure 37a**). Allele frequency plots indicated that these consensus calls showed clear peaks at ~50% (heterozygous mutations occurring while the tumor was diploid) and ~25% (mutations occurring in 1 of 4 alleles after tetraploidization of the genome), while those made by less than 4 centers were shifted towards a lower allele frequency. This may indicate either increased variability in sensitivity of the pipelines as allele frequency decreases, and/or some mutations at such a low frequency that there were no variant reads in certain datasets (**Supplementary Figure 37a**). The mutation contexts of the variants were reasonably similar across centers, with the majority being C > T transitions in a GpCpG or ApCpG context, although some variability can clearly be seen across the 5 sets (**Supplementary Figure 37b**). Roughly one third of the mutations were unique to only one center, with the remainder variably called by 2-4 groups. One of the most notable differences was the low total number of calls made by center C, resulting in a large proportion of calls called by the other 4 centers but not this one. Based on the outcome of the ICGC benchmark analyses, however, this center has now modified its analysis pipeline to slightly relax some over-stringent filtering steps, resulting in a much greater overlap with the

other calls (not shown). When looking further at mutational signatures as defined by Alexandrov and colleagues[1] rather than simple base change contexts, variation can also be seen per center in the number and type of mutational processes identified (**Supplementary Figure 37c**).

In terms of coding alterations, there was a greater degree of overlap, but certainly not 100% concordance. Four non-synonymous, one splice-site (*SET*) and one stop gain (*ANGPT1*) SSMs were identified from the curated MB Gold Set of somatic mutations, which were also present at more than 10% allele frequency in each individual dataset. Of these, one center called all 6, two centers called 5, and one 4. One outlier called only two originally, which was found to be a result of minor contamination of the control sample with tumor DNA. A second library preparation resolved this issue, and all 6 SSMs were subsequently called (**Supplementary Figure 38**). One analysis pipeline also indicated a potential SSM in *ZMYM3* that was not detected in the other sets. Further inspection revealed that this alteration is probably a complex SIM rather than a single point change (discussed below).

Interestingly, the variation of calls between these five centers was higher for this exercise than for the somatic mutation calling pipeline benchmark. In particular, each center calling on their own library produced a higher variation than for the same centers calling on the same tumor-normal pair, but on data from only one center (L.A), clearly indicating that library variations contribute to the observed heterogeneity of mutation calls. When excluding unique calls, fewer than 60% of SSM calls were shared between four or more centers and fewer than 20% were called by all five when analyzing different libraries. When using only one library, however, more than 60% of SSM calls were shared across all centers (**Supplementary Figure 39**).

Although the previous comparison already provided some evidence of a role for pre-analysis sequencing pipelines in generating differences between datasets, we wanted to further assess this by removing any variation in the analysis pipeline itself. We therefore re-aligned and re-called mutations on each dataset using one standardized pipeline (the DKFZ pipeline was chosen for logistical reasons). This resulted in a notably better consensus of mutations called by more than one center (>80% called by at least 4 out of 5 centers, **Supplementary Fig. 40a**, versus <60% with different pipelines, **Supplementary Fig. 37a**), but an unexpected increase in the number of private mutations, particularly for one center. A shift towards lower allele frequencies was again seen in the mutations not called in all centers (**Supplementary Fig. 40a**). Analysis of mutation contexts indicated that the vast majority of these excess mutations were T>G transversions with low allele frequency, which were not observed at high frequency in the other datasets. Simply filtering out mutations with low allele frequency arising in this context resulted in an improvement in the overlap of mutation calls, but many more exclusively called ('private') alterations remained compared with the center's own calls on their data (filtered against other reference samples, **Supplementary Fig. 37a** and **Supplementary Fig. 40b**). Closer investigation revealed that the cause for this artifact was a center-specific method for adjusting base quality q-scores, whereby a calibrating PhiX library was spiked into each sequencing lane. Unfortunately, this actually led to an increase in the specific artifact detected in this comparison (**Supplementary Fig. 40c)**, and the center has subsequently reverted to default q-score metrics. The fact that the same phenomenon was not seen in the center's own calls on their data (**Supplementary Fig. 37**) is because it had already been identified, and a customized filter applied to account for it (removal of such changes also observed in a panel of 48 sequenced normal samples). This emphasizes that care must be taken when re-analyzing publicly available genome data from external centers, particularly when details on library preparation and center-specific customized 'blacklists' are often not known. This effect also had an impact on the mutational signatures identified, with a different distribution of processes observed in this mutation set than for each center calling their own variants

(**Supplementary Fig. 40d**), further suggesting that both library preparation and calling algorithms can strongly affect the ability to accurately detect such signature.

Effect of reference genome builds

In addition to testing the choice of mapper on SSM calling, we investigated the effect of genome reference build version on SSM calling. Novoalign2 (http://www.novocraft.com/documentation/novoalign-2/) was used for mapping against the selected three human genome reference builds ("hg19r" being a subset of "b37", which in turn is a subset of "b37+decoy"). **Supplementary Table 16** shows that using a larger reference build leads to higher mapping rates and shorter mapping times. Rather than benefiting from the additional alignments (alignments to likely uninteresting parts of the extended reference), the advantage of the decoy sequences should be in prevention of misalignments (i.e. avoiding incorrect mapping to chromosomal regions by providing the appropriate reference). Conducted SSM calling tests showed that smaller reference builds lead to significantly larger unfiltered SSM call sets (removal of decoy sequences from the reference accounts for 25 – 31 % increase in the SSM call set size in our experiment, depending on the caller (**Supplementary Fig. 41**). The fraction of these additional calls that overlap the Gold Set is however negligible (0.00).

Effect of mapper

Qprofiler results indicate that relative mapping rates for the individual chromosomes are generally similar for all the mappers, with minor exceptions for chromosome Y and the decoy contig (**Supplementary Fig. 42**). Distributions of insert sizes are similarly in good overall agreement, especially in the ranges that are expected to contain the bulk of proper pairs (**Supplementary Fig. 43**).

Apparent inter-mapper differences appeared to be in the assignment of mapping quality values (**Supplementary Figures 44a and 44b**), with GEM tending to assign lower mapping qualities than the other mappers, while Novoalign2 tending to do the opposite. Whether distinct mapping quality assignments alone can impact SSM calling will depend on given SSM caller's methods for utilizing mapping quality information. One of the main reasons for an abnormally large SSM call set produced by MuTect on Novoalign2 alignments might be MuTect's sensitivity to Novoalign2's mapping quality assignments – a very low fraction of reads with mapping quality 0 together with a noticeably higher fraction of reads with mapping quality 20 (when compared to the other mappers, **Supplementary Fig. 44c**). Reads with mapping quality of 0, unlike reads with mapping qualities in the range between 1 and 4, are used by MuTect in a built-in mutation filter; mutation support by at least one read with mapping quality of 20 is required by the same filter.

Alignment-level insertion and deletion statistics (**Supplementary Fig. 45**) reveal that GEM alignments contain slightly fewer deletion events (of size at least 15 bp), while for Novoalign2 alignments a similar trend is evident for insertions and deletions with size of at least 35 bp. The latter phenomenon might be caused by Novoalign2 trimming its input reads to 150 bp in length by default.

Perhaps the most prominent differences can be observed in MD-field derived alignment mismatch statistics. When mismatch counts are considered (**Supplementary Fig. 46**), consistent differences between the mappers can be observed – for all non-ambiguous mismatch types, the counts are highest for GEM alignments, with BWA-mem, BWA and Novoalign2 following always in the same order. When ratios of the individual substitution types are considered as a measure instead of absolute counts (**Supplementary Fig. 47**), two additional disproportions become more clearly visible. Firstly, some symmetrical mismatches are

well balanced (C>T and G>A) while others are not (C>A and G>T). Being largely mapper-independent, this bias might be originating upstream of the mapping step. Secondly, the individual mappers seem to have preferred mutations types (e.g. Novoalign2 alignments contain relatively high fractions of C>T and G>A mismatches and relatively low fraction of A>C mutations, while the situation is reversed for BWA-mem alignments).

Except for the suspicion that mapping quality score assignment strategy might considerably influence SSM calling in specific pipeline setups, no links have been established between the observed mapper differences and their causes (i.e. particularities of respective alignment methods) or effects (impacts on mutation calling). An additional study, wholly focused on establishing such links, could however be of great benefit for evaluation and development of custom pipeline designs.

It is important to note that performances of individual components and their combinations might vary depending on the experiment type, sequencing depth and analysis steps that have not been tested or included in our benchmarking.

Supplementary Methods

CLL Somatic Mutation Calling Pipeline Descriptions

CLL.A

FASTQ PROCESSING

(no details specified)

MAPPING AND BAM PROCESSING

Read sequences were mapped by BWA v0.5.8a to the human reference genome (GRCh37) with default settings. Possible PCR duplicated reads were removed by SAMtools[9] v0.1.8 with default settings. After filtering by pair mapping distance, mapping uniqueness and orientation between paired reads, the mapping result files were converted into pileup format by SAMtools with -scf option.
We used three kinds of read filters: set1; both read pairs were uniquely mapped with consistent orientation and pair distance (within average ± 3 s.d.), set2; at least one read pair was uniquely mapped with consistent orientation and pair distance and, set3; all uniquely mapped paired reads and set2, as described elsewhere.

MUTATION CALLING

SSM

SIM

SSM and SIM calls were done using all three sets of filtered reads, and mutations identified in the all three sets were considered as candidates.

The scripts for SSM and SIM calling are available from http://emu.src.riken.jp.

SV

Inconsistent read pairs which occurred within 500bp of each other were considered to support the same rearrangement. We identified candidate rearrangements in both tumor (support read pairs ≥ 4) and normal tissue (support read pairs ≥ 1) samples, and tumor specific rearrangement candidates were identified. To exclude mapping errors, we performed a blast search of read pairs that support rearrangements against the reference genome. If a paired read mapped with correct orientation and distance (≤ 500 bp) with an E-value < 10-7, we excluded that read pair. Reads mapped with more than two mismatches were also discarded. After filtering, candidates supported by ≥ 4 read pairs and at least one perfect match pair were considered as somatic rearrangements.

CNV

Copy number alternations were detected by calculating the ratio of the average depth of coverage in cancer to that in blood for 5kbp bins and analyzed the ratio using the DNAcopy R package.

MUTATION FILTRATION

SSM

- non-reference calls with a frequency ≥ 0.15 after removing bases calls with base quality < 10, and mapping quality < 20
- supported by at least two base calls including one base call with base quality ≥ 30
- a SAMtools consensus quality ≥ 20 and maximum mapping quality ≥ 40
- if three or more SNVs were found within any 10bp windows, or distance from nearest indel was less than 5bp, we discarded all SNVs

- if candidate non-coding SNVs were in a tandem repeat region suggested by tandem repeat finder, we discarded the SNVs
- if candidate SNVs were in RepeatMasker repeat regions (http://www.repeatmasker.org) within 1Mb from the centoromeric or telemeric gaps, we discarded the SNVs
- if a base with consensus quality lower than 20 occurs within 3bp on either side of the target SNV, we discarded the SNVs

After SNV calling in the tumor samples, candidate SSMs were filtered based on the lymphocyte sequence of the same patient:

- candidate SNV alleles with a frequency ≥ 0.03 after removing reads with base quality < 15, and mapping quality < 20
- depth of coverage in lymphocyte ≤ 7
- depth of coverage in lymphocyte ≤ 10 and candidate SSM allele was represented in the dbSNP database v131.

SIM

Short indels were identified based on gaps in a read's alignment by BWA. We defined indels using the following criteria:

- if indels were supported by a frequency ≥ 0.1 and ≥ 4 reads after removing reads with mapping quality < 20
- if candidate non-coding indels were in repeat regions suggested by tandem repeat finder or RepeatMasker, we discarded the indels.
- After indel calling in tumor samples, the candidate SIMs were filtered based on the lymphocyte sequence of the same patient using the following criteria;
- depth of coverage in lymphocyte ≤ 7
- for coding and non-coding region, if any indels were identified within 5bp or 10bp region in lymphocyte, respectively, the candidate SIM was discarded.

COMPUTATIONAL RESOURCES

(no details specified)

CLL.B

FASTQ PROCESSING

(no details specified)

MAPPING AND BAM PROCESSING

In the first step sequencing data were aligned to the human reference genome (GRCh37) using Burrows–Wheeler alignment (BWA) and sorted according to their chromosomal position. In the second step, read groups, library, sequencing platform and sample information were added to the initial alignment result BAM file Picard tools. In the third step, lane-level BAM files were aggregated into library-level BAM files, which at last were combined into sample-level BAM files.

Mismatch clusters in narrow regions are likely due to read misalignment and will lead to the accumulation of erroneously called mutations so we used the GATK to do the local realignment to solve this problem. In the final step, molecular duplicate reads from PCR amplification of library fragments are flagged to indicate artifacts.

After above processing the final BAM file was used to call SSM and SIM.

BWA version: 0.5.9-r16

bwa aln : -o 1 -i 15 -l 31 -k 2 -t 10 -m 100000 -e 63 -q 10 –I

bwa sampe using default parameter

Picard version: 1.54

We used the Picard AddOrReplaceReadGroups.jar module to add the reads group, library, sequencing platform and sample information in the BAM header, and the MarkDuplicates.jar module was used to flag the artifacts reads using default parameters.

GATK version: v1.0.6076

We used the RealignerTargetCreator and IndelRealigner module to select the regions where mismatches were clustered and to do the realignment process using default parameters.

MUTATION CALLING

SSM

SSMs were first predicted by Varscan[10].
SAMtools mpileup was run with parameters –Q 0 .
Varscan parameters were --min-coverage 10 --min-coverage-normal 10 --min-coverage-tumor 10 –min-var-freq 0.1 --min-freq-for-hom 0.75 --somatic-p-value 0.05 --min-avg-qual 0.

SIM

SIMs were first called using GATK SomaticIndelDetector using default parameters. For the SIMs and their flanking regions of 500bp, normal reads and tumor reads were realigned to hg19.
Then we uses Varscan to identify SIMs:
SAMtools mpileup was run with parameters –Q 0
Varscan was run with parameters --min-coverage 5 --min-coverage-normal 5 --min- coverage-tumor 5 --min-var-freq 0.1
Distance between adjacent SIM and to adjacent SSM had to be >10bp.

SV

We use seeksv which is inhouse software to call structural variations. It is similar to CREST and uses next-generation sequencing reads with partial alignments to a reference genome to directly map structural variations at the nucleotide level of resolution.
This pipeline has 5 steps:

- Get soft-clipped reads from the original normal alignment file and tumor alignment file.
- Aligned the clipped sequences (unmapped parts of the soft-clipped reads) to human reference genome.
- Get SV breakpoints basing on the two breakpoints' (soft-clipped reads) alignment positions.
- Compare the final tumor breakpoints to the soft-clipped reads file of normal sample and get the somatic SV breakpoints.
- Confirm the SV breakpoints with discordant read-pairs.

MUTATION FILTRATION

SSM

Filter parameters of high confidence results:

- Adjacent somatic mutation distance > 10
- The number of alternative allele reads >= 5
- Alternative allele frequency in the matched normal sample < 0.05
- Reads mapping to this mutations are not significantly enriched for mismatches (p-value>0.005)
- Reads with best hits greater than 1 are not significantly overrepresented at this position (P-value>0.01)
- Map Quality score not significant lower in mutated reads than other alleles (Map Quality score cutoff is 30. Fisher's exact test, p < 0.01)
- Base Quality score for mutated position not significantly lower than other alleles (Base Quality score cutoff is 20. Fisher's exact test, p < 0.05)
- Mutant allele frequency change between tumor and adjacent normal (Fisher's exact test p < 0.05)
- Mutations were not in gap aligned reads (in neither 20bp flank region less than 10 gap flags)
- Mutant allele not significantly enriched within 5 bps of 5' or 3' ends of reads (Fisher's exact test, p < 0.05)
- Mutations were not in simpleRepeatRegion (Repeat events less than 6) , simpleRepeatRegion (http://hgdownload.cse.ucsc.edu/goldenPath/hg19/database/simpleRepeat.txt.gz)

Filter parameters of medium confidence results:

- Adjacent somatic mutation distance > 10
- Map Quality score not significantly lower in mutated reads than other alleles (Map Quality score cutoff is 30. Fisher's exact test, p < 0.20)

- Base Quality score for mutated position not significantly lower than other alleles (Base Quality score cutoff is 20. Fisher's exact test, $p < 0.05$)
- Mutant allele frequency change between tumor and adjacent normal (Fisher's exact test $p < 0.05$)
- Mutations were not in gap aligned reads (in neither 20bp flank region less than 10 gap flag)
- Mutant allele not significantly enriched within 5 bps of 5' or 3' ends of reads (Fisher's exact test, $p < 0.05$)
- Mutations were not in simpleRepeatRegion (Repeat events less than 6) , simpleRepeatRegion (http://hgdownload.cse.ucsc.edu/goldenPath/hg 19/database/simpleRepeat.txt.gz)

SIM

Filter parameters of high confidence results:
- Indels were not annotated in the 1000 Genomes Project indels database
- The number of alternative allele reads >= 5
- Reads mapping to this mutations are not significantly enriched for mismatches (P-value>0.005)
- Reads with best hits greater than 1 are not significantly overrepresented at this position (P-value>0.01)
- Map Quality score not significantly lower in mutated reads than other alleles (Map Quality score cutoff is 30. Fisher's exact test, $p < 0.01$)

Filter parameters of medium confidence results:
- Indels were not annotated in the 1000 Genomes Project indels database

SV

Filter parameters of high confidence results :
- Deletions smaller than 100 base-pairs are filtered.
- When the left-breakpoint and the right-breakpoint of the tumor breakpoint are from the same chromosome. position of the left- breakpoint: p1 ,position of the right-breakpoint:p2, if $0 < p1 – p2 < 100$, this breakpoint is filtered.
- The mutation frequency of the tumor breakpoints must be at least 0.1.
- The minimum number of tumor soft-clipped reads is 3
- In normal sample, the number of soft-clipped reads must be 0.
- In the tumor sample, the number of discordant read-pairs that support the tumor breakpoint must be at least 1.
- In the normal sample, the number of discordant read-pairs that support the breakpoint must be 0.

Filter parameters of medium confidence results :
- Deletions smaller than 100 base-pairs are filtered.
- When left-breakpoint and right-breakpoint are from the same chromosome. position of left-breakpoint: p1 ,position of right- breakpoint:p2, if $0 < p1 – p2 < 100$, this breakpoint is filtered.
- The mutation frequency of the breakpoints must be at least 0.1.
- The minimum number of soft-clipped reads is 3.
- In the normal sample, the number of discordant read-pairs that support the breakpoint must be 0.
- In the normal sample, the number of soft-clipped reads must be 1 or 2 , and let the number of discordant read-pairs support the breakpoint in the tumor sample be N, let the number of soft-clipped reads support the breakpoint in the tumor sample be M, let the number of soft-clipped reads support the breakpoint in the normal sample be a, if $M+N-a >20$, put this result into medium confidence results.

COMPUTATIONAL RESOURCES

All analysis were done under CentOS v5.5 on a computer of x86_64 architecture with 32GB memory and 16 CPU. For each step computational resource consumption was as follows (based on one CPU and one process):
- BWA alignment: 1756 CPU hours max RAM:5G
- Picard AddOrReplaceReadGroups.jar module to add the reads group, library, sequencing platform in the lane level bam : 22 CPU hours max RAM:2G
- GATK realignment : 162 CPU hours max RAM:2G
- Picard mark duplication : 21 CPU hours max RAM 2G
- SAMtools index final BAM : 4 CPU hours max RAM 0.5G
- SSM detection: 32 CPU hours max RAM:4G
- SIM detection: 25 CPU hours max RAM:4G
- Somatic structural variation detection: 9 CPU hours max RAM:3G

CLL.C1

FASTQ PROCESSING

(no details specified)

MAPPING AND BAM PROCESSING

GEM

MUTATION CALLING

SSM

Samtools and Bcftools for Genotype identification

SIM

Samtools and Bcftools for Genotype identification

MUTATION FILTRATION

SSM

- Exclude positions with coverage <10 in any of both samples
- Exclude clustered SNV (window = 10, cluster size = 3)
- Exclude SNV close to Indel (window = 10)
- Exclude SNV with Strand Bias <= 0.001
- Exclude SNV in low Mappability regions according to GEM
- mappability file

Somatic identification High Confident mutations:

- Significance of allele frequency difference by Fisher Exact Test. P- value <= 0.01
- Genotype Quality >= 20
- Mutant allele frequency in control = 0
- Mutant allele frequency in case => 0.10
- Mutant allele bases in case => 4
- Exclude mutations in dbSNP with GMAF > 0.01

Somatic identification Low Confident mutations:

- Significance of allele frequency difference by Fisher Exact Test. P- value <= 0.1
- Genotype Quality >= 20
- Mutant allele frequency in control <= 0.02
- Mutant allele frequency in case => 0.10
- Mutant allele bases in case => 2

SIM

- Exclude positions with coverage <10 in any of both samples
- Exclude clustered indels (window = 10, cluster size = 3)
- Exclude indels with Strand Bias <= 0.001
- Exclude indels in low Mappability regions according to GEM mappability file

Somatic identification High Confident mutations:

- Significance of allele frequency difference by Fisher Exact Test. P- value <= 0.01
- Genotype Quality >= 20
- Muant allele frequency in control = 0
- Mutant allele frequency in case => 0.10
- Mutant allele bases in case => 4
- Exclude mutations in dbSNP with GMAF > 0.01

Somatic identification Low Confident mutations:
- Significance of allele frequency difference by Fisher Exact Test. P- value <= 0.1
- Genotype Quality >= 20
- Mutant allele frequency in control <= 0.02
- Mutant allele frequency in case => 0.10
- Mutant allele bases in case => 2

COMPUTATIONAL RESOURCES

(no details specified)

CLL.C2

FASTQ PROCESSING

(no details specified)

MAPPING AND BAM PROCESSING

Reference genome: hsapiens_v37.fa (GRCh37 downloaded in May 2010).

Each FLI separately:
starting from uniquely mapped, properly paired reads generated by the GEM mapping pipeline + selReads script (CNAG, Simon Heath)
sort by coordinates to make bam file compatible with GATK:
java -jar picard/1.73/ReorderSam.jar
add read groups:
java -jar picard/1.73/AddOrReplaceReadGroups.jar
reassign mapping quality to make bam file compatible with GATK:
SAMTOOLS/0.1.19/bin/samtools view -h readgroups.bam | awk 'BEGIN{OFS="\t"}{if($5 == 255){$5 = 35}; print $0}' | SAMTOOLS/0.1.19/bin/samtools view -Sb
index processed bam file:
SAMTOOLS/0.1.19/bin/samtools index

Taking together all FLIs of one library:
remove duplicates:
java -jar picard/1.73/MarkDuplicates.jar

MUTATION CALLING

Tools and versions:

- Picard version: 1.73
- Samtools and bcftools version : 0.1.19
- GATK version: 1.6.5
- snape-pileup (CNAG, Emanuele Raineri)

- snpEff[11] 2.0.5d
- vcfProcess (CNAG, Simon Heath)
- vcfPileupFT (CNAG, Francesc Castro/Sophia Derdak/Raul Tonda)
- vcf-somatic (CNAG, Francesc Castro)

SSM

Mutation calling per sample:
snape-pileup

Taking together all bam files for both samples:
# indel realignment:
java -jar GATK/1.6.5/GenomeAnalysisTK.jar -T RealignerTargetCreator --known GATK/bundle_1.5/hg19/1000G_phase1.indels.hg19.vcf --known GATK/bundle_1.5/hg19/Mills_and_1000G_gold_standard.indels.hg19.vcf
java -jar GATK/1.6.5/GenomeAnalysisTK.jar -T IndelRealigner
# mutation calling:
SAMTOOLS/0.1.19/bin/samtools mpileup -DSug -d 10000 realigned.bam | SAMTOOLS/0.1.19/bin/bcftools view -bvcg - | SAMTOOLS/0.1.19/bin/bcftools view -

SIM

Same as for SSMs (above)

MUTATION FILTRATION

SSM

mutation annotation:
java -jar SNPEFF/snpEff_2_0_5d/snpEff.jar eff GRCh37.65
soft filtering and joining samtools and snape results:
vcfProcess
Fisher test for allele frequencies at positions with difference in genotype between control and tumor:
vcfPileupFT
tagging of somatic candidates:
vcf-somatic, using the following parameters:
- Fisher Test p-value for the position <=0.01
- alternative allele frequency in the control sample <=0.05
- alternative allele frequency in the tumor sample >=0.10
- count of the alternative allele in the control sample <=1
- count of the alternative allele in the tumor sample >=2
- coverage in the control sample >=10
- coverage in the tumor sample >=10
- Genotype quality in the control sample >=20
- Genotype quality in the tumor sample >=20

Submitted somatic candidates:
- only positions with the somatic tag from vcf-somatic
- only positions with genotype 0/0 in the control and 0/1 in the tumor sample.

SIM

Same as for SSMs (above)

COMPUTATIONAL RESOURCES

(no details specified)

CLL.D

The SMUFIN[12] pipeline was used.

CLL.E

FASTQ PROCESSING

Data quality was assessed using FastQC and in-house contaminant/adapter screening tool.
The quality score distribution from FastQC was used to guide trimming of reads, particularly for the GAIIx 150bp paired end runs.

MAPPING AND BAM PROCESSING

Alignment:
BWA v0.5.9 was used to align the sequence reads in paired end mode (bwa aln and sampe) to the GRCh37/hg19 human reference genome from UCSC, with the following settings:
-I (to convert Phred+64 quality scores to Phred+33)

Merging lane data and marking duplicates:
Picard v1.66 was used to mark duplicate reads on a per-lane basis and then to merge BAM files for each sequencing lane into a per-sample BAM file (one for each of 184TD and 184ND) while also marking duplicates for the sample as a whole.

Post-alignment QC:
Further data QC was performed on the aligned BAM files to assess alignment and error rates, coverage, duplication, etc., both on a per-lane and per-sample basis, and to detect spatial artifacts, using tools developed in-house.

MUTATION CALLING

SSM

SomaticSniper[13] v1.0.0 was used to call SSMs with the following settings:
-q 1 -J -s 0.000001 -Q 0 -F vcf

SIM

Samtools and Pindel were used to call indels with the following settings:
Samtools v0.1.18
mpileup -uf
bcftools view -bvcg
varFilter -D1000
Pindel[14] v0.2.4d
Default parameters
The indel calls from samtools and Pindel were then combined.

SV

Large-scale structural rearrangements were detected using a combination of approaches including discordant read pair clustering, soft-clipped (or split) read analysis and de novo assembly to determine breakpoints to base pair resolution.

Discordant read pair analysis :
The analysis pipeline for identifying putative structural mutations (SVs) from paired end data comprises the following steps.

- Compute distribution of inferred insert sizes and extract discordant read pairs where each end maps to different chromosomes, or in incorrect orientation, or with an insert size greater than 5 standard deviations above the median
- Realign discordant read pairs and read pairs for which only one end was mapped using novoalign v2.07.15b using options -I (to specify the median insert size and standard deviation) and -r Random
- Extract discordant read pairs from resulting alignments using proper pair bit flag
- Cluster discordant read pairs from the tumor (184TD), matched normal (184ND) and a pool of 22 other non-related normal samples sequenced to 50x depth as part of the UK OAC ICGC project
- Soft-clipped read analysis
  - CREST v1.0[15] was used to analyze soft-clipped reads from the BWA-aligned BAM files in paired mode. Somatic SV calls were taken forward for assessment by de novo assembly as described below.BAM files for the tumor and normal were split on a per-chromosome basis, and the extractSClip perl script applied separately to each chromosome. The extracted soft-clipped reads were then concatenated together, and subtracted using the countDiff perl script before being passed to CREST.pl with the default parameters.

Local de novo assembly:
A local de novo assembly of reads in the vicinity of putative SV breakpoint ends was carried out to determine the breakpoint to base pair resolution and assess the validity of that breakpoint by finding breakpoint-spanning reads. This is carried using an in-house developed Java program that is under active development and that makes use of the SAM-JDK and Picard packages and calls a number of tools including Readjoiner v1.1, BLAT v34 and exonerate v2.2.

MUTATION FILTRATION

SSM

SSMs were filtered using the criteria below to produce a set of high and medium confidence call:

- Somatic mutations only (somatic status, SS = 2)
- False positive filters using fpfilter.pl script from SomaticSniper/VarScan2
- Average mutation position in supporting reads, relative to read length between 0.1 and 0.9
- Strandedness - fraction of supporting reads from the forward strand between 0.01 and 0.99
- Mutant read count >= 4
- Mutant read frequency >= 0.05
- Mismatch quality sum difference < 50
- Mutation mismatch quality sum < 100
- Mapping quality difference < 30
- Difference in average trimmed read length between reference and mutant reads < 25
- Distance to effect 3' end for mutant reads < 0.2
- Exclude mutations with adjacent homopolymer of at least 5bp
- Mutant reads in normal <= 1
- Mutant reads in tumor >= 2 (medium confidence if < 4)
- Exclude mutations within 40bp of indel in normal called using Pindel (see below)
- Check against Pindel calls for pool of oesophageal adenocarcinoma normals from UK OAC ICGC project, mutations within 40bp of an indel marked as medium confidence

SIM

- Consensus between mpileup and pindel
- Inclusion of only 0/0 genotype in the normal
- Checked against normal (Pindel call from normal sample), exclusion of anything within 40 bp of an INDEL in normal
- Checked against normals (PINDEL calls) from UK oesophageal adenocarcinoma ICGC project, exclusion of anything within 40 bp of an INDEL in a normal sample as medium confidence

SV

Apply somatic filter by accepting read pair clusters as follows:

- Supporting read pairs in tumor >= 4 and no supporting read pairs in any normal
- Supporting read pairs in tumor >= 7, no more than 1 supporting read pair in the matched normal
- Supporting read pairs in tumor >= 10, no more than 2 supporting read pairs in the matched normal
- Remove putative SVs in centromeric and telomeric regions and other known problematic regions that result in very high depth of coverage

COMPUTATIONAL RESOURCES

- Data QC and trimming: 30 CPU hours
- BWA alignment: 777 CPU hours
- Post-alignment processing (sorting, merging, marking duplicates) and QC: 104 CPU hours
- SNV calling and filtering: 6 CPU hours
- Indel calling and filtering: 228 CPU hours
- Realignment of discordant read pairs (novoalign): 600 CPU hours
- SV calling and filtering: 37 CPU hours

CLL.F

Same pipeline as MB.F (Supplemental Methods 3)

CLL.G

FASTQ PROCESSING

(no details specified)

MAPPING AND BAM PROCESSING

BWA: Default settings, appropriate insert size set for library
Sanity checking of SW mapped reads: If a read is SW mapped the soft clipping must pass the following criteria:

- leading edge of read must not be clipped by more than 5 bp
- trailing edge of read must not be clipped by more than (readlength*0.1)+1

MUTATION CALLING

SSM

Caveman[16], settings:
k = 0.32
rd = 500
y = 0.95
z = 0.8
copynumber segments generated by PICNIC[17] NGS

SIM

Pindel , settings:
Pipeline based on early version of v0.2 with various patches

SV

PICNIC NGS and Brass with default settings.

MUTATION FILTRATION

SSM

Post-processing on raw Caveman output:

- Mutation probability threshold: The mutant allele probability score based on the core algorithm was equal to or above 0.8
- Read depth: At least a third of bases in the tumor sample reporting the mutant allele had to exceed or equal a base quality of 25
- Average mapping quality: The mean mapping quality of reads reporting the mutant allele had to exceed 20
- Read Position: The mutant allele failed this flag if it was present in less than 8 reads AND only represented on the last third of a read or only last third and first 8% of any read
- Matched normal: The mutant allele failed this flag if it was present at base qualities exceeding 15 in more than 5% of reads in the matched normal sample
- Panel of other normals: The mutant allele failed this flag if it was present in at least 5% of reads in at least 2 samples from the panel of 50 randomly selected normal samples
- Pentameric motif: The mutant allele failed this flag if all reads carrying the mutation but one were unidirectional (on forward or reverse strands only) AND the mutations were only present in the last half of the read AND the reads carrying the mutant allele contained the motif GGC[A/T]G in the same sequencing direction as the mutation AND the mean base quality for every base after the mutation was calculated for each read and was less than 20
- Phasing: The mutant allele itself was required to have a mean mutant base quality of more than or equal to 21 and was not unidirectionally represented.
- Simple repeat: The mutant call was failed if it fell within a simple repeat or within the immediate 5bp flanking the boundaries of a simple repeat as defined by UCSC
- Centromeric microsatellite: The mutant call was failed if it fell within the boundaries of a centromeric repeat as defined by UCSC.
- HiSeq coverage: The mutant call was failed if it fell within a genomic window where the coverage in 2 or more genomes in a panel of normal genomes, exceeded 8 SD of the average of the coverage for those genomes or if it fell within parts of the genome which were consistently in the top 5% of coverage of HiSeq sequenced genomes as defined by UCSC.
- Germline indels: The mutant allele must not fall within the boundaries or be within ± 4bp of a germline indel as detected by the indel-detecting algorithm.

SIM

In -house post-processing methods for pindel output.

COMPUTATIONAL RESOURCES

See **Supplementary Table 17**.

CLL.I

FASTQ PROCESSING

(no details specified)

MAPPING AND BAM PROCESSING

BWA version 0.6.1-r104-tpx
bwa aln -t 8 -q 20
bwa sampe -t 8 -T -a 1000

MUTATION CALLING

SSM

samtools mpileup + bcftools implemented as part of cancer-genome-customized pipeline

MPILEUP_OPTS=" -E -R -q 30 -ug "
BCFTOOLS_OPTS="-vcgN -p 2.0 "
MPILEUPCONTROL_OPTS="-A -R -B -Q 0 -q 1"

SIM

same as for SSM, but read mapping quality of only >=20
MPILEUP_OPTS=" -E -R -q 20 -ug "

MUTATION FILTRATION

(no details specified)

COMPUTATIONAL RESOURCES

Primary analysis including alignments, merging of lanes, duplicate removal, coverage calculations:
CPU hours in total: 1400
cluster (PBS) with 924 cores on 51 nodes
walltime: ~41hours
For SSM and SIM calling:
CPU hours in total: 50
walltime: ~5hours

CLL.K

FASTQ PROCESSING

(no details specified)

MAPPING AND BAM PROCESSING

Mapping:
Burrows-Wheeler Aligner (BWA, v0.5.8)
bwa aln -t 8 [Number of threads] <in.db.fasta> <in.query.fq> > <out.sai>

Merging forward and reverse reads:
bwa sampe -P [Load the entire FM-index into memory to reduce disk operations] -r [Specify the read group] <in.db.fasta> <in1.sai> <in2.sai> <in1.fq> <in2.fq> > <out.sam>

We didn't quality trim reads.

GATK (a Toolkit for Genome Analysis, v1.6.0) was used to recalibrate and realign sequence reads before mutation analysis.

MUTATION CALLING

SSM

Single nucleotide mutations were called using Atlas-SNP2 (v1.3):
ruby Atlas-SNP2.rb -i [in.sorted.bam] -r [reference.fa] -o [output file] -n [sample name] -s [choosing platform: Illumina] –f 10000 [INT maximum number of alignments allowed to be piled up on a site]
SSM were selected using CARNAC (Consensus And Repeatable Novel Alterations in Cancer, v1.0).

SIM

Small insertions and deletions were called using Atlas-Indel2 (v0.3.1):
ruby Atlas-Indel2.rb -b [input_bam] -r [reference] -o [outfile] –s [The name of the sample to be listed in the output VCF file] –I [Illumina platform] -f [requires indels to have at least one mutant read in each strand direction] -P 0 [The indel probability (p) cutoff value for 1bp deletions] -p 0 [The indel probability (p) cutoff value for the logistic regression model]
SIM were selected using CARNAC (Consensus And Repeatable Novel Alterations in Cancer, v1.0).

SV

Structural variations were called using BreakDancer (v1.1) and Pindel (0.2.4t):
breakdancer_max <analysis_config_file> -y 50 [output score filter] -d [prefix of fastq files that SV supporting reads will be saved by library]

pindel -f [reference.fa] -i [bam_configuration_file] -c ALL [chromosome_name] -o [prefix_for_output_file] –b [use calls from BreakDancer to further increase sensitivity and specificity]

MUTATION FILTRATION

SSM

- Tumor mutation coverage >= 4
- Tumor mutation ratio >= 8%
- Normal mutation coverage < 4
- Ratio of ratios: normal mutation ratio < 1% of tumor mutation ratio
- Sum of normal mutation base qualities <= 70
- End-of-read filter: at least one mutant base must be in the center of a read
- Strand direction filter: at least one mutant base must appear in each direction.
- Max-mapping-quality filter: at least one mutant-supporting read must be uniquely mapped

SIM

- Tumor mutation coverage >= 10
- Tumor mutation ratio >= 15%
- Normal mutation coverage = 0

COMPUTATIONAL RESOURCES

(no details specified)

CLL.L

The Pipeline used to produce this data set is identical to the pipeline MB.L1 (Supplementary Methods 3) with the following exception (omitted step indicated with strikethrough text):
~~An in house script utilized tabix/bedfiles was used to identify loci withiin UCSC/hg19 SegDup, rmsk and SimpleRepeat regions and these were filtered out of the final output.~~
~~1. vcf-annotate.pl (in house script)~~

FASTQ PROCESSING

(no details specified)

MAPPING AND BAM PROCESSING

Alignment:
bwa 0.6.2, the final lane level bam is created by piping output through picard 1.90 AddOrReplaceGroups
bwa aln -t 8 ref.fa lane.R1.fq.gz > lane.R1.sai ; bwa aln -t 8 lane.R2.fq.gz > lane.R2.fq.gz

bwa sampe ref.fa lane.R1.sai lane.R2.sai lane.R1.fq.gz lane.R2.fq.gz | java -Xmx2g jar AddOrReplaceGroups.jar RGID.. VALIDATION_STRINGENCY=SILENT I=/dev/stdin O=lane.sorted.bam SO=coordinate CREATE_INDEX=true

Filter and Collapse. Aligned bams are filtered using samtools 0.1.19, then collapsed/deduplicated using picard 1.90:

samtools view -u -F 4 lane.sorted.bam | samtools view -u -F 256 - | samtools view -q 30 - > lane.sorted.filtered.bam
samtools index lane.sorted.filtered.bam
java -Xmx4g MarkDuplicates.jar I=lane.sorted.filtered.bam O=lane.sorted.filtered.collapsed.bam METRICS_FILE=lane.sorted.filtered.collapsed.bam.metrics ASSUME_SORTED=true REMOVE_DUPLICATES=true CREATE_INDEX=true

Merge. Lane level bams from the same sample are merged into a single file using picard 1.90:

java Xmx4g MergeSamFiles INPUT=lane1.sorted.filtered.collapsed.bam ... INPUT=laneN.sorted.filtered.collapsed.bam OUTPUT=sampleID.sorted.filtered.collapsed.merged.bam SORT_ORDER=coordinate

MUTATION CALLING

SSM

First Somatic Caller: Strelka
Mutation calls were made with strelka/v1.0.7, followed by mutation annotation using GATK 1.3.16.
Strelka config.ini contains the following values:

- isSkipDepthFilters = 0
- maxInputDepth = 10000
- depthFilterMultiple = 3.0
- snvMaxFilteredBasecallFrac = 0.4
- snvMaxSpanningDeletionFrac = 0.75
- indelMaxRefRepeat = 8
- indelMaxWindowFilteredBasecallFrac = 0.3
- indelMaxIntHpolLength = 14
- ssnvPrior = 0.000001
- sindelPrior = 0.000001
- ssnvNoise = 0.0000005
- sindelNoise = 0.000001
- ssnvNoiseStrandBiasFrac = 0.5
- minTier1Mapq = 20
- minTier2Mapq = 5
- ssnvQuality_LowerBound = 15
- sindelQuality_LowerBound = 30
- isWriteRealignedBam = 0
- binSize = 25000000

Strelka was run with arguments:

configureStrelkaWorkflow.pl --normal blood.sorted.filtered.collapsed.merged.bam --tumor tumor.sorted.filtered.collapsed.merged.bam --ref hg19_random.fa --config config.ini --output-dir analysis

Further steps:

cd analysis; qmake -inherit -- -j 16
java -Xmx4g -Djava.io.tmpdir=tmp -jar GenomeAnalysisTK.jar -T VariantAnnotator -R /hg19_random.fa --variant analysis/results/passed.somatic.snvs.vcf --dbsnp dbSNP137_chr.vcf -o passed.somatic.snvs.vcf

Second Somatic Caller: MuTect
Mutation calls were made with MuTect 1.1.4. MuTect was run in parallel, by chromosome, and the final output was merged with vcftools 0.1.7.

java -Xmx10g -Djava.io.tmpdir=tmp -jar MuTect-1.1.4.jar --analysis_type MuTect --reference_sequence hg19_random.fa --dbsnp dbSNP137_chr.vcf --cosmic hg19_cosmic_OICR_v54_120711.vcf --input_file:tumor tumor.sorted.filtered.collapsed.merged.bam --input_file:normal blood.sorted.filtered.collapsed.merged.bam --intervals chrN:1-NNNNN --out chrom/MuTect.call_stats.chrN.txt --coverage_file

chrom/MuTect.coverage.wig.chrN.txt --vcf chrom/MuTect.chrN.vcf
vcf=`ls chrom/*vcf ; vcf-concat $vcf | vcf-sort > MuTect.allchrom.vcf
Vcf files from each caller were analyzed with custom scripts that ran Annovar (variant_function,exonic_variant_function) and appended this information into the info field:
convert2annovar.pl --format vcf4 --includeinfo caller.vcf > caller.annovar; annotate_variation.pl -geneanno caller.annovar -buildver hg19 /path/to/annovar/humandb
addAnnovarToVCF.pl (in house script)

SIM

SIMs were called jointly with SSMs (see above).

MUTATION FILTRATION

The final merge utilized an in-house scripts that captured calls found from both Strelka and MuTect analyses. Some field names were reworked to allow inclusion from both sources. Headers were merged and annotated with source caller. Genotypes (GT) were taken from the MuTect SNV calls as Strelka does not provide these. For indels called by strelka, genotype calls were formed by review of the data.

COMPUTATIONAL RESOURCES

(no details specified)

CLL.N

FASTQ PROCESSING

No trimming

MAPPING AND BAM PROCESSING

Alignment
BWA alignment. Alignment against the hg19 reference genome was done for each of the two samples using a set of custom perl scripts and perl libraries that implemented the following generalized commands: (bwa 0.5.7, picard 1.9.2)

- bwa aln -t 4 -f R1.sai hg19.random.fa R1.fastq.gz / bwa aln -t 4 -f R2.sai hg19.random.fa R2.fastq.gz
- bwa sampe -f lane.sam hg19.random.fa R1.sai R2.sai R1.fastq.gz R2.fastq.gz
- java -Xmx16g -jar AddOrReplaceReadGroups.jar RGCN=oicr.on.ca RGLB=library RGPL=platform RGPU=none RGSM=sample INPUT=lane.sam OUTPUT=lane.rg.bam VALIDATION_STRINGENCY=LENIENT SO=coordinate
- java -Xmx16g -jar MergeSamFiles.jar INPUT=lane1.rg.bam ... INPUT=laneN.rg.bam OUTPUT=sample.merged.bam SORT_ORDER=coordinate ASSUME_SORTED=no USE_THREADING=yes VALIDATION_STRINGENCY=LENIENT

Index BAM files using Picard (1.9.2)

BAM Processing
Indel realinement and base recalibration using GATK (2.4.9)
Merge the GATK processed normal and tumor BAM files using Picard (1.107)
Index the merged BAM files using Picard (1.107)

MUTATION CALLING

SSM

Strelka v1.0.12. Strelka config.ini contains the following values (used the config file for BWA sampe and isSkipDepthFilters was turned off)

- binSize = 25000000
- depthFilterMultiple = 3.0
- extraStrelkaArguments =
- indelMaxIntHpolLength = 14
- indelMaxRefRepeat = 8

- indelMaxWindowFilteredBasecallFrac = 0.3
- isSkipDepthFilters = 1
- isWriteRealignedBam = 0
- maxInputDepth = 10000
- minTier1Mapq = 20
- minTier2Mapq = 5
- sindelNoise = 0.000001
- sindelPrior = 0.000001
- sindelQuality_LowerBound = 30
- snvMaxFilteredBasecallFrac = 0.4
- snvMaxSpanningDeletionFrac = 0.75
- ssnvNoise = 0.0000005
- ssnvNoiseStrandBiasFrac = 0.5
- ssnvPrior = 0.000001

SIM

SIMs were called using the same criteria as for SSM (see above).

MUTATION FILTRATION

SSM

- Add GT in the FORMAT field and genotype in the NORMAL and TUMOR fields based on SGT in INFO field.
- Apply filters.
    - Whitelist
        - COSMIC (mutations that have occured in > 1 sample).
    - Blacklists
        - dbSNP137
        - 1kGenomes
        - Inhouse failed-to-validate blacklist
        - Fuentes 2012
        - Encode Dac/Duke
        - Duplicate gene database

SIM

Same as for SSMs.

COMPUTATIONAL RESOURCES

(no details specified)

CLL.O

FASTQ PROCESSING

All lanes were used to create the sample bam file. The trimming option of BWA should avoid mapping of poor quality reads.

MAPPING AND BAM PROCESSING

Our mapping pipeline combines the following tools:
Core mapping with BWA v0.5.9:

- bwa aln -l 32 -t 6 -q 20 (6 CPUs used)
- bwa sampe -P

We use '-q 20' option of BWA to trim 3' end of the reads. Reads are trimmed down to a point where the remaining bases are above a quality threshold of 20 (see BWA documentation). But a read could not be shorter than 35bp. This trimming is done before BWA tries to map back the read to the reference genome.
Presently 'bwa sampe' is called within a Perl wrapper and pairs with both reads unmapped are written to a separate BAM file.

Bam processing:

- Sorting + indexing lane BAM file (Picard tools v1.73, standard parameters)
- Indel realignment at known sites (GATK2 v2.0-23, standard parameters)
- Duplicates Marking (Picard, standard parameters)
- Recalibration (GATK2, standard parameters)
- Picard MergeSamFile
- Picard Markduplicates (with remove duplicates option)

We merged all the lanes that passed the quality controls.
We removed the duplicates to obtain a BAM file at the sample level.

MUTATION CALLING

SSM

We called mutations with the new published version of MuTect (v1.1.4).

SIM

We used SomaticIndelDetector, a tool included in GATK2 to call small indels.

MUTATION FILTRATION

SSM

We selected about 24k calls from the output of MuTect and filtered them according to S. Nik-Zainal:

- base quality of mutant alleles
- mapping quality of reads carrying mutant allele
- the number and the position in the read of the mutant allele in the tumor sample
- the phase of reads (balanced or not) carrying the mutant allele
- the position of the mutant allele within boundaries of known repeated, centromeric and telomeric regions
- the presence in a panel of 20 other germline samples sequenced with Illumina protocol
- the presence of the mutant allele in the germline sample
- the strand of reads (balanced or not) carrying the mutant allele
- min number of mutant allele in tumor sample

According to the thresholds used in the filters, calls were classified either "high confidence" or "low confidence".

SIM

- min number of reads carrying indel
- mapping quality of reads carrying indel
- germline filtering
- strand bias filtering
- mismatches in mapping of reads carrying indel (number and windows around indel filtering)
- base quality around indel
- the position of indels within boundaries of known repeated, centromeric and telomeric regions
- mutation filtering (indel starts at a SNV position)

- position of indels in the carrying reads
- the presence of the indel in a panel of 20 other germline samples sequenced with Illumina protocol

COMPUTATIONAL RESOURCES

See **Supplementary Table 18**.

All analysis were done on a Linux cluster (Scientific Linux 5.4) made of:
Number of nodes: 64
Nodes characteristics: 8 cores, 48Gb RAM
Shared disk file system: GPFS, 170To
Job Scheduler: TORQUE
We give here CPU time for two different representative lanes:
Lane1 = HiSeq lane of 91,098,526 pairs
Lane2 = GAIIx lane of 41,702,174 pairs

Presently, we do not have a simple way to keep track of shared memory usage during job execution.
The somatic mutation calling is done in parallel per chromosome. The longest run took 2h45.
The filtering process takes about 30 min (parallel processing).

CLL.P

FASTQ PROCESSING

(no details specified)

MAPPING AND BAM PROCESSING

(no details specified)

MUTATION CALLING

SSM

Sidrón computes a parameter, called S, that can be used to predict whether a candidate mutation in a pileup reflects an actual genomic mutation or can be attributed to noise from the sequencing machine. The noise is estimated from a large collection of genomic sites which are known or strongly suspected to contain no mutations. Then, the S value is calculated for each position, and pre-determined cut-off points are used to predict the genotype. Finally, positions classified as somatic mutations must pass a series of filters based on putative alignment problems.

The algorithm:
Get a set of probably homozygous positions and extract their pileups. When SNP microarrays are available, we extract these positions from their results. If not, we select positions with coverage higher than 12 and the same read base in at least 90% of the covering reads. In either case, we use about 300,000 positions for the next step.
Use the Perl script set_model.pl to calculate and save the estimated error table. The script assumes that the real base is the most frequently read base in each position. Then, the script populates a table where real bases (br) are related to observed bases (bo). These frequencies are broken down into quality tiers according to the Illumina score: low (0-9), medium-low (10-19), medium-high (20-29) and high (30 or higher). See **Supplementary Figure 48** for example for a part of a table.

Assemble a pileup file with all the candidate positions where a tumor mutation is expected (TD.mq). This step can be performed with the filtering programs in Samtools and Bcftools. The filtering conditions are set to non-restrictive, so that lots of false positives are expected. The same positions are extracted from the alignment of the normal (constitutive) sample with samtools into TD.fi (samtools mpileup -f path_to_genome -l TD.mq normal_bam_file > TD.fi).
Use the estimated error table with the sidron.pl Perl script to set an S score for each candidate position. Here is one example:

```
1	12198	G	9	.CCc.cc.C	GGGFGEEG(	1	12198	G	8	,CCCcc.c	GEGGGGGA
	CG	9.09767573371737	CG	5.45076161802802	-
1	12241	G	8	.,,..,c,	DGGEGF>E	1	12241	G	7	..,,C,. FEGGEGG GC
	1.12288376368371	GC	1.81874122037704	-
1	12245	T	7	,,C.,,, FG6GFE@ 1	12245	T	8	..,,.,.n	GFGGFGG#	TC	0.316737975803436
	TA	-2.00356890319781	-
1	12279	G	4	a,,,	(GEA	1	12279	G	4	.,,,	EB#>	GA
	0.307362715253917		GC	-1.19634829871803	-
1	12339	G	3	,,a	GG:	1	12339	G	3	,,,	GGG	GA
	1.41930421006339	GC	-0.902935359473031		-
1	12539	G	3	..T	BE6	1	12539	G	7	....... ?=CGGBC GT
	1.90250135748649	GC	-2.10670068976051	-
```

Each line contains the pileup information for one position plus the corresponding scores. Columns 1-6 correspond to the tumor sample. Columns 7-12 correspond to the normal sample. Column 13 explains the possible genotypes in the tumor sample. Only two genotypes are considered in this version: homozygous for the most frequent base (Hz) and heterozygous for the most frequent and second most frequent bases (Het), In the first line, Hz would be CC and Het would be CG. Column 14 shows the S score for the tumor. Finally, columns 15 and 16 show the possible phenotypes and the S score, respectively, for the normal sample. The definition of S represents how Het and Hz explain the pileup configuration:

S = log(p(configuration | Het)/p(configuration | Hz))

Therefore, higher S scores are compatible with Het positions and lower scores predict Hz positions.

Use the cutoff points to classify somatic mutations. The cutoff points were set after confirming the genotypes of 200 random positions and refined with a Monte Carlo simulation. The Monte Carlo simulation set 10,000 pileup positions consistent with a typical error table, either Het or Hz, at several read depths (5, 10, 20, 50, 100 and 200). Then, the S values were calculated and cutoff points were selected to achieve less than 5% false positives (predicted Het when in fact Hz) and as high a sensitivity as possible. Depths lower than 6 were subsequently discarded.

SIM

SIMs were called using the same pipeline as for SSMs (see above).

MUTATION FILTRATION

Use the Perl script polyfilter.pl to filter out putative sequencing and alignment artifacts. This scripts processes each pileup position and looks for three hallmarks of putative artifacts:

- The somatic mutation creates a polyN stretch of 10 or more nucleotides. Sometimes, in the context of near-polyN stretches (i. e. AAAAGAAAAAA), the sequencing machine completes the polyN (in the example, AAAAAAAAAAA) even in the absence of a mutation. These positions are eliminated from the result.
- Reads supporting a mutation which can align at other sites in the genome according to BLAT (local installation) are eliminated from the result.
- If all of the reads pointing to the mutation contain the mutant nucleotide at similar positions, the position is eliminated from the result. This typically arises from alignment artifacts in the proximity of small indels.
- Repeat the sidron.pl step. This is necessary because some pileup positions may have been changed by polyfilter.pl.

COMPUTATIONAL RESOURCES

(no details specified)

CLL.R

FASTQ PROCESSING

Trim-per-lane, trimmomatic !30 minLen 32

MAPPING AND BAM PROCESSING

Align-per-lane bwa 0.6.2 aln read1
Align-per-lane bwa 0.6.2 aln read2

Align-per-lane bwa 0.6.2 sampe
merge lane picard 1.77
indel realign GATK 2.1 target+realign
mergeRealigned+query sort picard 1.77
fixmate + coord sort picard 1.77
MarkDup picard 1.77

MUTATION CALLING

samtools mpileup by regions of 5,000,000

MUTATION FILTRATION

(no details specified)

COMPUTATIONAL RESOURCES

Trim-per-lane, 1hr
Align-per-lane bwa aln, 1-4hr
Align-per-lane bwa sampe, 4-8hr
merge lane picard, 10hr
indel realign GATK target+realign, 1-7hr
mergeRealigned+query sort picard, 21hr
fixmate + coord sort picard, 21hr
MarkDup picard, 16hr
samtools mpileup by regions of 5,000,000

CLL.S

FASTQ PROCESSING

Quality trim – quality 2 or lower trimmed off end.

MAPPING AND BAM PROCESSING

SNAP (http://snap.cs.berkeley.edu/). We used the default settings for all parameters but max hits, which we increased to 1000 (-h 1000).

MUTATION CALLING

SSM

BamBam

- 2 minimum reads to support SNPs
- minimum base quality of 10
- minimum neighborhood quality of 10 (5 surrounding bases must have base quality 10 or higher)
- minimum mapping quality of 10 (made no difference, mapping software only has mapping qualities of 60 and 0)
- 10% expected fraction germline
- maximum base quality of 40

COMPUTATIONAL RESOURCES

Run on a cluster environment with 2.33GHz Xeon processors (4 years old)
25 compute-hours for initial results
1000 compute-hours for post processing and VCF creation (this number will be reduced in the future, it's just absurdly

high right now.

CLL.T

FASTQ PROCESSING

(no details specified)

MAPPING AND BAM PROCESSING

Novoalign version 2.07.14, Picard version 1.56, samtools 0.1.18 :
- Novoalign with parameters: -i PE 400,50 -r ALL 1 -R 0 and Picard SortSam.jar to create lane level BAM files
- Samtools view to filter on -F 4, -F 256, -q 30
- Picard MarkDuplicates.jar to merge and remove duplicates (REMOVE_DUPLICATES=true)

GATK version 1.3.16, Picard version 1.40. All steps are parallelized by chromosome save for the CountCovariates step:
- GATK -T RealignerTargetCreator with --known dbSNP135 to generate realignment targets
- GATK -T IndelRealigner with --known dbSNP135 to locally realign around indels
- Picard FixMateInformation.jar to update pairs after realignment
- GATK -T CountCovariates with -cov ReadGroupCovariate -cov QualityScoreCovariate -cov CycleCovariate - cov DinucCovariate to build the covariate file
- GATK -T TableRecalibration with --preserve_qscores_less_than 5 to apply the covariates to the base
- qualities

MUTATION CALLING

SSM

Our existing production pipeline calls SSMs and SIMs using GATK and an in-house filtering script. For this benchmarking task we have submitted the intersection of our GATK and VarScan calls as our high confidence set, and the calls private to each tool as our medium confidence set.

GATK was run as follows:
- GATK -T UnifiedGenotyper with --dbsnp dbSNP135 -stand_call_conf 30 -stand_emit_conf 1.0 -- computeSLOD

VarScan was applied as follows:
- normal_pileup="samtools mpileup -B -q 1 -f hg19_random.fa normal.bam"
- tumor_pileup="samtools mpileup -B -q 1 -f hg19_random.fa tumor.bam"
- VarScan.v2.3.2 somatic <(\$normal_pileup) <(\$tumor_pileup) --min-coverage 8 --min-var-freq 0.1 --p- value 0.05 --somatic-p-value 0.05 --output-vcf 1
- VarScan.v2.3.2 somaticFilter snp.vcf --min-coverage 8 --min-reads2 3 --min-avg-qual 20 --min-var-freq 0.1 --p-value 0.05 --output-file snp.hc.vcf
- bam-readcount -b 15 -q 1 -f hg19_random.fa -l snp.hc.vcf.pos tumor.bam > snp.hc.rc
- fpfilter.pl --snp-file snp.hc.vcf --readcount-file snp.hc.rc"

SIM

- GATK -T UnifiedGenotyper with -glm INDEL --dbsnp dbSNP135 -stand_call_conf 30 -stand_emit_conf 1.0 – computeSLOD

CNV

Two in-house tools were used to identify 5 obvious regions of copy number variation based on the lower heterozygous allele frequency and the ratio of normalized depth of coverage between the normal and tumor. These measures were visualized, regions of interest were identified by eye and breakpoints were called manually from the depth of coverage data.

MUTATION FILTRATION

SSM

GATK -T VariantFiltration with --clusterWindowSize 10 --clusterSize 3 --filterExpression "MQ0 >= 4 && ((MQ0 / (1.0 * DP)) > 0.1)" --filterName "HARD_TO_VALIDATE" --mask .$VCF_DIR/${bname}.realigned.recal.bam.indels.raw.${j}.vcf --maskName InDel --filterExpression "DP < 5 " --filterName "LowCoverage" --filterExpression "QUAL < 30.0 " --filterName "VeryLowQual" -- filterExpression "QUAL > 30.0 && QUAL < 50.0 " --filterName "LowQual" --filterExpression "QD < 2.0 " -- filterName "LowQD" --filterExpression "FS > 60.0" --filterName "StrandBiasFishers" --filterExpression "SB > -0.1" --filterName "StrandBias" --filterExpression "DP > 500 " --filterName "ExcessiveDepth"

An in-house script is used on the GATK calls to apply annotation (with ANNOVAR) and to filter based on GATK qualities and hits in dbSNP, and to sort the calls into germline and somatic as follows:
Somatic candidates were initially identified as calls with a differing genotype between a reference (normal tissue) and tumor sample (always a pairwise comparison). Candidate somatic calls were then passed through the following filtering criteria in addition to the above GATK filters:

- dbSNP filtering: All mutations were annotated with dbSNP v135. Somatic candidates with a dbSNP annotation and known frequency > 5% for any population were removed from the set of somatic candidates. dbSNP annotated somatic candidates with no frequency information were retained, as were non-annotated candidate somatic mutations.
- For each sample genotype call, the GATK unified genotyper declares a confidence score for the genotype call, ranging from 0.1 (extremely low confidence) to 99 (very high confidence). Where the reported confidence is < 60.0 for either the reference sample genotype call or the tumor sample genotype call, the somatic call is classified as a 'lower confidence' candidate and is not reported.
- Genotype calls that are heterozygous in the reference tissue, and reported homozygous for one of the reference alleles in the tumor tissue are classified separately as LOH candidates and are not reported. Further verification using copy number data is recommended.
- Finally, somatic candidate mutations that intersect with the UCSC repeat mask, simple repeats, segmental duplications tracks are removed from the somatic candidate list.

SIM

GATK -T VariantFiltration with --filterExpression "MQ0 >= 4 && ((MQ0 / (1.0 * DP)) > 0.1)" --filterName "HARD_TO_VALIDATE" --filterExpression "FS > 60.0" --filterName "StrandBiasFishers" --filterExpression "SB > -10.0" --filterName "StrandBias" --filterExpression "DP < 5" --filterName "LowCoverage" -- filterExpression "QUAL < 30.0" --filterName "VeryLowQual" --filterExpression "QUAL > 30.0 && QUAL < 50.0 " --filterName "LowQual"

COMPUTATIONAL RESOURCES

Everything was run on an SGE Cluster with 5500 3GHz cores arranged in 16 and 24 core nodes:
- 185 nodes with 16 GB RAM
- 221 nodes with 24 GB RAM
- 32 nodes with 96 GB RAM
- 5 nodes with 256 GB RAM

2.5 PB of online storage
1Gb, 10Gb and fibre connectivity
Nodes do not employ locally mounted storage - all scratch is provisioned by NFS.

24 Novoalign Jobs:

- 24 GB of RAM per job = 576 GB
- Average of 7,650 mins of CPU per job = 183,600 mins total
- Average of 10.5 wall clock hours per job

1 GATK (228 Jobs):

- Between 6 and 20 GB of RAM per job = 3,026 GB total requested (max of 500 GB concurrent)
- Total of 19,416 mins of CPU
- ~62 wall clock hours total

VarScan and our in-house CNV tools run in relatively short amounts of time compared to GATK and Novoalign.

CLL.U

FASTQ PROCESSING

(no details specified)

MAPPING AND BAM PROCESSING

bwa 0.5.9 [-q 5 -t 4]
Duplication handling and BAM merging: Picard 1.46

MUTATION CALLING

SSM

(samtools r963 [-A -B] filtered by snp-filter v1
intersect
somaticsniper 1.0.2 [-F vcf -q 1 -Q 15] filtered by false-positive v1 [-- bam-readcount-version 0.4 --bam-readcount-min-base-quality 15]
then somatic-score-mapping-quality v1 [--min-mapping-quality 40 -- min-somatic-score 40])
unique union
(varscan-somatic 2.2.6 filtered by varscan-high-confidence v1 then
false-positive v1 [--bam-readcount-version 0.4 --bam-readcount-min- base-quality 15])
unique union
(strelka 0.4.6.2 [isSkipDepthFilters = 0])

SIM

(gatk-somatic-indel 5336 filtered by false-indel v1 [--bam-readcount- version 0.4 --bam-readcount-min-base-quality 15])
unique union
(pindel 0.5 filtered by pindel-somatic-calls v1 then pindel-vaf-filter v1 [-- variant-freq-cutoff=0.08] then pindel-read-support v1)
unique union
(varscan-somatic 2.2.6 filtered by varscan-high-confidence-indel v1 then false-indel v1 [--bam-readcount-version 0.4 –bam-readcount- min-base-quality 15])
unique union
(strelka 0.4.6.2 [isSkipDepthFilters = 0])

MUTATION FILTRATION

See above Mutation Calling.

COMPUTATIONAL RESOURCES

Compute cluster with 4 types of hosts:
Type-I : 2 x four (Xeon @ 2.50 GHz), 32G RAM, 300GB HDD, PowerEdge M600
Type-II : 2 x four (E5540 @ 2.53GHz), 48G RAM, 600GB HDD, PowerEdge M610
Type-III : 2 x six (X5660 @ 2.80GHz), 96G RAM, 1TB HDD, PowerEdge M610
Type-IV : 2 x six (E5-26030 @ 2.30 GHz), 96G RAM, 2TB HDD, PowerEdge M620

Platform LSF job scheduler has access to:
405 total hosts
810 total CPUs
3520 total cores
4787 total job slots
Runtime: 2 days and 06:30:54
CPU time: 11 days and 07:02:11

Medulloblastoma Somatic Mutation Calling Pipeline Descriptions

MB.A

FASTQ PROCESSING

(no details specified)

MAPPING AND BAM PROCESSING

Read sequences were mapped by BWA v0.5.8a to the human reference genome (GRCh37) using default options.
Possible PCR duplicated reads were removed by SAMtools v 0.1.18 using default options.
After filtering by pair mapping distance, mapping uniqueness and orientation between paired reads, the mapping result files were converted into pileup format by SAMtools with -scf option.
We used three kinds of read filters:

- set1: both read pairs were uniquely mapped with consistent orientation and pair distance (within average ± 3 s.d.).
- set2: at least one read pair was uniquely mapped with consistent orientation and pair distance.
- set3: all uniquely mapped paired reads and set2.

MUTATION CALLING

SSM

Calls were made using all three sets of filtered reads, and mutations identified in the all three sets were considered as candidates.
The scripts for SSM and SIM calling are available from http://emu.src.riken.jp.

SIM

Calls were made using all three sets of filtered reads, and mutations identified in the all three sets were considered as candidates.
The scripts for SSM and SIM calling are available from http://emu.src.riken.jp.

MUTATION FILTRATION

SSM

- non-reference calls with a frequency ≥ 0.15 after removing bases calls with base quality < 10, and mapping quality < 20
- supported by at least two base calls including one base call with base quality ≥ 30
- a SAMtools consensus quality ≥ 20 and root mean square mapping quality ≥ 40
- if three or more single nucleotide mutations were found within any 10bp windows, or distance from nearest indel was less than 5bp, we discarded all SNVs
- if candidate non-coding SNVs were in a tandem repeat region suggested by tandem repeat finder, we discarded the SNVs
- if candidate SNVs were in RepeatMasker repeat regions (http://www.repeatmasker.org) within 1Mb from the centoromeric or telemeric gaps, we discarded the SNVs
- if a base with consensus quality lower than 20 occurs within 3bp on either side of the target SNV, we discarded the SNVs
- After SNV calling in the tumor samples, SSMs were filtered based on the lymphocyte sequence of the same patient:
- SSM alleles with a frequency ≥ 0.03 after removing reads with base quality < 15, and mapping quality < 20
- depth of coverage in lymphocyte ≤ 7
- depth of coverage in lymphocyte ≤ 10 and SSM allele was represented in the dbSNP database v131.

SIM

- if indels were supported by a frequency ≥ 0.1 and ≥ 4 reads after removing reads with mapping quality < 20, and root mean square mapping quality ≥ 40

- if candidate non-coding indels were in repeat regions suggested by tandem repeat finder or RepeatMasker, we discarded the indels.
- After indel calling in tumor samples, the candidates were filtered based on the lymphocyte sequence of the same patient using the following criteria:
- depth of coverage in lymphocyte ≤ 7
- for coding and non-coding region, if any indels were identified within 5bp or 10bp region in lymphocyte, respectively, the candidate indel was discarded.

COMPUTATIONAL RESOURCES

(no details specified)

MB.B

FASTQ PROCESSING

(no details specified)

MAPPING AND BAM PROCESSING

The BAMs were provided by CNAG.

MUTATION CALLING

SSM

SSMs were first predicted by Varscan:
SAMtools mpileup parameter was –Q 0
Varscan parameters were --min-coverage 10 --min-coverage-normal 10 --min-coverage-tumor 10 --min-var-freq 0.1 --min-freq-for-hom 0.75 --somatic-p-value 0.05 --min-avg-qual 0

SIM

SIMs were first called using GATK SomaticIndelDetector using default parameters. For the SIMs and their flanking regions of 500bp, normal reads and tumor reads were realigned to hg19.
Then we uses Varscan to identify SIMs:
SAMtools mpileup was run with parameters –Q 0
Varscan was run with parameters --min-coverage 5 --min-coverage-normal 5 --min- coverage-tumor 5 --min-var-freq 0.1
Distance between adjacent SIM and to adjacent SSM had to be >10bp.

MUTATION FILTRATION

SSM

Filter parameters of high confidence results:
- Adjacent somatic mutation distance > 10
- The number of alternative allele reads >= 5
- Alternative allele frequency in the matched normal sample < 0.05
- Reads mapping to this mutations are not significantly enriched for mismatches (p-value>0.005)
- Reads with best hits greater than 1 are not significantly overrepresented at this position (P-value>0.01)
- Map Quality score not significant lower in mutated reads than other alleles (Map Quality score cutoff is 30. Fisher's exact test, p < 0.01)
- Base Quality score for mutated position not significantly lower than other alleles (Base Quality score cutoff is 20. Fisher's exact test, p < 0.05)
- Mutant allele frequency change between tumor and adjacent normal (Fisher's exact test p < 0.05)
- Mutations were not in gap aligned reads (in neither 20bp flank region less than 10 gap flags)

- Mutant allele not significantly enriched within 5 bps of 5' or 3' ends of reads (Fisher's exact test, p < 0.05)
- Mutations were not in simpleRepeatRegion (Repeat events less than 6) , simpleRepeatRegion (http://hgdownload.cse.ucsc.edu/goldenPath/hg 19/database/simpleRepeat.txt.gz)

SIM

Filter parameters of high confidence results:
- Indels were not annotated in the 1000 Genomes Project indels database
- The number of alternative allele reads >= 5
- Reads mapping to this mutations are not significantly enriched for mismatches (P-value>0.005)
- Reads with best hits greater than 1 are not significantly overrepresented at this position (P-value>0.01)
- Map Quality score not significantly lower in mutated reads than other alleles (Map Quality score cutoff is 30. Fisher's exact test, p < 0.01)

COMPUTATIONAL RESOURCES

(no details specified)

MB.C

FASTQ PROCESSING

(no details specified)

MAPPING AND BAM PROCESSING

reference genome:
Reference genome: hsapiens_v37.fa (GRCh37 downloaded in May 2010)

tools and versions:
Picard version: 1.73
Samtools and bcftools version : 0.1.19
GATK version: 1.6.5 and 2.5
snape-pileup (CNAG, Emanuele Raineri)
snpEff 2.0.5d
vcfProcess (CNAG, Simon Heath)
vcfPileupFT (CNAG, Francesc Castro/Sophia Derdak/Raul Tonda)
vcf-somatic (CNAG, Francesc Castro)

Bam file processing:
Steps performed for each read group separately:
- starting from uniquely mapped, properly paired reads generated by the gem mapping pipeline + selReads script (CNAG, Simon Heath)
- sort by coordinates to make bam file compatible with GATK: picard ReorderSam
- add read groups: picard AddOrReplaceReadGroups
- since our bam files lack mapping qualities (mapping quality field always says 255), we reassign mapping quality to make bam file compatible with GATK:
- SAMTOOLS/0.1.19/bin/samtools view -h readgroups.bam | awk 'BEGIN{OFS="\t"}{if($5 == 255){$5 = 35}; print $0}' | SAMTOOLS/0.1.19/bin/samtools view -Sb
- index processed bam file: samtools index

Steps performed for each sequencing library separately (joining bam files from several read groups):
- remove duplicates: picard MarkDuplicates

Steps performed taking together all bam files for both samples:

- local realignment using two "gold standard" datasets of human indels from the former GATK bundle of RESOURCES for the human genome: GATK RealignerTargetCreator, GATK IndelRealigner, GATK LeftAlignIndels

MUTATION CALLING

SSM

Mutation calling per sample:

- snape-pileup on the rmdup.bam files

Mutation calling on the sample-pair realigned bam files:

- Mutation calling including the -d option to allow high coverage per sample to avoid downsampling (and thus potential miscalling) at high coverage positions:
  - SAMTOOLS/0.1.19/bin/samtools mpileup -DSug -d 10000 leftaligned.bam | SAMTOOLS/0.1.19/bin/bcftools view -bvcg - | SAMTOOLS/0.1.19/bin/bcftools view –

vcf file processing:

- left align mutations: GATK LeftAlignAndTrimVariants
- mutation annotation: snpEff using GRCh37.65 database

soft filtering and joining samtools and snape results:

- vcfProcess

Fisher test for allele frequencies at positions with difference in genotype between control and tumor:

- vcfPileupFT

SIM

SIMs were called jointly with SSMs as described above.

MUTATION FILTRATION

SSM

tagging of somatic candidates: vcf-somatic, using the following parameters:

- genotype 0/0 in the control sample and 0/1 in the tumor sample
- Fisher Test p-value for the position <=0.01
- alternative allele frequency in the control sample <=0.05
- alternative allele frequency in the tumor sample >=0.10
- count of the alternative allele in the control sample <=1
- count of the alternative allele in the tumor sample >=2
- coverage in the control sample >=10
- coverage in the tumor sample >=10
- Genotype quality in the control sample >=20
- Genotype quality in the tumor sample >=20
- exclude positions that were 0/1 in the tumor and had less 15 reads in one of the samples.
- exclude positions on chrM.

SIM

SIMs were filtered jointly using the same criteria as for SSM (see above).

COMPUTATIONAL RESOURCES

(no details specified)

MB.D

The SMUFIN[7] pipeline was used.

MB.E

FASTQ PROCESSING

FASTQ files were processed using our QC and alignment pipelines as follows:
Data quality was assessed using FastQC.
Second read for each of the three 251bp paired end MiSeq runs (A3MCW, A4DBD and A4DC6) was trimmed to 220 bases.

MAPPING AND BAM PROCESSING

BWA v0.5.9 in paired end mode (bwa aln, bwa sampe) using default settings
GRCh37 reference from Ensembl v71 with chromosomes renamed using UCSC hg19 naming scheme
Picard v1.105 FixMateInformation
Picard v1.105 MarkDuplicates treating 514F-A and 514F-B as separate libraries

MUTATION CALLING

SSM

SSMs were called and filtered as follows:
SomaticSniper v1.0.2 with following settings: -q 1 -Q 15 -J -r 0.001000 -T 0.850000 -N 2 -s 0.01

SIM

(no details specified)

MUTATION FILTRATION

SSM

False positive filters using fpfilter.pl script from SomaticSniper/VarScan2 (all apply to tumor reads):

- Average mutation position in supporting reads relative to read length between 0.1 and 0.9
- Strandedness - fraction of supporting reads from forward strand between 0.01 and 0.99
- Mutant read count >= 4
- Mutant allele frequency >= 0.05
- Difference in average mismatch quality sum between mutant and reference reads <= 50
- Average mismatch quality sum for mutant reads <= 100
- Difference in average mapping quality between reference and mutant reads <= 30
- Difference in average read length between reference and mutant reads <= 25
- Additional filters:
- Homopolymer - number of bases in a flanking homopolymer < 5
- Normal genotype must contain reference base, e.g. 0/0 or 0/1 but not 1/2
- Depth in normal >= 10
- Mutant read count in normal <= 1
- Indel proximity - exclude mutations within 40bp of indel called by Pindel or samtools mpileup in either the tumor or the normal
- Exclude mutations at positions of known SNPs from the NHGRI UniSNP database of uniquely mapped SNPs from dbSNP v129 and HapMap release 27
- Somatic score from SomaticSniper >= 40

SIM

(no details specified)

COMPUTATIONAL RESOURCES

(no details specified)

MB.F

FASTQ PROCESSING

FASTQ files were processed using our QC and alignment pipelines as follows:
Data quality was assessed using FastQC.
Second read for each of the three 251bp paired end MiSeq runs (A3MCW, A4DBD and A4DC6) was trimmed to 220 bases.

MAPPING AND BAM PROCESSING

BWA v0.5.9 in paired end mode (bwa aln, bwa sampe) using default settings.
GRCh37 reference from 1000 Genomes project (includes rCRS mitochondrial sequence, human herpes virus 4 type 1 and concatenated decoy sequences, hs37d5) with chromosomes renamed using UCSC hg19 naming scheme.
Picard v1.105 FixMateInformation
Picard v1.105 MarkDuplicates treating 514F-A and 514F-B as separate libraries

MUTATION CALLING

SSM

SSMs were called as follows:
Strelka v1.0.13 run with default/suggested configuration settings for BWA:

- isSkipDepthFilters = 0
- maxInputDepth = 10000
- depthFilterMultiple = 3.0
- snvMaxFilteredBasecallFrac = 0.4
- snvMaxSpanningDeletionFrac = 0.75
- indelMaxRefRepeat = 8
- indelMaxWindowFilteredBasecallFrac = 0.3
- indelMaxIntHpolLength = 14
- ssnvPrior = 0.000001
- sindelPrior = 0.000001
- ssnvNoise = 0.0000005
- sindelNoise = 0.000001
- ssnvNoiseStrandBiasFrac = 0.5
- minTier1Mapq = 20
- minTier2Mapq = 5
- ssnvQuality_LowerBound = 15
- sindelQuality_LowerBound = 30

SIM

SIMs were called using the same criteria as for SSM (see above).

MUTATION FILTRATION

SSM

SSM calls from Strelka were further filtered as follows:

- False positive filters using fpfilter.pl script from SomaticSniper/VarScan2 on tumor read counts/statistics generated using bam-readcounts (also from WashU) with -q 1 (i.e. excluding reads with zero mapping quality)
  - Mutant read count >= 3
  - Average mutation position in supporting reads relative to read length between 0.1 and 0.9
  - Strandedness - fraction of supporting reads from forward strand between 0.01 and 0.99 (only applied if at least 7 mutant reads)
  - Mutant allele frequency >= 0.05

  - Average mismatch quality sum for mutant reads <= 100
  - Difference in average mismatch quality sum between mutant and reference reads <= 75 (increased from default value of 50 to reduce filtering of true somatic mutations located close to and phased with heterozygous germline SNPs)
  - Difference in average mapping quality between reference and mutant reads <= 30
  - Average distance to 3' end of mutation position in supporting reads relative to read length >= 0.2
- Additional filters
  - Homopolymer - number of bases in a flanking homopolymer < 5
  - Mutant read count in normal <= 1 (using reads with mapping quality >= 1 and bases with quality >= 10)

SIM

Note that Strelka applies a number of filters based on the above settings and these appear in the submitted VCF file. We have not applied any further filtering to the SIM calls.

COMPUTATIONAL RESOURCES

(no details specified)

MB.F2

FASTQ PROCESSING

Quality assessment – FastQC

Trimming – second read for each of the 251-bp paired end MiSeq runs (A3MCW, A4DBD and A4DC6) trimmed to 220 bases.

MAPPING AND BAM PROCESSING

Alignment – BWA v0.5.9 run in paired end mode (bwa aln, bwa sampe) using default settings.
Reference genome – GRCh37 reference from 1000 Genomes project (includes rCRS mitochondrial sequence, human herpes virus 4 type 1 and concatenated decoy sequences, hs37d5) with chromosomes renamed using UCSC hg19 naming scheme.
Post-processing – Picard v1.105 FixMateInformation and MarkDuplicates treating 514F-A and 514F-B as separate libraries

MUTATION CALLING

SSM

Strelka v1.0.13 run with default/suggested configuration settings for BWA with the exception of indelMaxRefRepeat (see SIM calling below).

- isSkipDepthFilters = 0
- maxInputDepth = 10000
- depthFilterMultiple = 3.0
- snvMaxFilteredBasecallFrac = 0.4
- snvMaxSpanningDeletionFrac = 0.75
- indelMaxRefRepeat = 100
- indelMaxWindowFilteredBasecallFrac = 0.3
- indelMaxIntHpolLength = 14
- ssnvPrior = 0.000001
- sindelPrior = 0.000001

- ssnvNoise = 0.0000005
- sindelNoise = 0.000001
- ssnvNoiseStrandBiasFrac = 0.5
- minTier1Mapq = 20
- minTier2Mapq = 5
- ssnvQuality_LowerBound = 15
- sindelQuality_LowerBound = 30

SIM

SIMs were called using Strelka v1.0.13 using the same configuration settings as for SSM (see above). Note that one change was made to the default/suggested settings for BWA – indelMaxRefRepeat was set to an arbitrarily high value of 100 (cf. default value of 8) to effectively disable the Strelka filter for somatic indel calls representing an expansion or contraction of a repeated pattern.

MUTATION FILTERING

SSM

The SSM calls that pass Strelka's filters were further filtered as described in supplementary table 9.

SIM

No further filters have been applied to SIM calls from Strelka that pass Strelka's own filters. Note that the setting used for indelMaxRefRepeat effectively disables this filter.

MB.G

Same as CLL.G (above)

MB.H

FASTQ PROCESSING

(no details specified)

MAPPING AND BAM PROCESSING

Reads from each lane were aligned/preprocessed separately in the following steps:

- alignment with Novoalign (v 2.08.03), non-default parameter values were used to provide sample- and lane-specific read group information, options "-i PE 220,60" and "-r All 10" were used
- reads not aligning to the reference genome were discarded (in-house scripts)
- reads not aligning uniquely to the reference genome were discarded (in-house scripts)
- read-pairs which had both mates mapped with the same orientation and/or each mate mapped to a different chromosome were discarded (in-house scripts)
- duplicate reads (judged by mapping location and sequence) with lowest sum of base qualities were discarded (in-house scripts)
- GATK tools (v. 2.3-9) with default settings were used for indel realignment (RealignerTargetCreator, IndelRealigner) and base-quality recalibration (BaseRecalibrator, PrintReads).
- Picard tools (v 1.84) were used for SAM to BAM conversions (SamFormatConverter), BAM files sorting (SortSam) and consistency maintenance (FixMateInformation).
- Picard tools (v 1.84) were used for merging of the pre-processed lane-separated reads (MergeSamFiles)
- The merged tumor and normal BAM files were split by chromosome for the purpose of parallelizing the

downstream steps (in-house scripts). The following steps were performed on the chromosome-specific BAM files:
- GATK tools (v. 2.3-9) were used for a common (tumor+normal) re-alignment around (RealignerTargetCreator, IndelRealigner)
- Picard tools (v 1.84) were used for BAM files sorting (SortSam)

MUTATION CALLING

SSM

MuTect (v. 1.1.4) was used for chromosome-wise calling and initial classification of SSMs.
In-house scripts were used for generating additional information that could be used for mutation post-filtering (allele strand support information with soft-clip areas being taken into account).

Annotations:
- ANNOVAR (release august 27 2013) using RefSeq as the gene model database
- dbSNP (build138) + 1000G
- COSMIC v68 (February 2014)

SIM

VarScan (v. 2.3.5) was used for chromosome-wise calling and initial classification of INDEL mutations.
SamTools (v. 0.1.18) mpileup was utilized for creating VarScan input files, with option -B enabled.
VarScan's fpfilter (v. 1.01) was used for initial round of indel filtering (default filtering settings was used, except for parameter "min_var_count" being set to 2).
In-house scripts were used for generating additional information that could be used for mutation post-filtering (allele strand support information with soft-clip areas being taken into account).
Mutations were left-aligned using GATK's LeftAlignAndTrimVariants tool.

Annotations:
- ANNOVAR (release august 27 2013) using RefSeq as the gene model database
- dbSNP (build138) + 1000G
- COSMIC v68 (February 2014)

MUTATION FILTRATION

SSM

SSM and SIM annotated as 'PASS' had to pass ALL the following criteria:
- (tumor depth at mutation position >= 14 and support for alternate allele by >= 4 reads) OR (fraction of alternate allele >= 0.2)
- (normal depth at mutation position >= 8 and support for alternate allele by <= 0 reads) OR (fraction of alternate allele <= 0.03)
- PASSED by external software algorithm for somatic mutation detection (MuTect or VarScan2)

SIM

- SIMs (called by VarScan2) had to have read support for alternate allele on both strands
- SIMs (called by VarScan2) had to occur outside a mononucleotide repeat (>=8) and outside high-copy repeats

GERMLINE

GERMLINE mutations annotated as 'PASS' had to pass ALL the following criteria:
- Rejected by external software algorithm for somatic mutation detection (MuTect or VarScan2)
- (normal depth at m position >= 14 and support for alternate allele by >= 4 reads)
- fraction of alternate allele > 0.2

COMPUTATIONAL RESOURCES

(no details specified)

MB.I

FASTQ PROCESSING

(no details specified)

MAPPING AND BAM PROCESSING

All lanes processed passed our quality pipeline and were processed as follows:

- bwa options:bwa 0.6.2-r126-tpx aln -t 12 -q 20 (accelerated hardware, convey)
- sampe options: bwa-0.6.2-tpx sampe -P -T -t 8 -a 1000 -r
- samtools-0.1.17 index
- merge and mark duplicates: Picard tools MarkDuplicates Version: 1.61
- The merged bam files had a coverage (estimated by picard tools) of:
- Control: 29.58x
- Tumor: 40.46x

MUTATION CALLING

SSM

Our SNV calling pipelines does the following steps:

- samtools-0.1.19 mpileup -REI -q 30 -ug (on the tumor bam file)
- filter out mutations with strand bias next to the motif GGnnGG (or CCnnCC if snv is on reverse strand)
- samtools-0.1.19 mpileup -ABRI -Q 0 -q 1 (on the control bam file for positions called in the tumor)
- annotation of databases:
- dbSNP_135/00-All.SNV.vcf.gz
- 1000genomes/ALL.wgs.phase1_integrated_calls.20101123.snps_chr.vcf.gz
- annotate with annovar_Sept2013 -dbtype wgEncodeGencodeCompV17
- annotate with annovar_Sept2013 -regionanno -dbtype segdup
- annotate with annovar_Sept2013 -regionanno -dbtype band
- annotate more tracks:
- UCSC/wgEncodeCrgMapabilityAlign100mer_chr.bedGraph.gz
- UCSC/HiSeqDepthTop10Pct_chr.bed.gz
- UCSC/repeats/SimpleTandemRepeats_chr.bed.gz:4
- UCSC/Sept2013/UCSC_27Sept2013_RepeatMasker.bed.gz
- UCSC/DukeExcluded_chr.bed.gz
- UCSC/DACBlacklist_chr.bed.gz
- UCSC/selfChain_chr.bed.gz
- confidence annotation, this step creates a score for the reliability of a call. Scores are given from 1 to 10. Every call starts with 10 and looses (sometimes even gains) scores based on sequencing depth, VAF, mappability, repeat region, in dbSNP or 1000genomes...

SIM

Our indel calling pipeline uses Platypus_Version_0.5.2.
Calling is done on both, the tumor and control file at the same time; we are currently only looking at somatic calls (genotype 0/0 in control and 0/1|1/0|1/1 in tumor):

- Platypus.py callVariants --ploidy=2 --nIndividuals=2 --nCPU=10 --genIndels=1 --genSNPs=0
- confidence annotation, this step creates a score for the reliability of a call. Scores are given from 1 to 10. Every call starts with 10 and looses based on severity of platypus filters it did not pass and based on call specific properties like sequencing depth, VAF and genotype qualities.

MUTATION FILTRATION

SSM

Filter for high confidence (scores of 8, 9 and 10, see above) somatic calls.

SIM

Filter for high confidence (scores of 8, 9 and 10, see above) somatic calls.

COMPUTATIONAL RESOURCES

(no details specified)

MB.J

FASTQ PROCESSING

(no details specified)

MAPPING AND BAM PROCESSING

The BAMs provided by CNAG were used.

MUTATION CALLING

SSM

This call set is primarily generated by exploration of the joint tumor/normal assembly graph using sga (github.com/jts/sga). In my experience assembly-based calling has two main drawbacks:

- Sensitivity for low-frequency mutations may be limited, due to sga's higher evidence requirement.
- If the evidence in the control sample for a germline SNV/indel is weak, the assembler may call it as a somatic mutation.

To account for the lower sensitivity I have augmented the sga SNV calls with alignment-based calls from freebayes (github.com/ekg/freebayes). To remove false positive mutations due to misalignments, the freebayes calls were filtered against the assembly graph. To account for misclassifed germline SNVs, I implemented a set of simple filters for the sga SNVs using the aligned data.
The union of the sga + freebayes-filtered calls were used for the SNV call set. Only the sga calls were used for the indel set (details below).

Assembly-based calls:
The sga call set used a development version of the code (git SHA1: 13b19a797baf) with the following command:
```
$(SGA) graph-diff -p build2.k61.mdbg2.noextend --min-dbg-count 2 -k 61 -x 4 -t 8 -a debruijn \
        -r variant.fastq.gz -b base.fastq.gz \
        --ref /u/jsimpson/simpsonlab/data/references/hs37d5.pp.fa
```
The calls were post-processed to remove mutations on the decoy chromosomes, left-aligned then filtered with this command:
```
sga-variant-filters.pl --dbsnp ~/simpsonlab/data/references/dbsnp/v138/00-All.vcf.gz \
        --min-af 0.1 \
        --sga sga.allchr.somatic.leftaligned.vcf \
        -o sga.allchr.somatic.leftaligned.filters.vcf \
        --tumor $T_BAM \
        --normal $N_BAM
```

Alignment-based calls:
Freebayes was run on each chromosome in parallel:
```
freebayes -f $REF -r $chr --pooled-discrete --pooled-continuous --min-alternate-fraction 0.1 --genotype-qualities
$T_BAM $N_BAM
```
The freebayes calls were combined, split into single alleles and tagged with somatic status:
```
$VCFLIB/vcfcombine by_chromosome/*.vcf | \
        $VCFLIB/vcfbreakmulti | \
        $VCFLIB/vcfsamplediff -s VT sample_control_MB99_CNAG sample_tumor_MB99_CNAG - >
freebayes.allchr.vcf
```

The freebayes calls were partitioned into a set of somatic calls and all other calls (germline+'reversion').
The somatic calls were checked for concordance with the SGA assembly graph (git SHA1: c4f1c19):
$SGA vcf-read-count -r variant.fastq.gz -b normal.fastq.gz -g $FB_GERMLINE --ref $REF $FB_SOMATIC
The freebayes SNVs that are concordant with the assembly graph and with quality score at least 30 were retained.

SIM

The procedure was

MUTATION FILTRATION

SSM

Merged call set:
To merge the SNV call sets the following logic was applied:

- If an SNV was PASS in either freebayes or SGA, it was PASS in the merged set.
- If only one of two callers found the mutation, the FILTER flag from the caller was retained.

SIM

For indels and MNPs, only SGA calls were used in the merged call set with the FILTER flag from sga-variant-filters.
In the merged call set the genotypes were assumed to be 0/0 for the normal and 0/1 for the tumor.

COMPUTATIONAL RESOURCES

(no details specified)

MB.K

FASTQ PROCESSING

(no details specified)

MAPPING AND BAM PROCESSING

(no details specified)

MUTATION CALLING

SSM

Atlas-SNP of the Atlas2 Suite was run to list all mutations found in multiple reads at a single locus; and mutations were annotated with dbSNP by ANNOVAR and COSMIC (Catalogue Of Somatic Mutations In Cancer).

SIM

Atlas-Indel of the Atlas2 Suite was run to list all the indel mutations; and mutations were annotated with dbSNP by ANNOVAR and COSMIC (Catalogue Of Somatic Mutations In Cancer).

MUTATION FILTRATION

SSM

- Tumor mutation coverage >=4
- Normal/Tumor mutation ratio <1%
- End-of-read: at least one mutant base must be in the center of a read
- Strand direction: at least one mutant base must appear in each direction
- Mapping quality: at least one mutant read must be uniquely mapped
- Normal mutant base quality: Sum of normal mutant base qualities <= 70

- COSMIC mutations were exempted from above filters

SIM

- Tumor/Normal mutation coverage:Tumor mutation coverage >=10, Normal mutation coverage =0
- Tumor mutation ratio >=0.15
- COSMIC mutations were exempted from above filters

COMPUTATIONAL RESOURCES

(no details specified)

MB.L1

FASTQ PROCESSING

(no details specified)

MAPPING AND BAM PROCESSING

Alignment with bwa 0.6.2; the final lane level bam is created by piping output through picard 1.9.0 AddOrReplaceGroups:

- bwa aln -t 8 ref.fa lane.R1.fq.gz > lane.R1.sai ; bwa aln -t 8 lane.R2.fq.gz > lane.R2.fq.gz
- bwa sampe ref.fa lane.R1.sai lane.R2.sai lane.R1.fq.gz lane.R2.fq.gz | java -Xmx2g jar AddOrReplaceGroups.jar RGID.. VALIDATION_STRINGENCY=SILENT I=/dev/stdin O=lane.sorted.bam SO=coordinate CREATE_INDEX=true

Aligned bams are filtered using samtools 0.1.19, then collapsed/deduplicated using picard 1.9.0:

- samtools view -u -F 4 lane.sorted.bam | samtools view -u -F 256 - | samtools view -q 30 - > lane.sorted.filtered.bam
- samtools index lane.sorted.filtered.bam
  java -Xmx4g MarkDuplicates.jar I=lane.sorted.filtered.bam O=lane.sorted.filtered.collapsed.bam METRICS_FILE=lane.sorted.filtered.collapsed.bam.metrics ASSUME_SORTED=true REMOVE_DUPLICATES=true CREATE_INDEX=true

Lane level bams from the same sample are merged into a single file using picard 1.9.0:

- java Xmx4g MergeSamFiles INPUT=lane1.sorted.filtered.collapsed.bam ... INPUT=laneN.sorted.filtered.collapsed.bam OUTPUT=sampleID.sorted.filtered.collapsed.merged.bam SORT_ORDER=coordinate

MUTATION CALLING

SSM

First Somatic Caller: Strelka
Mutation calls were made with strelka/v1.0.7, followed by annotation using GATK 1.3.16.
Strelka config.ini contains the following values:

- isSkipDepthFilters = 0
- maxInputDepth = 10000
- depthFilterMultiple = 3.0
- snvMaxFilteredBasecallFrac = 0.4
- snvMaxSpanningDeletionFrac = 0.75
- indelMaxRefRepeat = 8
- indelMaxWindowFilteredBasecallFrac = 0.3
- indelMaxIntHpolLength = 14
- ssnvPrior = 0.000001
- sindelPrior = 0.000001

- ssnvNoise = 0.0000005
- sindelNoise = 0.000001
- ssnvNoiseStrandBiasFrac = 0.5
- minTier1Mapq = 20
- minTier2Mapq = 5
- ssnvQuality_LowerBound = 15
- sindelQuality_LowerBound = 30
- isWriteRealignedBam = 0
- binSize = 25000000

Extra Strelka arguments:

- configureStrelkaWorkflow.pl --normal blood.sorted.filtered.collapsed.merged.bam --tumor tumor.sorted.filtered.collapsed.merged.bam --ref hg19_random.fa --config config.ini --output-dir analysis

After Strelka:

- cd analysis; qmake -inherit -- -j 16
- java -Xmx4g -Djava.io.tmpdir=tmp -jar GenomeAnalysisTK.jar -T VariantAnnotator -R /hg19_random.fa --variant analysis/results/passed.somatic.snvs.vcf --dbsnp dbSNP137_chr.vcf -o passed.somatic.snvs.vcf

Second Somatic Caller: MuTect
Mutation calls were made with MuTect 1.1.4. MuTect was run in parallel, by chromosome, and the final output was merged with vcftools 0.1.7:

- java -Xmx10g -Djava.io.tmpdir=tmp -jar MuTect-1.1.4.jar --analysis_type MuTect --reference_sequence hg19_random.fa --dbsnp dbSNP137_chr.vcf --cosmic hg19_cosmic_OICR_v54_120711.vcf --input_file:tumor tumor.sorted.filtered.collapsed.merged.bam --input_file:normal blood.sorted.filtered.collapsed.merged.bam --intervals chrN:1-NNNNN --out chrom/MuTect.call_stats.chrN.txt --coverage_file chrom/MuTect.coverage.wig.chrN.txt --vcf chrom/MuTect.chrN.vcf
- vcf=`ls chrom/*vcf ; vcf-concat $vcf | vcf-sort > MuTect.allchrom.vcf

VCF Annotation, filtering and merging:
Vcf files from each caller were analyzed with custom scripts that ran Annovar (variant_function, exonic_variant_function) and appended this information into the info field:

- convert2annovar.pl --format vcf4 --includeinfo caller.vcf > caller.annovar; annotate_variation.pl -geneanno caller.annovar -buildver hg19 /path/to/annovar/humandb
- addAnnovarToVCF.pl (in house script)

An in-house script utilizing tabix/bedfiles was used to identify loci within UCSC/hg19 SegDup, rmsk and SimpleRepeat regions, and these were filtered out of the final output:

- vcf-annotate.pl (in-house script)

SIM

(no details specified)

MUTATION FILTRATION

SSM

The final merge utilized an in-house script that captured calls found in both Strelka and MuTect analyses. Some field names were reworked to allow inclusion from both sources. Headers were merged and annotated with source caller. Genotypes (GT) were taken from the MuTect SNV calls as Strelka does not provide these.

SIM

For indels called by strelka, genotype calls were formed by review of the data.

COMPUTATIONAL RESOURCES

(no details specified)

MB.L2

The Pipeline use to produce this data set is identical to this pipeline: MB.L1
With the following exception (omitted step indicated with strikethrough text):
~~An in house script utilized tabix/bedfiles was used to identify loci withiin UCSC/hg19 SegDup, rmsk and SimpleRepeat regions and these were filtered out of the final output.~~

1. ~~vcf-annotate.pl (in house script)~~

FASTQ PROCESSING

(no details specified)

MAPPING AND BAM PROCESSING

Alignment with bwa 0.6.2; the final lane level bam is created by piping output through picard 1.9.0 AddOrReplaceGroups:

- bwa aln -t 8 ref.fa lane.R1.fq.gz > lane.R1.sai ; bwa aln -t 8 lane.R2.fq.gz > lane.R2.fq.gz
- bwa sampe ref.fa lane.R1.sai lane.R2.sai lane.R1.fq.gz lane.R2.fq.gz | java -Xmx2g jar AddOrReplaceGroups.jar RGID.. VALIDATION_STRINGENCY=SILENT I=/dev/stdin O=lane.sorted.bam SO=coordinate CREATE_INDEX=true

Aligned bams are filtered using samtools 0.1.19, then collapsed/deduplicated using picard 1.9.0:

- samtools view -u -F 4 lane.sorted.bam | samtools view -u -F 256 - | samtools view -q 30 - > lane.sorted.filtered.bam
- samtools index lane.sorted.filtered.bam
- java -Xmx4g MarkDuplicates.jar I=lane.sorted.filtered.bam O=lane.sorted.filtered.collapsed.bam METRICS_FILE=lane.sorted.filtered.collapsed.bam.metrics ASSUME_SORTED=true REMOVE_DUPLICATES=true CREATE_INDEX=true

Lane level bams from the same sample are merged into a single file using picard 1.9.0:

- java Xmx4g MergeSamFiles INPUT=lane1.sorted.filtered.collapsed.bam ... INPUT=laneN.sorted.filtered.collapsed.bam OUTPUT=sampleID.sorted.filtered.collapsed.merged.bam SORT_ORDER=coordinate

MUTATION CALLING

SSM

First Somatic Caller: Strelka
Mutation calls were made with strelka/v1.0.7, followed by annotation using GATK 1.3.16.
Strelka config.ini contains the following values:

- isSkipDepthFilters = 0
- maxInputDepth = 10000
- depthFilterMultiple = 3.0
- snvMaxFilteredBasecallFrac = 0.4
- snvMaxSpanningDeletionFrac = 0.75
- indelMaxRefRepeat = 8
- indelMaxWindowFilteredBasecallFrac = 0.3
- indelMaxIntHpolLength = 14
- ssnvPrior = 0.000001
- sindelPrior = 0.000001
- ssnvNoise = 0.0000005
- sindelNoise = 0.000001
- ssnvNoiseStrandBiasFrac = 0.5
- minTier1Mapq = 20
- minTier2Mapq = 5
- ssnvQuality_LowerBound = 15
- sindelQuality_LowerBound = 30
- isWriteRealignedBam = 0
- binSize = 25000000

Extra Strelka arguments:

- configureStrelkaWorkflow.pl --normal blood.sorted.filtered.collapsed.merged.bam --tumor tumor.sorted.filtered.collapsed.merged.bam --ref hg19_random.fa --config config.ini --output-dir analysis

After Strelka:

- cd analysis; qmake -inherit -- -j 16
- java -Xmx4g -Djava.io.tmpdir=tmp -jar GenomeAnalysisTK.jar -T VariantAnnotator -R /hg19_random.fa --variant analysis/results/passed.somatic.snvs.vcf --dbsnp dbSNP137_chr.vcf -o passed.somatic.snvs.vcf

Second Somatic Caller: MuTect
Mutation calls were made with MuTect 1.1.4. MuTect was run in parallel, by chromosome, and the final output was merged with vcftools 0.1.7:

- java -Xmx10g -Djava.io.tmpdir=tmp -jar MuTect-1.1.4.jar --analysis_type MuTect --reference_sequence hg19_random.fa --dbsnp dbSNP137_chr.vcf --cosmic hg19_cosmic_OICR_v54_120711.vcf --input_file:tumor tumor.sorted.filtered.collapsed.merged.bam --input_file:normal blood.sorted.filtered.collapsed.merged.bam --intervals chrN:1-NNNNN --out chrom/MuTect.call_stats.chrN.txt --coverage_file chrom/MuTect.coverage.wig.chrN.txt --vcf chrom/MuTect.chrN.vcf
- vcf=`ls chrom/*vcf ; vcf-concat $vcf | vcf-sort > MuTect.allchrom.vcf
- VCF Annotation, filtering and merging:
- Vcf files from each caller were analyzed with custom scripts that ran Annovar (variant_function, exonic_variant_function) and appended this information into the info field:
- convert2annovar.pl --format vcf4 --includeinfo caller.vcf > caller.annovar; annotate_variation.pl -geneanno caller.annovar -buildver hg19 /path/to/annovar/humandb
- addAnnovarToVCF.pl (in house script)

MUTATION FILTRATION

SSM

The final merge utilized an in-house script that captured calls found in both Strelka and MuTect analyses. Some field names were reworked to allow inclusion from both sources. Headers were merged and annotated with source caller. Genotypes (GT) were taken from the MuTect SNV calls as Strelka does not provide these.

SIM

For indels called by Strelka, genotype calls were formed by review of the data.

COMPUTATIONAL RESOURCES

(no details specified)

MB.M

FASTQ PROCESSING

(no details specified)

MAPPING AND BAM PROCESSING

Alignment was performed with bwa 0.7.3a:

- bwa mem -t 24 -Ma -R '@RG\tID:513F-A_tumor1\tLB:513F-A_tumor\tSM:513F-A_tumor' /human_g1k_v37.fasta 513F-A_tumor1_1.fq.gz 513F-A_tumor1_2.fq.gz > 513F-A_tumor1.sam

Aligned sams are converted to bam format and sorted using samtools 0.1.19; duplicates were removed using picard-tools 1.51:

- samtools view -bS -@ 24 -T human_g1k_v37.fasta 513F-A_tumor1.sam -o 513F-A_tumor1_unsorted.bam
- samtools sort -m 5000000000 513F-A_tumor1_unsorted.bam 513F-A_tumor1
- samtools index 513F-A_tumor1.bam
- java -Xmx8g -jar MarkDuplicates.jar I=513F-A_tumor1.bam O=513F-A_tumor_dupRem.bam M=513F-

A_tumor_dup.metrics VALIDATION_STRINGENCY=SILENT ASSUME_SORTED=true REMOVE_DUPLICATES=true

- Using GATK version 2.1-13 we performed local realignment (RealignerTargetCreator and IndelRealigner) as well as base score recalibration (BaseRecalibrator and PrintReads):
- java -Xmx8g -jar GenomeAnalysisTK.jar -T RealignerTargetCreator -nt 24 -R human_g1k_v37.fasta -o 513F-A_realn.intervals -known:dbsnp,vcf dbsnp_137.b37.vcf -known:indels,vcf 1000g_indels.vcf -I 513F-A_tumor_dupRem.bam
- java -Xmx8g -jar GenomeAnalysisTK.jar -T IndelRealigner -rf NotPrimaryAlignment -R human_g1k_v37.fasta -targetIntervals 513F-A_realn.intervals -known:indels,vcf 1000g_indels.vcf -I 513F-A_tumor_dupRem.bam -o 513F-A_tumor_realign.bam
- java -Xmx8g -jar GenomeAnalysisTK.jar -T BaseRecalibrator -I 513F-A_tumor_realign.bam -rf BadCigar -R human_g1k_v37.fasta -knownSites:mask,vcf dbsnp_137.b37.vcf --default_platform illumina -l INFO -cov QualityScoreCovariate -cov CycleCovariate -cov ContextCovariate -cov ReadGroupCovariate --disable_indel_quals -o 513F-A_tumor_recal.csv
- java -Xmx8g -jar GenomeAnalysisTK.jar -T PrintReads -I 513F-A_tumor_realign.bam -rf BadCigar -l INFO -R human_g1k_v37.fasta -o 513F-A_tumor.bam -BQSR 513F-A_tumor_recal.csv

MUTATION CALLING

SSM

Mutation calls were made with MuTect 1.1.4:

- java -Xmx12g -jar MuTect-1.1.4.jar -T MuTect -I:normal 514_tumor.bam -I:tumor 513_tumor.bam -R human_g1k_v37.fasta --out 513_vs_514.vcf --dbsnp dbsnp_137.b37.vcf --cosmic b37_cosmic_v54_120711.vcf –enable_extended_output

SIM

SIMs were called jointly with the SSMs (see above).

MUTATION FILTRATION

SSM

The vcf files from MuTect were filtered using custom scripts that discarded positions based on read depth (>=10x for normal and >=14x for tumor), VAF in tumor (>=0.10) and VAF in normal (<=0.02).

SIM

Same as for SSM (see above).

COMPUTATIONAL RESOURCES

(no details specified)

MB.N

FASTQ PROCESSING

(no details specified)

MAPPING AND BAM PROCESSING

Alignment against the hg19 reference genome was done for each of the two samples using a set of custom perl scripts and perl libraries that implemented the following generalized commands (bwa 0.5.7, picard 1.92):

- bwa aln -t 4 -f R1.sai hg19.random.fa R1.fastq.gz / bwa aln -t 4 -f R2.sai hg19.random.fa R2.fastq.gz
- bwa sampe -f lane.sam hg19.random.fa R1.sai R2.sai R1.fastq.gz R2.fastq.gz
- java -Xmx16g -jar AddOrReplaceReadGroups.jar RGCN=oicr.on.ca RGLB=library RGPL=platform RGPU=none RGSM=sample INPUT=lane.sam OUTPUT=lane.rg.bam VALIDATION_STRINGENCY=LENIENT SO=coordinate

- java -Xmx16g -jar MergeSamFiles.jar INPUT=lane1.rg.bam ... INPUT=laneN.rg.bam OUTPUT=sample.merged.bam SORT_ORDER=coordinate ASSUME_SORTED=no USE_THREADING=yes VALIDATION_STRINGENCY=LENIENT
- Index BAM files using picard

BAM Processing (GATK 2.4.9, Picard 1.92):
- Indel realingment and base recalibration using GATK
- Merge the GATK processed normal and tumor BAM files using Picard
- Index the merged BAM files using Picard

MUTATION CALLING

SSM

SSM calls were made with Strelka (1.0.12).
Strelka config.ini contains the following values (used the config file for BWA sampe and isSkipDepthFilters was turned off):
- binSize = 25000000
- depthFilterMultiple = 3.0
- extraStrelkaArguments =
- indelMaxIntHpolLength = 14
- indelMaxRefRepeat = 8
- indelMaxWindowFilteredBasecallFrac = 0.3
- isSkipDepthFilters = 1
- isWriteRealignedBam = 0
- maxInputDepth = 10000
- minTier1Mapq = 20
- minTier2Mapq = 5
- sindelNoise = 0.000001
- sindelPrior = 0.000001
- sindelQuality_LowerBound = 30
- snvMaxFilteredBasecallFrac = 0.4
- snvMaxSpanningDeletionFrac = 0.75
- ssnvNoise = 0.0000005
- ssnvNoiseStrandBiasFrac = 0.5
- ssnvPrior = 0.000001

We added GT in the FORMAT field and genotype in the NORMAL and TUMOR fields of the vcf files based on SGT in INFO field.

SIM

Same as for SSM (see above).

MUTATION FILTRATION

SSM

Whitelist:
- COSMIC (mutations that have occured in > 1 sample)

Blacklist:
- dbSNP137
- 1kGenomes
- Inhouse failed-to-validate blacklist
- Fuentes 2012
- Encode Dac/Duke
- Duplicate gene database.

COMPUTATIONAL RESOURCES

(no details specified)

MB.O

FASTQ PROCESSING

Lane Quality control:
Each lane is checked with our quality control pipeline. All lanes were kept to create the sample bamfile.

MAPPING AND BAM PROCESSING

Alignment:

- Core mapping with BWA v0.7.5a
    - bwa aln -l 32 -t 6 -q 20 (6 CPUs used)
    - bwa sampe -P

Presently 'bwa sampe' is called within a Perl wrapper and pairs with both reads unmapped are written to a separate BAM file.
Bam processing:

- Sorting + indexing lane BAM file (Picard tools v1.73)
- Indel realignment at known sites (GATK2 v2.0-23)
- Duplicates Marking (Picard)
- Recalibration (GATK2): Calculating table and generating recalibrated BAM file
- Picard MergeSamFile
- Picard Markduplicates (with remove duplicates option)
- Picard and GATK are used with standard parameters.

In step picard MergeSamFile, we merged all the lanes that passed the quality controls. We then removed the duplicates to obtain a BAM file at the sample level.
The final estimated coverage were : 28x for the normal sample and 38x for the tumor sample

MUTATION CALLING

SSM

We called somatic mutations with MuTect (version 1.1.4, Broad tool). This tool compares normal and tumor samples to retrieve somatic mutations.
Default values for all parameters were used.

SIM

We used SomaticIndelDetector, a tool included in GATK2 to call small indels.

MUTATION FILTRATION

SSM

Among MuTect calls, 40064 candidate calls were retained. These candidate mutations were then filtered according to these filters:

- TAC : minimum number of read carrying mutant allele
- DP : read depth + base quality filter
- MQ : mapping quality of reads carrying mutant allele
- PB : read position filter
- SB : strand bias filter
- GL : germline filter
- RE : mutations found nearby repeat regions
- BL : mutant allele fount in satellite regions
- CU2 : the presence of the mutant allele in a panel of 18 other germline samples sequenced with Illumina protocol

SIM

Calls were further filtered by our pipeline:
- min number of reads carrying indel
- mapping quality of reads carrying indel
- germline filtering
- strand bias filtering
- mismatches in mapping of reads carrying indel (number and windows around indel filtering)
- base quality around indel
- the position of indels within boundaries of known repeated, centromeric and telomeric regions
- mutation filtering (indel starts at a SNV position)
- position of indels in the carrying reads
- the presence of the indel in a panel of 20 other germline samples sequenced with Illumina protocol

COMPUTATIONAL RESOURCES

Almost the same as described in CLL.O1.

MB.P

Same pipeline as CLL.P (above)

--

MB.Q

FASTQ PROCESSING

(no details specified)

MAPPING AND BAM PROCESSING

Quality assessed fastq pair files aligned with BWA bwa 0.6.2-mt
- BWA options: bwa aln –t ${threads}
- sampe options: bwa sampe -t ${threads} –r {set readgroup}
- samtools-0.1.19 for sorting and mapset BAM creation
- mark duplicates: Picard MarkDuplicates Version: 1.97
- fix BAM headers qBAMFIX (in house tool)
- Merge mapset BAMs qBAMMerge (in house tool)
- mark duplicates: Picard MarkDuplicates Version: 1.97
- QC of final and mapest level BAMs by qProfiler and qCoverage (in house tools)

MUTATION CALLING

SSM

qSNP:

- Input: matched tumor, normal BAM and reference
- Filter qBAMFilter (in house tool) options
    - Primary alignment
    - not unmapped
    - Cigar_M > 35
    - option_SM > 14
    - MD_mismatch < 3
    - Flag_DuplicateRead == false
    - Identification of candidate mutations through rules based annotation

GATK:

- GATK v 1.6, Unified Genotyper
- Input: matched tumor and normal BAMs separately
    - Primary alignment only
    - Not unmapped or vendor failed
    - Flag_DuplicateRead == false
    - Subtractive comparison for somatic/ germline assignation

SIM

- Pindel v 0.2.4
- Input: matched tumor and normal BAMs separately
- Subtractive comparison for somatic/ germline assignation
- Annotation Repeat masker for simple repeats; low complexity; satellite regions

MUTATION FILTRATION

SSM

- Supporting reads with novel starts
- Supporting counts for each strand
- Coverage in normal and tumor
- Presence of mutation in unfiltered normal BAM
- Annotation with dbSNP 135 and In house germline database
- Gene model Ensemble v70 consequence annotation
- Filtering and ranking (qMAFTools) based on annotation

Intersection and *in silico* verification

- Pileup of mutation positions qBASEPileup in other matched available data (e.g. RNA_seq)
- Intersection of qSNP and GATK mutations plus mutations unique to either qSNP and GATK but with supporting evidence in another matched dataset.

SIM

- Supporting reads with novel starts
- Supporting counts for each strand
- Supporting clips
- Coverage in normal and tumor
- Presence of mutation in unfiltered normal BAM
- Homopolymer length
- Annotation with In house germline database
- Gene model Ensemble v70 consequence annotation
- Filtering and ranking (qMAFTools) based on annotation

COMPUTATIONAL RESOURCES

(no details specified)

Supplementary References

1 Alexandrov, L. B. *et al.* Signatures of mutational processes in human cancer. *Nature* **500**, 415-421, doi:10.1038/nature12477 (2013).

2 Puente, X. S. *et al.* Whole-genome sequencing identifies recurrent mutations in chronic lymphocytic leukaemia. *Nature* **475**, 101-105, doi:10.1038/nature10113 (2011).

3 Louis, D. N. *et al.* The 2007 WHO classification of tumours of the central nervous system. *Acta neuropathologica* **114**, 97-109, doi:10.1007/s00401-007-0243-4 (2007).

4 Taylor, M. D. *et al.* Molecular subgroups of medulloblastoma: the current consensus. *Acta neuropathologica* **123**, 465-472, doi:10.1007/s00401-011-0922-z (2012).

5 Jones, D. T. *et al.* Dissecting the genomic complexity underlying medulloblastoma. *Nature* **488**, 100-105, doi:10.1038/nature11284 (2012).

6 Li, S. *et al.* Detecting and correcting systematic variation in large-scale RNA sequencing data. *Nat Biotech* **32**, 888-895, doi:10.1038/nbt.3000 (2014).

7 Futreal, P. A. *et al.* A census of human cancer genes. *Nature reviews. Cancer* **4**, 177-183, doi:10.1038/nrc1299 (2004).

8 Ross, M. G. *et al.* Characterizing and measuring bias in sequence data. *Genome biology* **14**, R51, doi:10.1186/gb-2013-14-5-r51 (2013).

9 Li, H. *et al.* The Sequence Alignment/Map format and SAMtools. *Bioinformatics* **25**, 2078-2079, doi:10.1093/bioinformatics/btp352 (2009).

10 Koboldt, D. C. *et al.* VarScan 2: somatic mutation and copy number alteration discovery in cancer by exome sequencing. *Genome Res* **22**, 568-576, doi:10.1101/gr.129684.111 (2012).

11 Cingolani, P. *et al.* A program for annotating and predicting the effects of single nucleotide polymorphisms, SnpEff: SNPs in the genome of Drosophila melanogaster strain w1118; iso-2; iso-3. *Fly (Austin)* **6**, 80-92, doi:10.4161/fly.19695 (2012).

12 Moncunill, V. *et al.* Comprehensive characterization of complex structural variations in cancer by directly comparing genome sequence reads. *Nat Biotechnol* **32**, 1106-1112, doi:10.1038/nbt.3027 (2014).

13 Larson, D. E. *et al.* SomaticSniper: identification of somatic point mutations in whole genome sequencing data. *Bioinformatics* **28**, 311-317, doi:10.1093/bioinformatics/btr665 (2012).

14 Ye, K., Schulz, M. H., Long, Q., Apweiler, R. & Ning, Z. Pindel: a pattern growth approach to detect break points of large deletions and medium sized insertions from paired-end short reads. *Bioinformatics* **25**, 2865-2871, doi:10.1093/bioinformatics/btp394 (2009).

15 Wang, J. *et al.* CREST maps somatic structural variation in cancer genomes with base-pair resolution. *Nat Methods* **8**, 652-654, doi:10.1038/nmeth.1628 (2011).

16 Stephens, P. J. *et al.* The landscape of cancer genes and mutational processes in breast cancer. *Nature* **486**, 400-404, doi:10.1038/nature11017 (2012).

17 Greenman, C. D. *et al.* PICNIC: an algorithm to predict absolute allelic copy number variation with microarray cancer data. *Biostatistics* **11**, 164-175, doi:10.1093/biostatistics/kxp045 (2010).
